# Rapid Lewis Acid Screening and Reaction Optimization Using 3D-Printed Catalyst-Impregnated Stirrer Devices in the Synthesis of Heterocycles

**DOI:** 10.1021/acs.joc.3c01601

**Published:** 2023-11-27

**Authors:** Rumintha Thavarajah, Matthew R. Penny, Ryo Torii, Stephen T. Hilton

**Affiliations:** † Department of Pharmaceutical and Biological Chemistry, UCL School of Pharmacy, 29-39 Brunswick Square, London WC1N 1AX, U.K.; ‡ Department of Mechanical Engineering, UCL, Torrington Place, London WC1E 7JE, U.K.

## Abstract

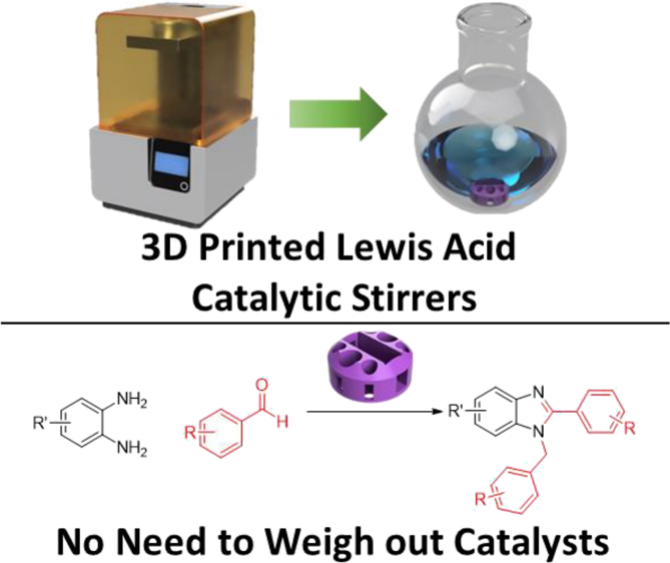

We describe the development of Lewis acid (LA) catalyst-impregnated 3D-printed stirrer devices and demonstrate their ability to facilitate the rapid screening of reaction conditions to synthesize heterocycles. The stereolithography 3D-printed stirrer devices were designed to fit round-bottomed flasks and Radleys carousel tubes using our recently reported solvent-resistant resin, and using CFD modeling studies and experimental data, we demonstrated that the device design leads to rapid mixing and rapid throughput over the device surface. Using a range of LA 3D-printed stirrers, the reaction between a diamine and an aldehyde was optimized for the catalyst and solvent, and we demonstrated that use of the 3D-printed catalyst-embedded devices led to higher yields and reduced reaction times. A library of benzimidazole and benzothiazole compounds were synthesized, and the use of devices led to efficient formation of the product as well as low levels of the catalyst in the resultant crude mixture. The use of these devices makes the process of setting up multiple reactions simpler by avoiding weighing out of catalysts, and the devices, once used, can be simply removed from the reaction, making the process of compound library synthesis more facile.

## Introduction

Additive manufacturing (AM),^1,2^ also known as three-dimensional (3D) printing, is a versatile technique by which complex 3D objects can be created from a digital design with precise geometry.^3^ Over the past decade, 3D printing has been established as a revolutionary tool for the chemical and pharmaceutical industries and other scientific disciplines.^1−9^ The technique of 3D printing has grown in the field of chemistry following research by ourselves and others, where it has been shown to be an essential tool for the design, development, and production of low-cost laboratory equipment, continuous flow systems, and teaching aids.^1,3,8−11^

Despite the clear advantages of stereolithography (SLA) 3D printing over fused deposition modeling in terms of accuracy and reproducibility, the use of SLA 3D printing in chemistry remains limited. This in part stems from the paucity of solvent-resistant commercial resins that are available for SLA printing.^1^ However, recent research by our group into catalyst-embedded stirrer devices for chemical synthesis has led to the discovery of a resin formulation that is stable to a range of organic solvents and that can be 3D printed with embedded Pd catalysts and that was shown to efficiently catalyze Suzuki–Miyaura reactions with low catalyst loss.^12^

**Figure 1:**
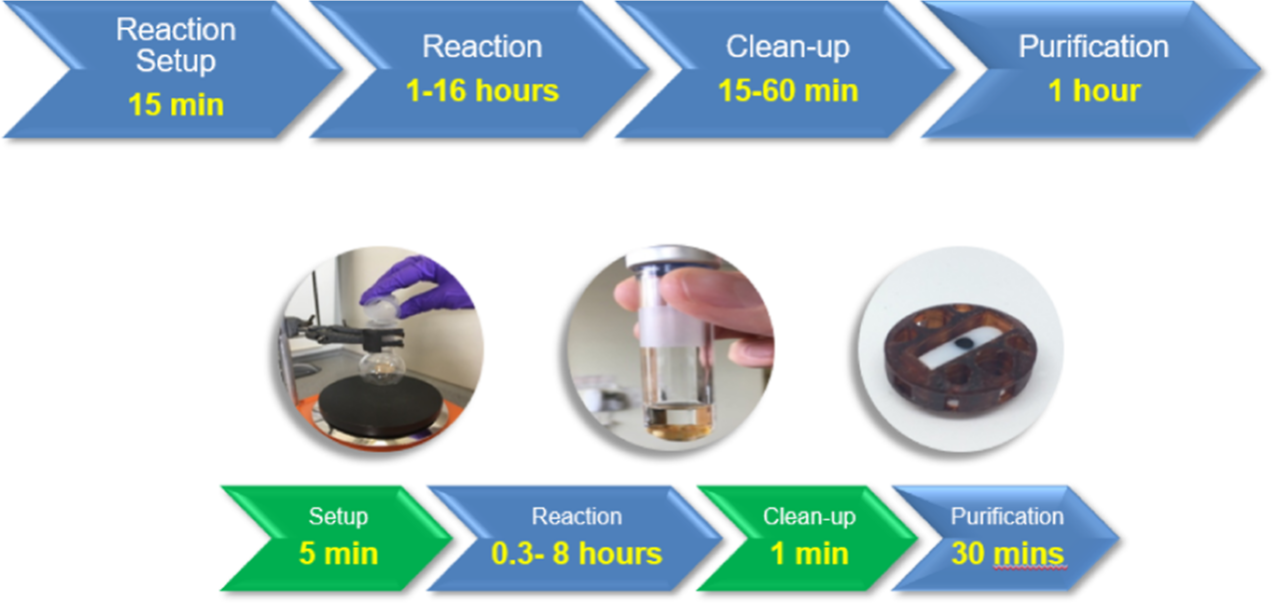
Illustration of standard chemical workflow (top) vs our approach via the use of 3D-printed catalyst-embedded stirrer devices (bottom).

As a result of our research into 3D printing and 3D-printed catalyst-embedded stirrers, we were intrigued by the possibility of extending our research into the area of Lewis acids (LAs), where LA catalysts could be incorporated into SLA 3D-printed stirrer devices. Solid-supported LAs have previously been used in the synthesis of heterocycles and active pharmaceutical ingredients,^13^ but while they have been used in this approach, they typically require weighing out before use, in much the same way as the use of traditional solution-based catalysts.^13^ Our new paradigm approach therefore provides a much more simplified workflow,^12^ where a range of LA-impregnated stirrers can be readily added to a reaction followed by the reagents. Once the reaction is complete, they can then be removed at the end of the reaction in much the same way as a stir bar, making the entire process much simpler to follow (Figure 1).

**Figure 2:**
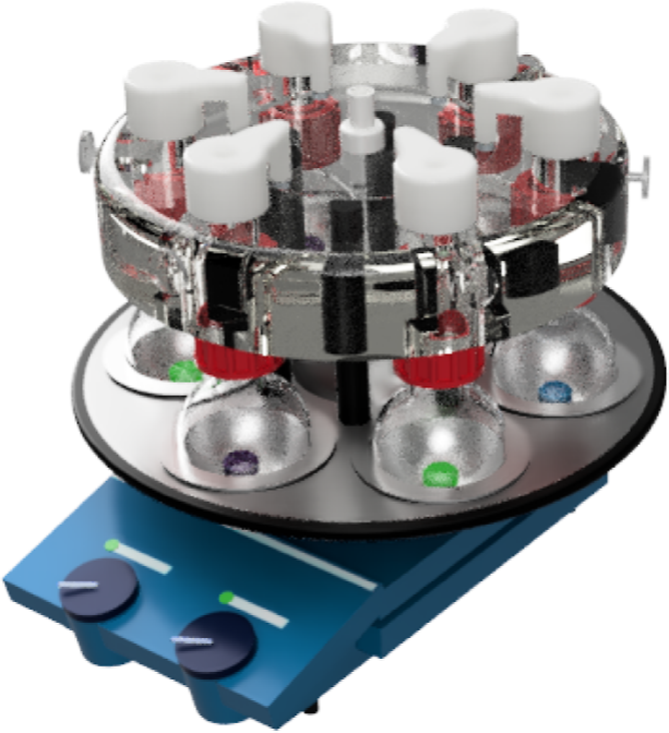
CAD drawing illustration of the use of a range of six different LA-impregnated devices (colored) in a Radleys carousel reactor with the top made opaque for clarity.

As a proof of concept, we were interested in applying our approach using 3D-printed LA-impregnated stirrer beads to the synthesis of benzimidazoles and benzothiazoles and related derivatives due to their potent biological and pharmaceutical properties.^14^ Both of these key heterocycles and related derivatives display numerous therapeutic activities such as: anticancer,^15^ antifungal,^16^ antiflammatory,^17,18^ antimicrobial,^19^ antiviral,^20,21^ anti-HIV,^22^ antibacterial,^23^ and antiulcer effects.^24^ The aim of our approach, was to demonstrate that the stirrer devices containing a range of catalysts, could then be used in a Radleys carousel to optimize the reaction scope for both catalysts and solvents, simplifying the workflow in catalyst screening.^25^ In this manner, we would therefore be able to carry out the reaction with 6 or 12 stirrer devices at any one time, without the need to weigh out the catalyst (Figure 2).

## Results and Discussion

**Figure 3:**
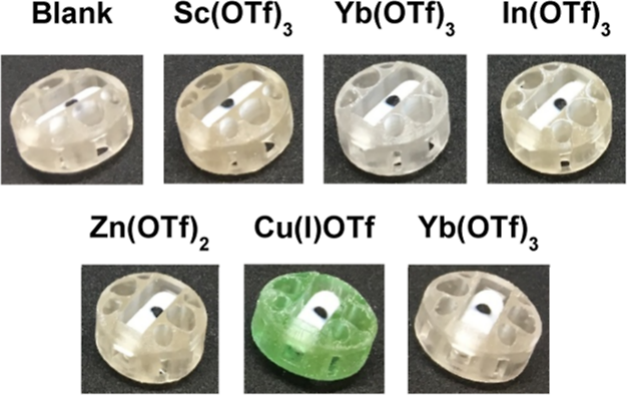
3D-printed stirrer devices containing LAs.

To exemplify our approach, we explored the 3D printing of a range of LA catalyst containing devices, covering scandium, ytterbium, indium, zinc, copper (I), and yttrium. Catalysts 2.5% (*w*/*w* versus resin) were dissolved in the poly(ethylene glycol) diacrylate (PEGDA) monomer, photoinitiator added, and the resultant devices 3D printed on a Formlabs Form1+ 3D printer to give the resultant catalyst-embedded devices and rare earth stirrer beads inserted into the central cavity of the device as shown (Figure 3 and Supporting Information). The weight and the amount of the catalyst in each 3D-printed stirrer device were calculated from 3D printing of the devices in triplicate, with an average weight range of 770–940 mg and a catalyst loading ranging from 19–23 mg depending on the catalyst (Supporting Information). There is a slight variation in the standard error of the mean of less than 0.5% for all catalysts examined, suggesting good size uniformity of the 3D-printed stirrer devices. It is also evident from the uniform green color of the CuOTf-impregnated stirrer device that there was successful dispersion of the catalyst throughout the device in all cases in which the catalysts were soluble in the 3D printing resin.

**Figure 4:**
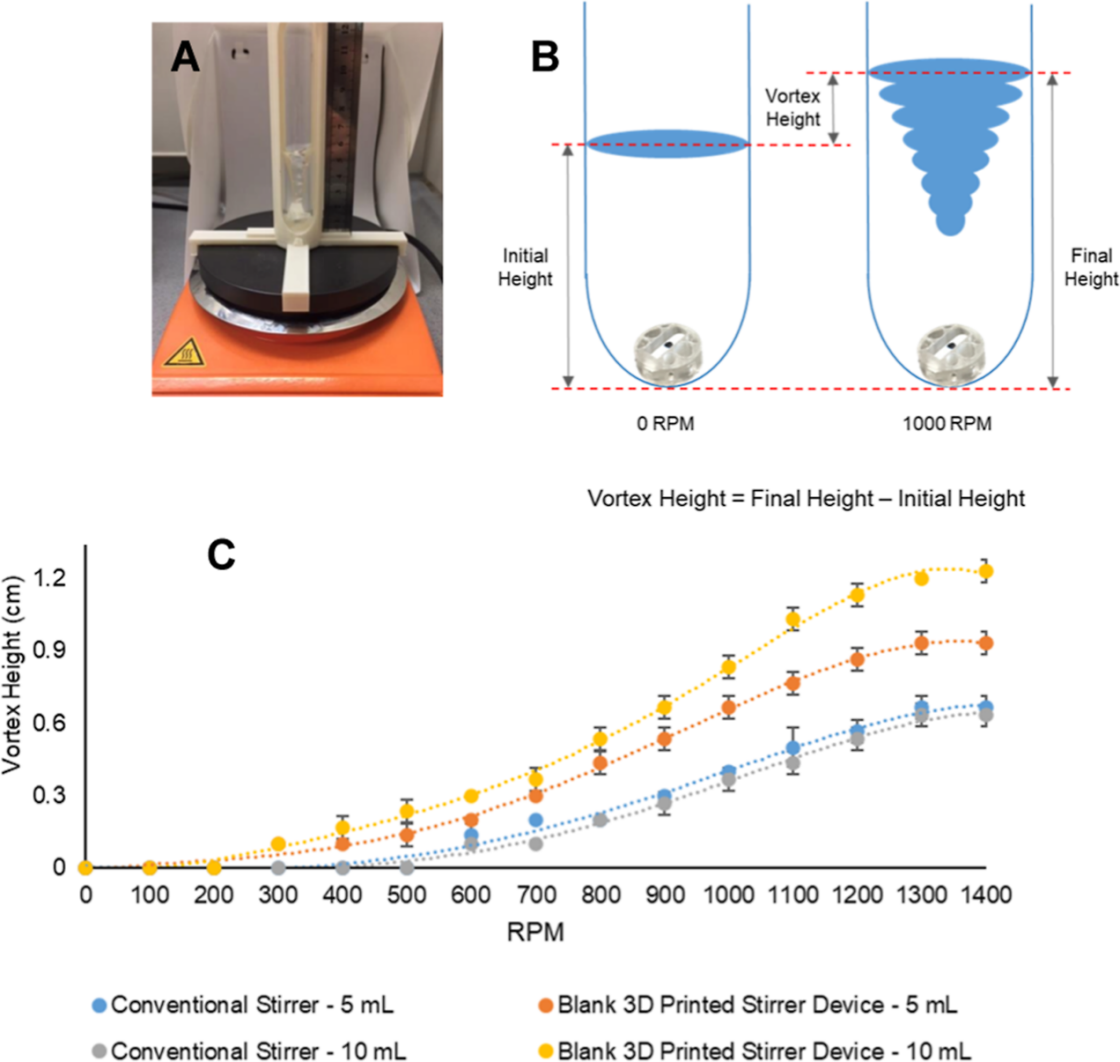
Investigation into the stirring effects. (A) Physical set up for the vortex measurement. (B) Illustration of how the vortex height was calculated. (C) Direct comparison of the vortex capabilities of a conventional stirrer and a blank 3D-printed carousel/RBF stirrer device in 5 and 10 mL of EtOH using a carousel vial (20 mL).

In order to test the efficacy of our 3D-printed stirrer devices, initial investigations focused on exploring the effect of stirring, which was conducted through measuring the vortex height capabilities of each device. A Radleys carousel vial was placed in a 3D-printed vial holder with a ruler set up on a stirrer hotplate to ensure that the vial was kept in the middle of the stirrer hot plate so that no variation in both the magnetic field and in the height of the vial occurred during repeat runs (Figure 4A). During this analysis, EtOH (5 and 10 mL) was placed in the carousel vial along with a blank 3D-printed stirrer device and the resultant vial placed in the 3D-printed holder containing a ruler. This was placed on a stirrer hot plate, and the initial height measured from the bottom of the carousel tube to the top of the solvent level. While the mixture was stirred at each rpm, the final height was measured from the bottom of the carousel tube to the top of the solvent level. The difference in the final height and the initial height was measured as the vortex height (Figure 4B).

**Figure 5:**
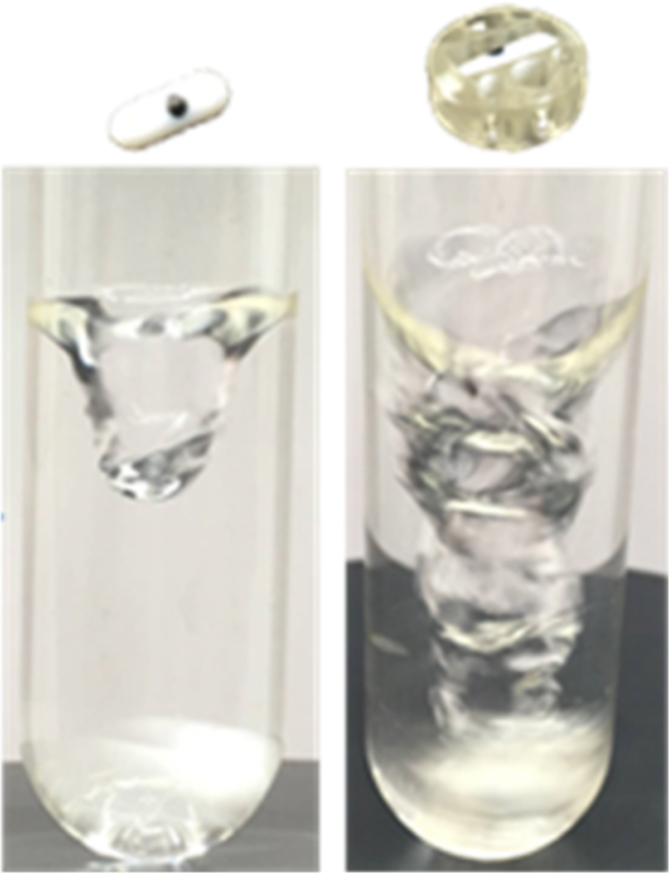
Vortexing ability of a conventional stirrer at 1400 rpm (left) versus that of its 3D-printed congener (right).

The stirring ability of the 3D-printed stirrer device greatly exceeded that of the conventional stirrer with both 5 and 10 mL of solvent (Figure 4C). The vortex height of both stirrers remained similar from 0 to 200 rpm, however, from 300 rpm, the difference in vortex height of the 3D-printed stirrer devices steadily increased, while the conventional stirrer remained at zero until 500 rpm. Increasing the volume of EtOH to 10 mL led to a steeper increase in turbulence. The high vortex abilities measured for the 3D-printed stirrer over that of its simple bar congener device can be visualized below, showing the increased turbulence exerted by the device (Figure 5).

**Figure 6:**
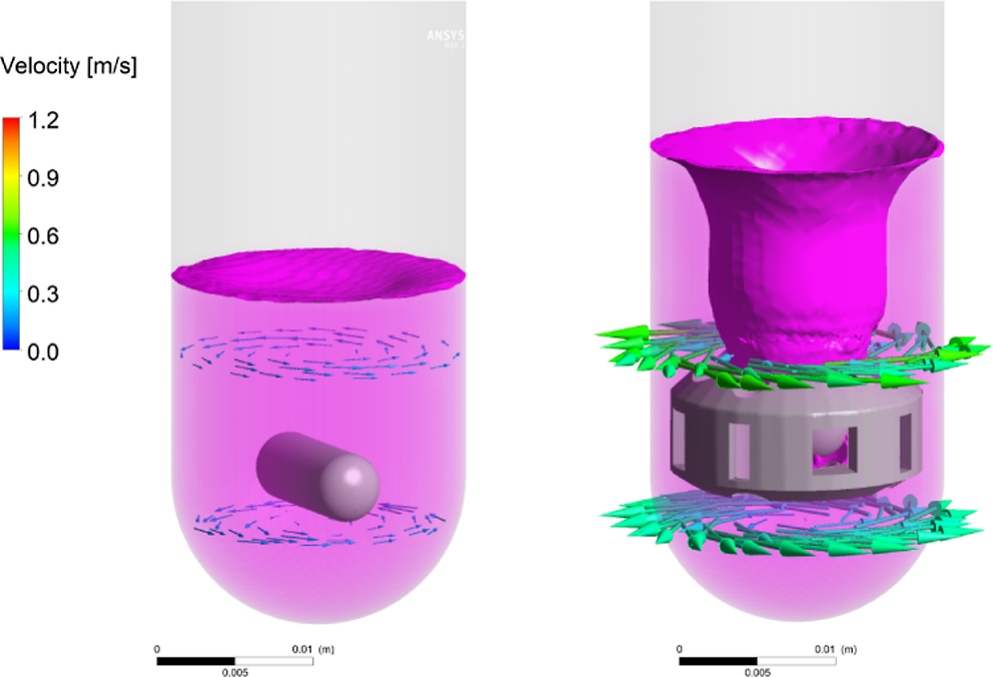
Computational analysis of the vortexing ability of a conventional stirrer at 1400 rpm (left) versus that of its 3D-printed congener (right).

To corroborate the vortexing height capability findings, a modeling study comparing the mixing ability of a blank 3D-printed stirrer device against a conventional magnetic stirrer was conducted using EtOH (5 mL) with a density of 0.7893 g/cm^3^ and a viscosity of 1.074 mPa s. At time equal to zero, the temperature of the air was set to 25 °C with no observed surface tension at the interface. The results from the simulation of simplified fluid dynamics clearly agreed with the experimental findings, in that the 3D-printed device displays a higher degree of rapid mixing with greater turbulence (Figure 6 and Supporting Information).

**Figure 7:**
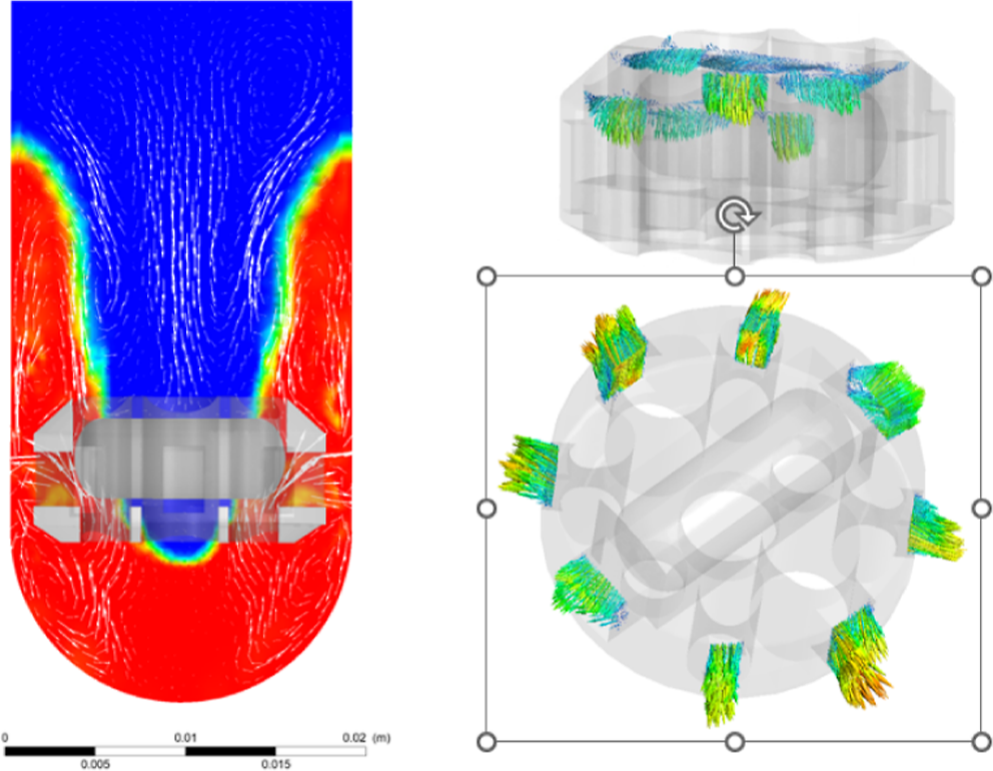
Modeling of the vortexing ability of the 3D-printed stirrer and an illustration of the fluid flow through the device.

The average swirling flow velocity magnitude in the two planes at *t* = 5 s is greater in the 3D-printed stirrer device than that in the conventional stirrer. The velocity for the 3D-printed stirrer device on the top and bottom plane is 0.0234 and 0.407 m/s, respectively, which is 2.5 times greater than the velocity experienced with the conventional stirrer. The flow is sucked in from the region below and ejected sideways through the lateral opening (Figure 7 and Supporting Information). The color contour represents the phases, where red is EtOH and blue represents the air. The volumetric flow rates coming through different openings are discrete; the green openings have the lowest flow rate of 0.212 mL/s, but the red and side openings have the highest flow rate of 1.48 and 1.13 mL/s, respectively. The total flow through the 3D-printed stirrer device is 12.5 mL/s. Therefore, it shows that the 3D-printed stirrer device does indeed exhibit an increased degree of mixing and greater turbulence in comparison with the commercially available conventional bar stirrer.

**Scheme 1:**
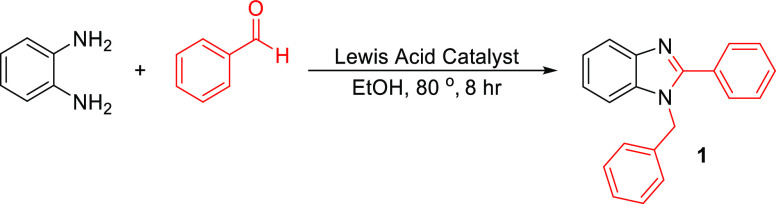
Reaction of Benzene-1,2-diamine and Benzaldehyde in the Presence of Various LA Catalysts in EtOH

Following the results of the mixing tests in the carousel tubes, we wanted to explore the ability of the impregnated catalysts in the formation of substituted benzimidazoles following a report by Fan et al. On the use of LAs to facilitate the formation of benzimidazoles, we wanted to show how we could quickly and efficiently improve the reaction using our approach (Scheme 1).^26^

The 3D-printed LA catalyst-impregnated devices screened in our study were: Sc(OTf)_3_, Yb(OTf)_3_, In(OTf)_3_, Zn(OTf)_2_, Sc(OTf)_3_, CuOTf, and Y(OTf)_3_. The screened catalysts were heated at reflux in Radleys carousel tubes in EtOH for 8 h under inert conditions (Scheme 1). An initial background reaction with no catalyst and a conventional magnetic stirrer bar was run as a control and gave a low yield of the product (6%). Surprisingly, when the control reaction was repeated using a blank 3D-printed stirrer, a yield of 14% was achieved. We attributed this increase in the yield to the rapid mixing abilities of the 3D-printed stirrer devices in comparison to the conventional magnetic stirrer bar (Table 1).

**Table 1:** A = Conventional Stirrer + Powdered Catalyst (0.1 mmol, 0.049 g) B = Blank 3D-Printed Stirrer Device + Powdered Catalyst (0.1 mmol, 0.049 g) C = Lewis Acid Catalyst-Impregnated 3D-Printed Stirrer Device^a^

		form of catalyst					
		A		B		C	
isolated yield (%)	normal stirrer	6**					
	blank 3D stirrer	14**					
	Sc(OTf)_3_	61	62*	64	65*	78	74*
	Yb(OTf)_3_	37	54	71			
	In(OTf)_3_	49	63	65			
	Zn(OTf)_2_	38	53	57			
	CuOTf	25	33	38			
	Y(OTf)	23	25	34			

a * = RBF, and ** = no catalyst added.

From the results, we can clearly see that Sc(OTf)_3_ proved to be the best catalyst for the synthesis of 2,3-disubstituted benzimidazoles and that in all cases, the use of the 3D-printed catalyst-impregnated device gave the highest yields in all the reactions despite the fact that the amount of the catalyst in the catalyst-impregnated stirrer device is a lot less in comparison to the powdered catalyst used (∼20 mg versus ∼48 mg). The stirrers possess a surface area of 1266 mm^2^ and a volume of 646 mm^3^, giving a surface area/volume ratio of 2.0 mm^–1^. However, the catalyst itself is distributed evenly throughout the device, meaning that only the catalyst near the surface is available for reaction.^12^ As such, we estimate that only 10% of the actual catalyst is available for the reaction for the carousel stirrer devices. The Sc(OTf)_3_-impregnated stirrer device has approximately 20 mg of Sc(OTf)_3_, whereas in the reaction 49 mg has been used as a powdered catalyst. The reaction with Sc(OTf)_3_ was also repeated in a round-bottomed flask (RBF) to further investigate whether similar yields can be achieved in a different vessel, and we were pleased to note that this was the case.

It is worth mentioning that the workup procedure was obviated in the reactions performed using the catalytic devices as the 3D-printed catalyst-embedded device could be simply removed from the reaction mixture upon completion, whereas workup was mandatory in the reactions catalyzed by powdered LA catalysts.

A solvent screening test was subsequently carried out using scandium triflate-impregnated 3D-printed stirrer devices. From the results, acetonitrile was found to be the optimum solvent, giving the highest yield and the shortest reaction times (Table 2). Pleasingly, the use of polar and nonpolar solvents did not affect the architecture of stirrer devices, with the structural integrity maintained even at temperatures of 100 °C.

**Table 2:** Screening of Polar and Nonpolar Solvents to Optimize the Reaction Conditions

solvent	temperature of reaction (°C)	time of reflux (h)	isolated yield (%)
MeCN	80	2	87
MeCN	80	4	84
MeCN	80	6	79
MeCN (dry)	80	6	71
MeCN + H_2_O (4:1)	80	6	52
EtOH	80	2	51
EtOAc	80	6	55
MeOH (dry)	80	6	82
*t*-butanol	80	6	68
IPA	80	6	65
H_2_O	100	6	42
THF (dry)	80	6	27
toluene	100	6	63

**Scheme 2:**
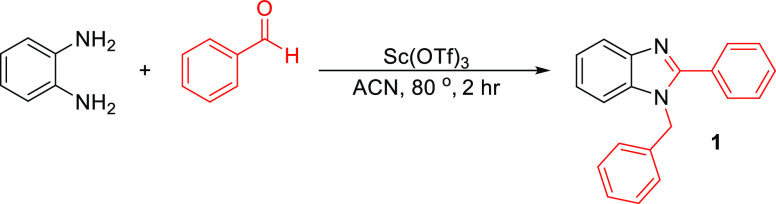
Optimized Reaction Condition for the Synthesis of 1-Benzyl-2-phenyl-1*H*-benzo[*d*]imidazole Using Benzene-1,2-diamine, Benzaldehyde and Sc(OTf)_3_ as the Catalyst in MeCN

Following the selection of the optimized conditions, comparative reactions were carried out to try to understand the relative advantages of the stirrer devices as opposed to normal stirrers. Reactions without any catalyst using both conventional and blank 3D-printed stirrer devices gave 0% yield at the end of the reaction. We calculated that there is 20.3 mg of Sc(OTf)_3_ in each device, so a comparative reaction was also carried out using 20.3 mg of the powdered catalyst, which gave a 63% yield. Use of the catalyst-impregnated stirrer device gave the highest yield of 87% (Scheme 2, Table 3).

**Table 3:** Isolated Yields Were Obtained from Different Forms of Catalysts

form of catalyst	% isolated yield
normal stirrer	0
3D blank stirrer	0
normal stirrer + 0.049 mg cat	68
normal stirrer + 0.020 mg cat	63
3D blank stirrer + 0.049 mg cat	79
3D blank stirrer + 0.020 mg cat	76
3D Sc(OTf)_3_-impregnated stirrer	87

A reusability test was carried out using a Sc(OTf)_3_ impregnated 3D-printed stirrer device. The device was washed in the reaction solvent, dried, and used in the same reaction using the same substrate and reaction molarities. The yields of the reaction are consistent for the first two repeats: 83 and 86% respectively, with yields dropping from the third repeat (Table 4 and Supporting Information).

**Table 4:**
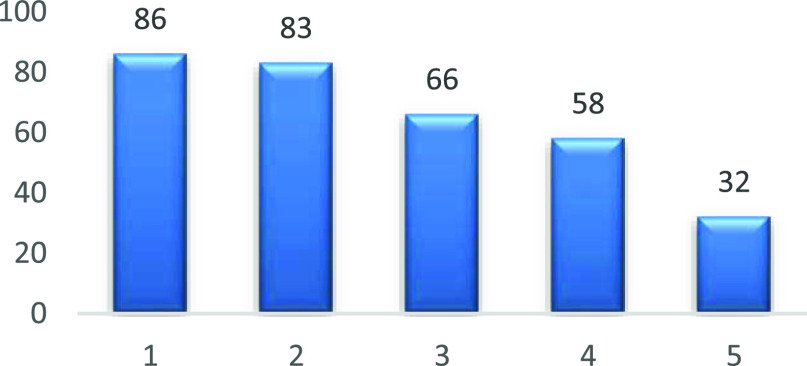
Reusability Test with a Sc(OTf)_3_-Impregnated Stirrer

From the results (Supporting Information), it was clear that the devices discolor rapidly after the second reaction presumably upon exposure to the diamine, with increasing morphological changes to the device after each repeat.

To understand whether the scandium was being lost to the reaction through leaching and catalyzing the reaction in that manner, or whether the reaction was taking place at the surface of the device, an analysis of scandium leaching from the reaction was carried out, with detection from inductively coupled plasma optical emission spectroscopy (ICP-OES). Pleasingly, only 1% of the total amount of the scandium catalyst was lost in the two-hour reaction when the catalyst-impregnated 3D-printed stirrer device was utilized (Table 5). This reduced leaching effect of the 3D-printed stirrer devices and the fact that these devices can be reused indicate that it may not be a leaching effect and may also partly be due to surface phenomena. Therefore, it is safe to assume that it is not the leached material that is carrying out the reaction.

**Table 5:** ICP–MS Study of Scandium Leaching in the Reaction; (A) No Catalyst + Blank 3D-Printed Stirrer Device, (B) Powdered Catalyst + Conventional Magnetic Stirrer, (C) Powdered Catalyst + Blank 3D-Printed Stirrer Device, and (D) Sc(OTf)_3_-Impregnated 3D-Printed Stirrer Device

entry	mass of Sc metal used in the reaction (mg)	mass of Sc metal detected through ICP–MS (mg)	Sc(OTf)_3_ leached (%)
A	0.00	0.00	0
B	4.48	3.98	89
C	4.48	3.67	82
D	1.85	0.02	1

In order to demonstrate the utility of our approach, using the optimized reaction conditions, a library of benzimidazole compounds were synthesized using the scandium triflate-impregnated 3D-printed stirrer device in excellent yields (Table 6). A range of diamine and aldehyde derivatives were chosen to investigate the level of tolerance of the stirrer devices to different functional groups.

**Table 6:**
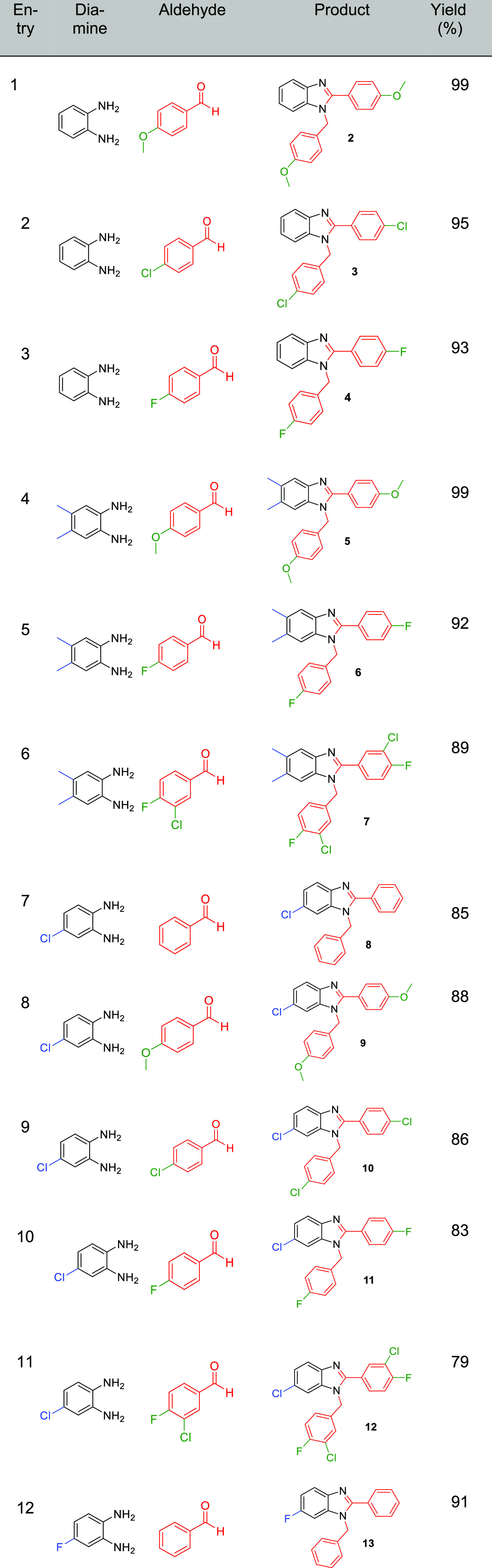
Library of Benzimidazole Compounds Using Derivatives of Diamines and Aldehydes

Pleasingly in all cases, the resultant benzimidazoles were obtained in good to excellent yields, demonstrating the tolerability of the devices toward an array of functional groups.

**Scheme 3:**
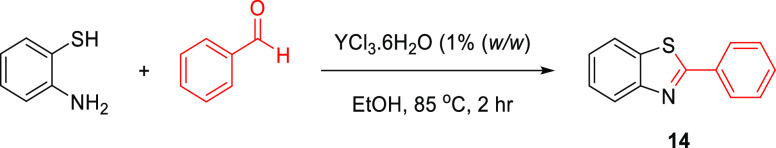
Synthesis of 2-phenylbenzo[*d*]thiazole, YCl_3_·6H_2_O Catalyzed *o*-Aminothiophenol and Benzaldehyde in EtOH

Having demonstrated the utility and application of the LA catalyst-embedded devices in optimizing reaction conditions, we next wanted to explore their utility in a previously reported LA-catalyzed reaction. Fan et al. recently reported on the use of yttrium chloride to catalyze the reaction between *o*-aminothiophenol and benzaldehyde in the synthesis of benzthiazoles.^27^ We were therefore interested in using YCl_3_ as the catalyst in our resin formulation. However, due to the insoluble nature of YCl_3_ in our resin formulation, we elected to use YCl_3_·6H_2_O. A loading of 1% catalyst in the stirrer device was chosen to maintain consistency with powdered catalysts, enabling direct comparisons between the two variations of catalysts (Scheme 3).

**Figure 8:**
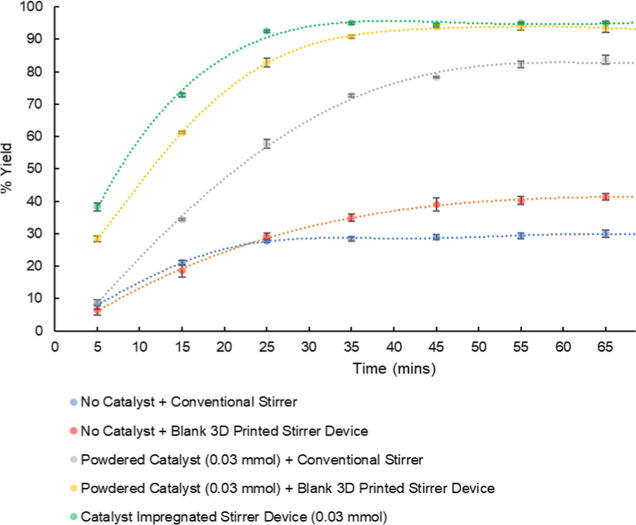
Monitoring the progress of the reaction in the formation of 2-phenylbenzo[*d*]thiazole via LCMS analysis.

We were interested in monitoring the progress of the above reaction using a 1% YCl_3_·6H_2_O-impregnated stirrer device (including % conversion over the course of the reaction) to explore the potential of our 3D-printed stirrers. As such, we carried out a series of reaction runs using conventional stirring, powdered catalyst, and catalyst-impregnated devices, with all reactions carried out in triplicate, and the results of the reaction and the reaction profiles are shown below (Figure 8).

Both control reactions, without the use of any catalyst, showed conversion of the product with 42 and 30% yield with the blank 3D printer stirrer device (red line) and conventional stirrer (blue line), respectively, in 65 min. The significant difference between these two reactions highlights the effective mixing achieved with the 3D printer stirrer device. The reaction using the catalyst-impregnated stirrer device (green line) went to completion at 35 min in a yield of 95%. In comparison to all other reactions using different forms of catalysts, the catalyst-impregnated stirrer device exhibited the fastest reaction rate and highest yield in the shortest time. Furthermore. a similar trend in the rate of reaction was observed when using the powdered catalyst in combination with the blank 3D-printed stirrer device (yellow line) but with a slight increase in reaction time to 45 min. This close relationship is again associated with the high turbulence with the 3D-printed stirrer devices. The reaction using powdered catalysts and a conventional stirrer (gray line) displayed a significantly lower rate of the reaction when compared with the catalyst-impregnated stirrer device and the longest reaction time (excluding controls) with a comparatively lower yield of 84% in 65 min. The reaction carried out with the impregnated device was also cleaner when analyzed by LCMS when compared to its solution-based congener (Supporting Information).

**Figure 9:**
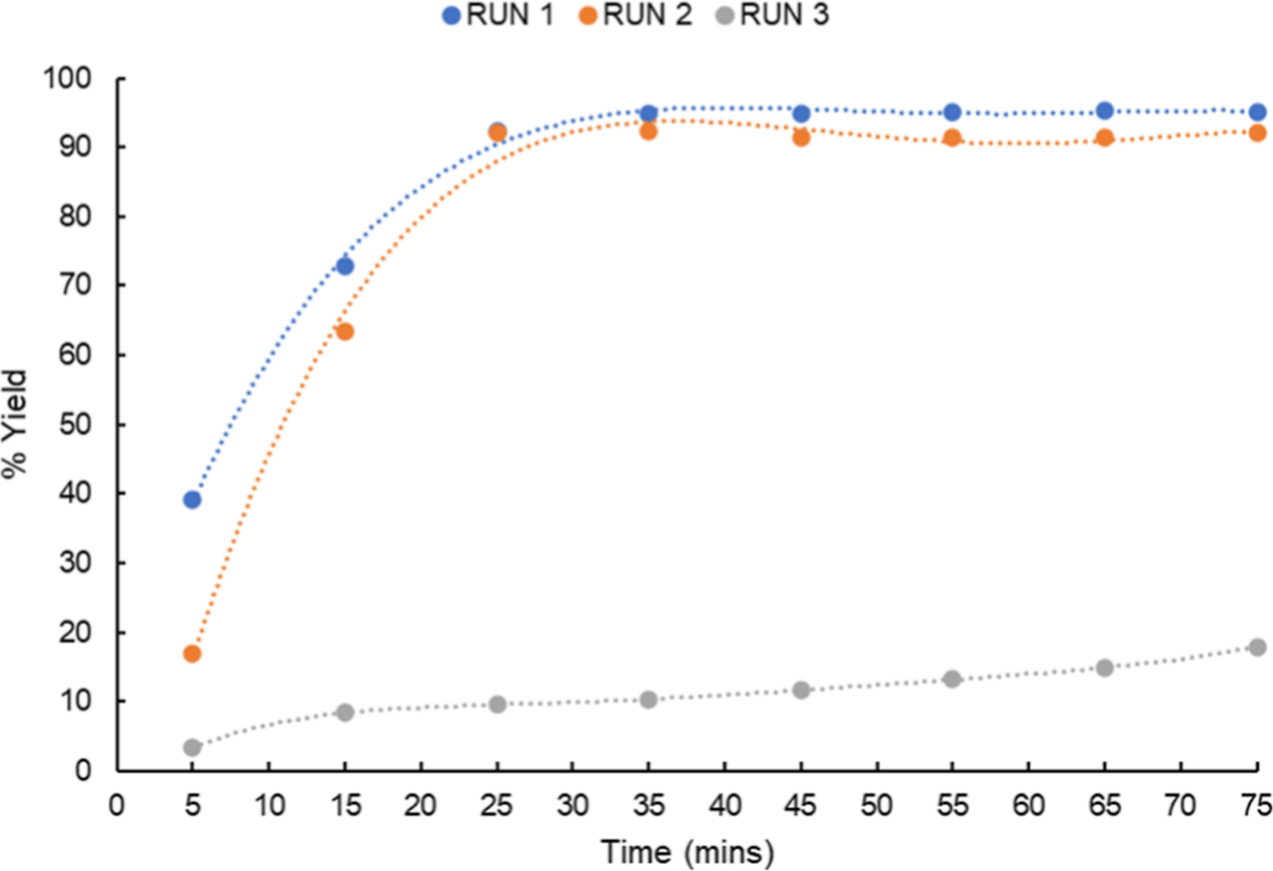
Reusability and reaction profile of the YCl_3_·6H_2_O 3D-printed devices.

A reusability study of the YCl_3_·6H_2_O-impregnated stirrer devices was carried out, where the catalytic device was washed in the reaction solvent (EtOH), dried, and used in the same reaction. The reusability for the first two repeats gave the product in good yields with 95 and 92%, respectively, and similar reaction profiles, but a profound difference in both the reaction rate and final yield was encountered during the third repeat. At the end of the 75 min reaction, a yield of only 18% was recorded, implying that the catalytic device/catalyst on the surface may have undergone degradation on exposure to reactants (Figure 9).

To confirm that the change in the reaction rate was due to the degradation/poisoning of the catalyst and was not due to simple leaching, we investigated catalyst loss using ICP–MS (Table 7).

**Table 7:** ICP–MS Study of Yttrium Leaching in the Reaction; (A) No Catalyst + Blank 3D-Printed Stirrer Device, (B) Powdered Catalyst + Conventional Magnetic Stirrer, (C) Powdered Catalyst + Blank 3D-Printed Stirrer Device, and (D) YCl_3_.6H_2_O-Impregnated 3D-Printed Stirrer Device

	mass of Y metal used in the reaction (mg)	mass of Y metal detected through ICP–MS (mg)	YCl_3_·6H_2_O leached (%)
A	0.00	0.00	0
B	2.67 (in the stirrer device)	0.00560	0.21

From the results obtained, it can be seen that as with the scandium-impregnated devices, there is very little loss of yttrium into the reaction medium. As such, it appears that the loss of activity in the third run is probably due to the slow poisoning of the embedded catalyst on the surface of the device by the reactants. Having demonstrated the utility of the yttrium-impregnated stirrers, a library of benzothiazole compounds were synthesized in excellent yields (Table 8). A range of thiazole and aldehyde derivatives were chosen to investigate the level of tolerance of the stirrer devices to different functional groups.

**Table 8:**
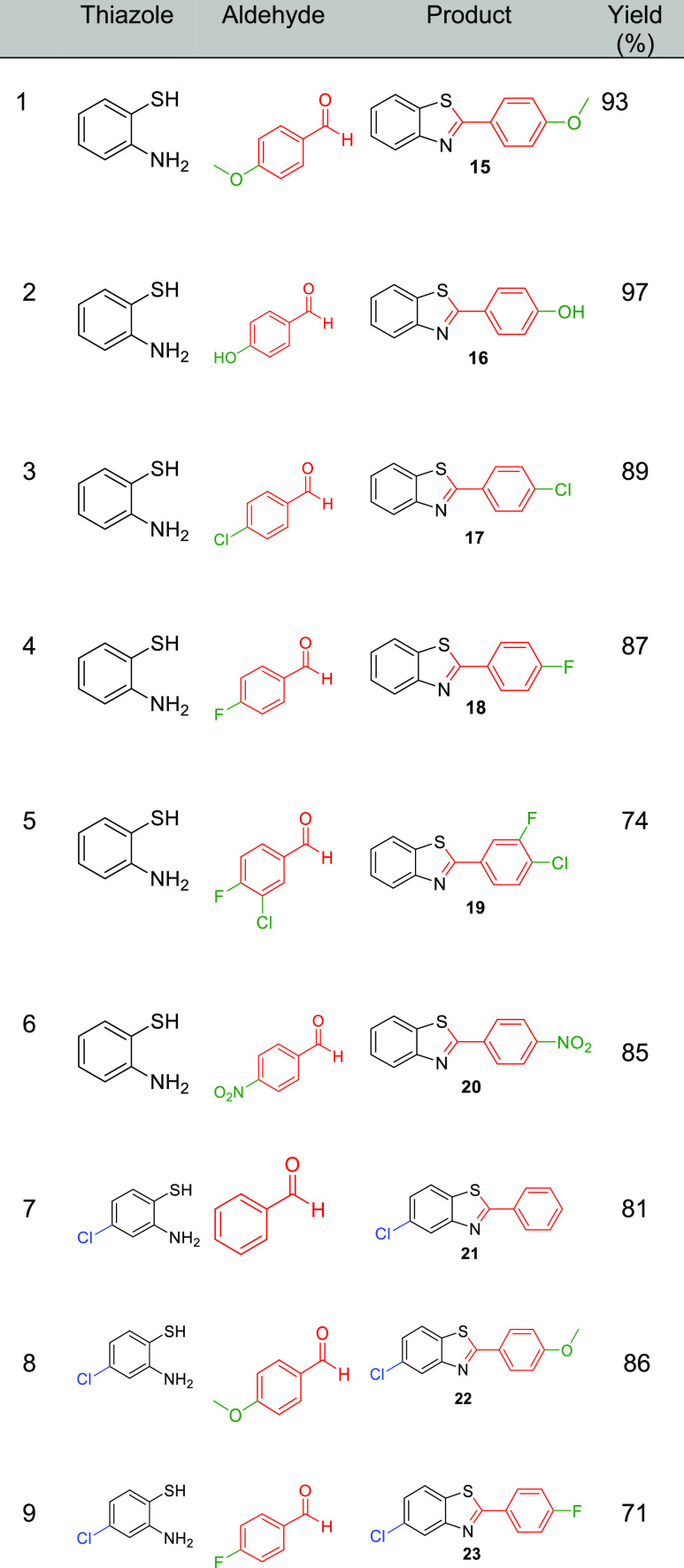
Library of Benzothiazole Compounds Using Derivatives of Thiazoles and Aldehydes

All reactions gave good yields of the substituted benzothiazoles and avoided extensive purification due to the clean nature of the reaction via catalysis from the YCl_3_-embedded 3D-printed devices, clearly highlighting the utility of the LA-impregnated devices.

## Conclusions

In summary, we have demonstrated the significance of 3D printing in chemical synthesis to aid batch reactions through the development of novel 3D-printed stirrer devices that contain LAs and demonstrated their clear advantages over normal batch catalysis. The preliminary investigations into the use of these 3D-printed stirrer devices to optimize both reaction efficiency and reaction simplicity have shown that the efficient stirring of the devices allows for a greater interaction of the reactants in comparison to the traditional synthetic route involving powdered catalyst and conventional stirrer. The reactions of various benzene-1,2-diamines and *o*-aminothiophenols with various benzaldehydes in the presence of a range of LA catalysts and solvents have been carried out and optimized reaction condition have been developed. The use of such devices omits the need for the weighing out of powdered catalysts and simplifies the work up procedure, thus saving time. The ability to reuse the stirrer devices has also been successfully demonstrated, and further investigations as to the exact nature of the catalyst and investigation of the ranges of catalysts that can be impregnated into the stirrer devices will be reported in due course.

## Data Availability

The data underlying this study are available in the published article and its online Supporting Information.

## Supporting Information Available

Details of the production of the stirrer devices, their requisite printing, surface analysis, use, and full experimental details (PDF)
