# Supplementary material for: Rapid Lewis Acid Screening and Reaction Optimization Using 3D-Printed Catalyst-Impregnated Stirrer Devices in the Synthesis of Heterocycles
Source: J Org Chem. 2023 Nov 27;88(24):16845–53. doi: 10.1021/acs.joc.3c01601 (PMC10729026; doi:10.1021/acs.joc.3c01601)
Supplement: Supplementary file 1 — jo3c01601_si_001.pdf [file jo3c01601_si_001.pdf]

# Supporting Information

# Rapid Lewis Acid Screening and Reaction Optimization using 3D Printed Catalyst Impregnated Stirrer Devices in the Synthesis of Heterocycles

Rumintha Thavarajah,[a] Matthew R. Penny,[a] Ryo Torii[b] and Stephen T. Hilton[a]*

[a] UCL School of Pharmacy
University College London
29-39 Brunswick Square, London, WC1N 1AX, United Kingdom
E-mail: s.hilton@ucl.ac.uk

[a] Department of Mechanical Engineering, UCL, Torrington
Place, London WC1E 7JE

Supplementary Information

S1.0. General Experimental & Analysis ........ 3
S2.0. 3D Printing of Lewis Acid catalysts Impregnated Stirrer Devices ........ 4
S2.1. Design of 3D Printed Stirrer ........ 4
S2.2. Resin Formulation ........ 6
S2.2.1. Blank 3D Printed Stirrer Device ........ 6
S2.2.2. Scandium(III) trifluoromethanesulfonate Impregnated 3D Printed Stirrer Devices ........ 6
S2.2.3. Ytterbium(III) trifluoromethanesulfonate Impregnated 3D Printed Stirrer Devices ........ 7
S2.2.4. Indium(III) trifluoromethanesulfonate Impregnated 3D Printed Stirrer Devices ........ 7
S2.2.5. Zinc(II) trifluoromethanesulfonate Impregnated 3D Printed Stirrer Devices 7
S2.2.6. Copper(I) trifluoromethanesulfonate Impregnated 3D Printed Stirrer Devices ........ 8
S2.2.7. Yttrium(III) trifluoromethanesulfonate Impregnated 3D Printed Stirrer Devices ........ 8
S2.2.8. Yttrium chloride hexahydrate Impregnated 3D Printed Stirrer Devices ........ 8
S2.3. Print Settings ........ 9
S2.4. Weight & Amount of Catalyst in Each 3D Printed Stirrer Device ........ 12
S3.0. Vortex Capabilities of 3D Printed Stirrer Devices ........ 13
S3.1. Computational Modelling of the Fluid dynamic evaluation of the 3D-printed stirrer ........ 14
S4.0. Reactions of Lewis Acid Catalysts Impregnated 3D Printed Stirrer Devices ........ 17
S5.0. General Procedures: Benzimidazole Derivatives ........ 23
S6.0. Reactions of Yttrium Chloride Hexahydrate Impregnated Stirrer Devices ........ 30
S7.0. General Procedures: Benzothiazole Derivatives ........ 36
S8.0. NMR Spectra: Benzimidazole Derivatives ........ 41
S6.0. NMR Spectra: Benzothiazole Derivatives ........ 54
S9.0. References ........ 64

## S1.0. General Experimental & Analysis

All reactions were carried out under an atmosphere of nitrogen and all glassware was pre-dried in an oven (110 °C) and cooled under nitrogen prior to use. Stirring was by internal Lewis Acid impregnated 3D printed magnetic follower unless otherwise stated. Reagents and solvents were purchased from Sigma-Aldrich, Merck, Fluka or VWR and used without further purification. Analytical TLC was carried out on Merck silica gel 60 $F_{254}$ pre-coated plastic plates. Short wave UV (245 nm) or $KMnO_4$ were used to visualize components. $^1H$ and $^{13}C$ NMR data were recorded on a Bruker AV400, Bruker AV500 and AV600 spectrometers. Spectra were recorded in deuterochloroform and referenced to residual $CHCl_3$ ($^1H$, 7.26 ppm; $^{13}C$, 77.16 ppm). $^1H$, and $^{13}C$ spectral data were visualized and processed using MestReNova software. Chemical shifts were expressed in ppm (δ) relative to the standard and coupling constants (J) in Hz. High resolution mass spectra were recorded by the National Mass Spectrometry Facility at Swansea University on a LTQ Orbitrap XL utilizing nanospray ionization (NSI) or Xevo G2-S Atmospheric Solids Analysis Probe (ASAP). Infrared spectra were recorded on a Bruker Alpha IR spectrophotometer. Melting points were determined using open glass capillaries on a Stuart Scientific SMP3 apparatus and are uncorrected.

## S2.0. 3D Printing of Lewis Acid catalysts Impregnated Stirrer Devices

### S2.1. Design of 3D Printed Stirrer

A 3D design of the stirrer devices to be printed using the SLA printer was created using the free online CAD software - Tinkercad.[1] The device was designed so that it could be used in both a Radleys carousel reactor or in a round bottomed flask (RBF).[2] The design consisted of a circular device with a dimension of 16.20 mm by 16.20 mm and with a height of 8 mm. The design also contains a central cavity, which is a rectangular hole in the central part of the device to allow the suitable and secure fit of a commercially available conventional magnetic stirrer bar with a dimension of 13 mm by 8 mm. The design also contains six vertical cylindrical holes; three on either side of the central cavity. An additional three cylindrical holes have been added into the central cavity, followed by eight rectangular holes around the surface of the design to allow sufficient mixing and the flow of the solution during a chemical reaction (Supplementary Figures 1 & 2).

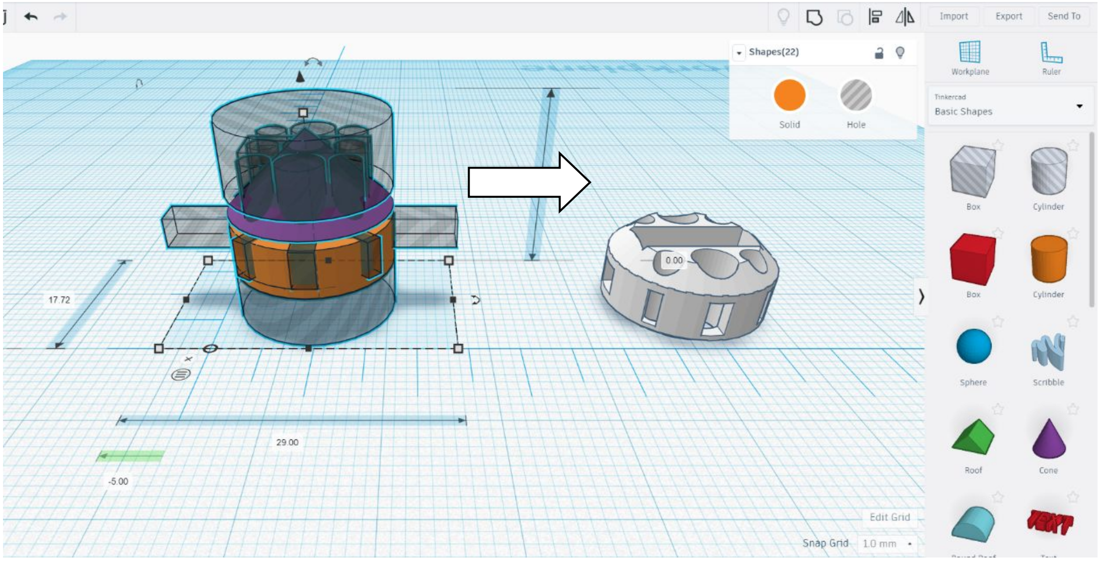


**Supplementary Figure 1**: The process of designing the third generation 3D printed stirrer device.

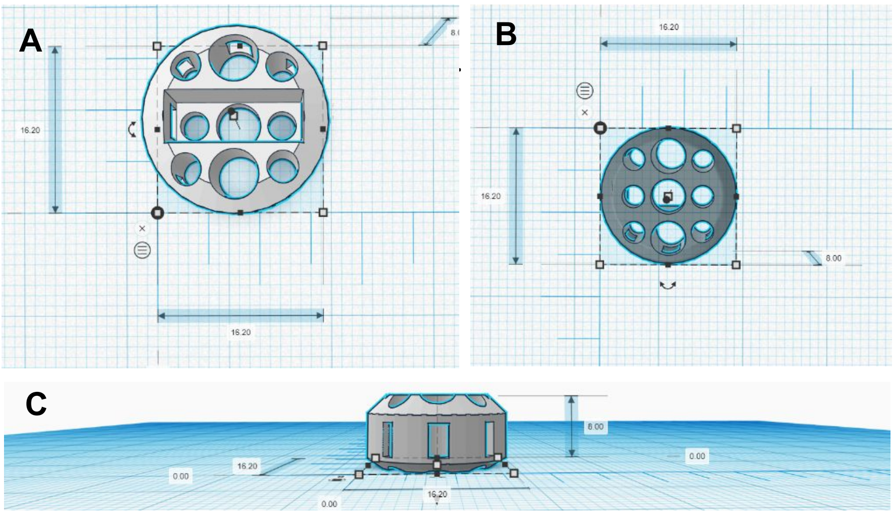


**Supplementary Figure 2**: STL drawing of 3rd generation 3D printed stirrer device design and its corresponding dimensions. A. Upper view B. Lower View C. Peripheral view

### S2.2. Resin Formulation

#### S2.2.1. Blank 3D Printed Stirrer Device

Poly(ethylene glycol) diacrylate $M_n$ 250 (33.3 g) was weighed into a glass bottle (100 mL). The glass bottle was covered in foil, followed by the addition of diphenyl(2,4,6-trimethylbenzoyl)phosphine oxide (0.5 g, 1.47% *(w/w)*). The mixture was stirred continuously at 1000 RPM for 30 minutes at room temperature. The stirrer devices were then printed using a Form 1+ 3D Printer (preform software), using the print settings described below. Post print: the printed stirrer devices were washed in IPA and cured in the Formlabs curing oven at 45 °C for 30 minutes.[3,4]

**Caution!**

The use of acrylates can cause skin irritation. Appropriate PPE is to be used at all times when handling these.

#### S2.2.2. Scandium(III) trifluoromethanesulfonate Impregnated 3D Printed Stirrer Devices

Poly(ethylene glycol) diacrylate $M_n$ 250 (33.3 g) was weighed into a glass bottle (100 mL). The glass bottle was covered in foil and followed by the addition of diphenyl(2,4,6-trimethylbenzoyl)phosphine oxide (0.5 g, 1.47% *(w/w)*) and scandium(**III**) trifluoromethanesulfonate (0.845 g, 2.5% (*w/w*)). The mixture was stirred continuously at 1000 RPM for 2 hours at room temperature. The stirrer devices were then printed using a Form 1+ 3D Printer (preform software), using the print settings described below. Post print: the printed stirrer devices were washed in IPA and cured in the Formlabs curing oven at 45 °C for 30 minutes.

### S2.2.3. Ytterbium(III) trifluoromethanesulfonate Impregnated 3D Printed Stirrer Devices

Poly(ethylene glycol) diacrylate $M_n$ 250 (33.3 g) was weighed into a glass bottle (100 mL). The glass bottle was covered in foil and followed by the addition of diphenyl(2,4,6-trimethylbenzoyl)phosphine oxide (0.5 g, 1.47% *(w/w)*) and ytterbium(III) trifluoromethanesulfonate (0.845 g, 2.5% (*w/w*)) was used as the catalyst. The mixture was stirred continuously for 1 hour at room temperature. The stirrer devices were then printed using a Form 1+ 3D Printer (preform software), using the print settings described below. Post print: the printed stirrer devices were washed in IPA and cured in the Formlabs curing oven at 45 °C for 30 minutes.

### S2.2.4. Indium(III) trifluoromethanesulfonate Impregnated 3D Printed Stirrer Devices

Poly(ethylene glycol) diacrylate $M_n$ 250 (33.3 g) was weighed into a glass bottle (100 mL). The glass bottle was covered in foil and followed by the addition of diphenyl(2,4,6-trimethylbenzoyl)phosphine oxide (0.5 g, 1.47% *(w/w)*) and indium(III) trifluoromethanesulfonate (0.845 g, 2.5% (*w/w*)) was used as the catalyst. The mixture was stirred continuously for 1 hour at room temperature. The stirrer devices were then printed using a Form 1+ 3D Printer (preform software), using the print settings described below. Post print: the printed stirrer devices were washed in IPA and cured in the Formlabs curing oven at 45 °C for 30 minutes.

### S2.2.5. Zinc(II) trifluoromethanesulfonate Impregnated 3D Printed Stirrer Devices

Poly(ethylene glycol) diacrylate $M_n$ 250 (33.3 g) was weighed into a glass bottle (100 mL). The glass bottle was covered in foil and followed by the addition of diphenyl(2,4,6-trimethylbenzoyl)phosphine oxide (0.5 g, 1.47% *(w/w)*) and zinc(II) trifluoromethanesulfonate (0.845 g, 2.5% (*w/w*)) was used as the catalyst. The mixture was stirred continuously for 1 hour at room temperature. The stirrer devices were then printed using a Form 1+ 3D Printer (preform software), using the print settings described below. Post print: the printed stirrer devices were washed in and cured in the Formlabs curing oven at 45 °C for 30 minutes.

### S2.2.6. Copper(I) trifluoromethanesulfonate Impregnated 3D Printed Stirrer Devices

Poly(ethylene glycol) diacrylate $M_n$ 250 (33.3 g) was weighed into a glass bottle (100 mL). The glass bottle was covered in foil and followed by the addition of diphenyl(2,4,6-trimethylbenzoyl)phosphine oxide (0.5 g, 1.47% *(w/w)*) and copper(I) trifluoromethanesulfonate (0.845 g, 2.5% (*w/w*)) was used as the catalyst. The mixture was stirred continuously for 1 hour at room temperature. The stirrer devices were then printed using a Form 1+ 3D Printer (preform software), using the print settings described below. Post print: the printed stirrer devices were washed in IPA and cured in the Formlabs curing oven at 45 °C for 30 minutes.

### S2.2.7. Yttrium(III) trifluoromethanesulfonate Impregnated 3D Printed Stirrer Devices

Poly(ethylene glycol) diacrylate $M_n$ 250 (33.3 g) was weighed into a glass bottle (100 mL). The glass bottle was covered in foil and followed by the addition of diphenyl(2,4,6-trimethylbenzoyl)phosphine oxide (0.5 g, 1.47% *(w/w)*) and yttrium(III) trifluoromethanesulfonate (0.845 g, 2.5% (*w/w*)) was used as the catalyst. The mixture was stirred continuously for 4 hours at room temperature. The stirrer devices were then printed using a Form 1+ 3D Printer (preform software), using the print settings described below. Post print: the printed stirrer devices were washed in IPA and cured in the Formlabs curing oven at 45 °C for 30 minutes.

### S2.2.8. Yttrium chloride hexahydrate Impregnated 3D Printed Stirrer Devices

Poly(ethylene glycol) diacrylate $M_n$ 250 (33.3 g) was weighed into a glass bottle (100 mL). The glass bottle was covered in aluminium foil and followed by the addition of diphenyl(2,4,6-trimethylbenzoyl)phosphine oxide (0.5 g, 1.47% *(w/w)*) and yttrium(III) chloride hexahydrate (0.34 g, 1.0% *(w/w)*) was used as the catalyst. The mixture was stirred continuously for 4 hours at room temperature. The stirrer devices were then printed using a Form 1+ 3D Printer (preform software), using the print settings described below. Post print: the printed stirrer devices were washed in IPA and cured in the Formlabs curing oven at 45 °C for 30 minutes.

### S2.3. Print Settings

Once the designs had been exported as .stl files, they were uploaded to FormLabs PreForm Software (version 2.10.3) before printing. Support structures to aid printing were generated using PreForm Software. The resin formulation containing the Lewis acid catalyst was poured into the tray of a Form1+ 3D Printer.[3,4]

The print settings differ when printing a blank 3D printed stirrer device without catalyst and when incorporating various catalysts into the stirrer devices. After many iterative changes, the accurate and precise print setting required when printing with different types of catalysts were developed. The table below (Supplementary Table 1) illustrates the different print settings required for a range of Lewis acid catalysts screened.

All the stirrer devices were printed horizontally with a base and supports attached onto the stirrer device. The lower the number of supports required, the less resin needed. Some catalysts required many supports when compared to the others: blank, ytterbium triflate ($Yb(OTf)_3$), indium triflate ($In(OTf)_3$), zinc triflate ($Zn(OTf)_2$), copper triflate (CuOTf) and yttrium triflate ($Y(OTf)_3$) required four supports, while scandium triflate ($Sc(OTf)_3$) needed 10. The blank, $Yb(OTf)_3$, $In(OTf)_3$, $Zn(OTf)_3$ and $Y(OTf)_3$ stirrer devices needed to be printed using the clear material setting in the version V1(FLGPCLO1), where the final object had an opaque finish. Both $Sc(OTf)_3$ and CuOTf required a the black resin setting in the version V1(FLGPBK01) where the final object has a translucent finish. If the right print settings were not used, it led to over polymerisation, poor quality of print, poor resolution, severe contraction of polymer (where the magnetic stirrer bar did not fit in the central cavity of the printed device causing the device to break) and the final object was brittle/ flaky and as a result, additional time was needed to cure. The time taken and volume of resin needed to print multiple 3D stirrer devices in a single print is described below (Supplementary Table 2).

| Catalyst | Material | Version | No. of Supports | Support Point Size (mm) | No. of Layers to Print 1 Object without Supports | No. of Layers to Print 1 Object with Supports |
|---|---|---|---|---|---|---|
| Blank | Clear | V1 (FLGPCL01) | 4 | 0.60 | 80 | 150 |
| $Sc(OTf)_3$ | Black | V1 (FLGPBK01) | 10 | 1.00 | 80 | 120 |
| $Yb(OTf)_3$ | Clear | V1 (FLGPCL01) | 4 | 0.60 | 80 | 150 |
| $In(OTf)_3$ | Clear | V1 (FLGPCL01) | 4 | 0.60 | 80 | 150 |
| $Zn(OTf)_2$ | Clear | V1 (FLGPCL01) | 4 | 0.60 | 80 | 150 |
| CuOTf | Black | V1 (FLGPBK01) | 4 | 0.60 | 80 | 150 |
| $Y(OTf)_3$ | Clear | V1 (FLGPCL01) | 4 | 0.60 | 80 | 150 |

**Supplementary Table 1**: The required print settings for different Lewis acid catalysts.

| | Volume of Resin used (mL) | | Time taken to complete print (mins) | |
|---|---|---|---|---|
| The No. of 3D Printed Stirrer Devices to be Printed with Supports | Blank, $Yb(OTf)_3$, $In(OTf)_3$, $Zn(OTf)_2$, CuOTf, $Y(OTf)_3$ | $Sc(OTf)_3$ | Blank, $Yb(OTf)_3$, $In(OTf)_3$, $Zn(OTf)_2$, CuOTf, $Y(OTf)_3$ | $Sc(OTf)_3$ |
| 1 | 1.07 | 1.15 | 26 | 23 |
| 2 | 2.15 | 2.30 | 28 | 26 |
| 3 | 3.22 | 3.45 | 30 | 30 |
| 4 | 4.29 | 4.60 | 33 | 33 |
| 5 | 5.37 | 5.75 | 35 | 36 |
| 6 | 6.44 | 6.90 | 37 | 40 |
| 7 | 7.51 | 8.05 | 39 | 43 |
| 8 | 8.59 | 9.20 | 42 | 46 |
| 9 | 9.66 | 10.36 | 44 | 50 |
| 10 | 10.73 | 11.51 | 46 | 53 |

**Supplementary Table 2**: The time taken, and volume of resin needed to print multiple 3D stirrer devices in a single print.

The arrangement of the stirrer devices to be printed varied from catalyst to catalyst and the setup is illustrated below (Supplementary Figure 3). The print had to be monitored from time to time to make sure the devices adhere to the build platform, if not the base thickness had to be adjusted and the parts checked to ensure it didn't detach from its supports during the print - if not the point size had to adjusted.

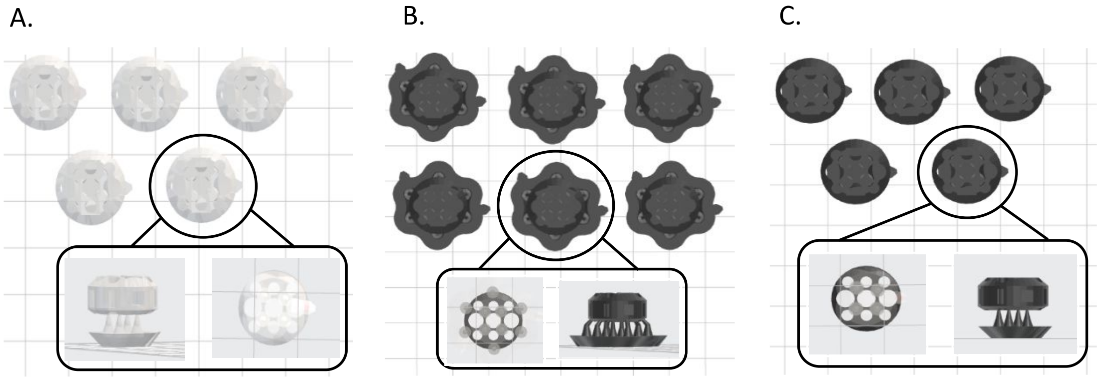


**Supplementary Figure 3:** The orientation of printing the stirrer devices with different Lewis acid catalysts using the SLA printer. A: blank, $Yb(OTf)_3$, $In(OTf)_3$, $Zn(OTf)_2$,$Y(OTf)_3$ stirrer devices B: $Sc(OTf)_3$ stirrer devices C: CuOTf stirrer devices.

After the print was complete, the object was removed from the build plate and was washed in isopropanol (IPA) for about 10 seconds. The stirrer devices were cured in the Formlabs curing oven for 30 minutes at 45 °C, followed by removal of the supports from the stirrer devices and the insertion of the magnetic stirrer bar into the central cavity of the 3D printed stirrer device (Supplementary Table 3).

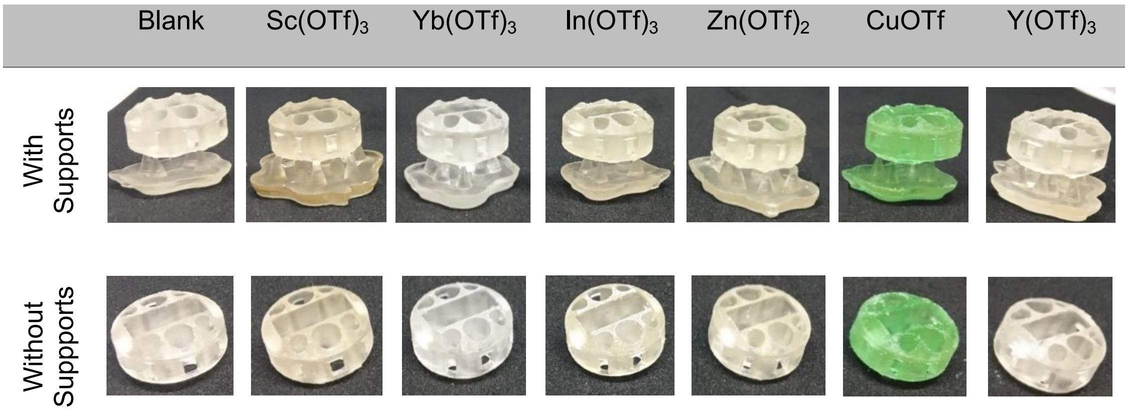


**Supplementary Table 3:** The appearance of the catalyst impregnated 3D printed stirrer devices after curing.

## S2.4. Weight & Amount of Catalyst in Each 3D Printed Stirrer Device

The weight and the amount of catalyst in each 3D printed stirrer device has been calculated using 10 3D printed stirrer devices as the sample size. There was a minor change in mass of each stirrer device, therefore the catalyst loading variance of the mean has been calculated to account for the change in mass. There is a slight variation in the standard error of the mean (Supplementary Table 4) of less than 0.5% for all catalysts examined. The change in mass could have occurred when removing the supports from the 3D printed stirrer devices where too much or too little of the supports could have been removed. However, this negligible difference suggests good uniformity of the 3D printed stirrer devices, which could mean the catalyst is distributed evenly in the devices.

| | Average Weight Range (g) | Catalyst Loading Range (g) | Catalyst Loading Variance (%) |
|---|---|---|---|
| Blank | 0.8491 g ± 1.37 mg | _ | _ |
| $Sc(OTf)_3$ | 0.8130 g ± 2.36 mg | 20.3 mg ± 0.060 mg | 0.29 |
| $Yb(Otf)_3$ | 0.9430 g ± 2.82 mg | 23.6 mg ± 0.071 mg | 0.30 |
| $In(Otf)_3$ | 0.8504 g ± 2.51 mg | 21.3 mg ± 0.063 mg | 0.30 |
| $Zn(Otf)_2$ | 0.9100 g ± 3.74 mg | 22.8 mg ± 0.094 mg | 0.41 |
| CuOTf | 0.7740 g ± 3.28 mg | 19.3 mg ± 0.082 mg | 0.42 |
| $Y(Otf)_3$ | 0.8460 g ± 3.48 mg | 21.2 mg ± 0.087 mg | 0.41 |

**Supplementary Table 4:** The weight and the amount of catalyst in each 3D printed stirrer device with corresponding catalyst loading variance.

## S3.0. Vortex Capabilities of 3D Printed Stirrer Devices

A carousel vial was placed in a 3D printed vial holder with a ruler set up. This was to ensure the vial was kept in the middle of the stirrer hot plate and there could be no variation in the in the height of the vial each time the test was repeated.

Deionised water (5 mL) was placed in the carousel vial and a conventional stirrer/blank 3D printed stirrer device was added and the vial was placed in the 3D printed vial holder containing a ruler. This was placed on a stirrer hot plate and the initial height was measured from the bottom of the carousel vial to the top of the solvent (deionised water) level. While stirring at each RPM, the final height was measured from the bottom of the carousel vial to the top of the solvent (deionised) level. The difference in final height and the initial height was concluded as the vortex height. This above procedure was repeated for the rest of the RPM values and was repeated 2 times to obtain results in triplicate (Supplementary Figure 4).

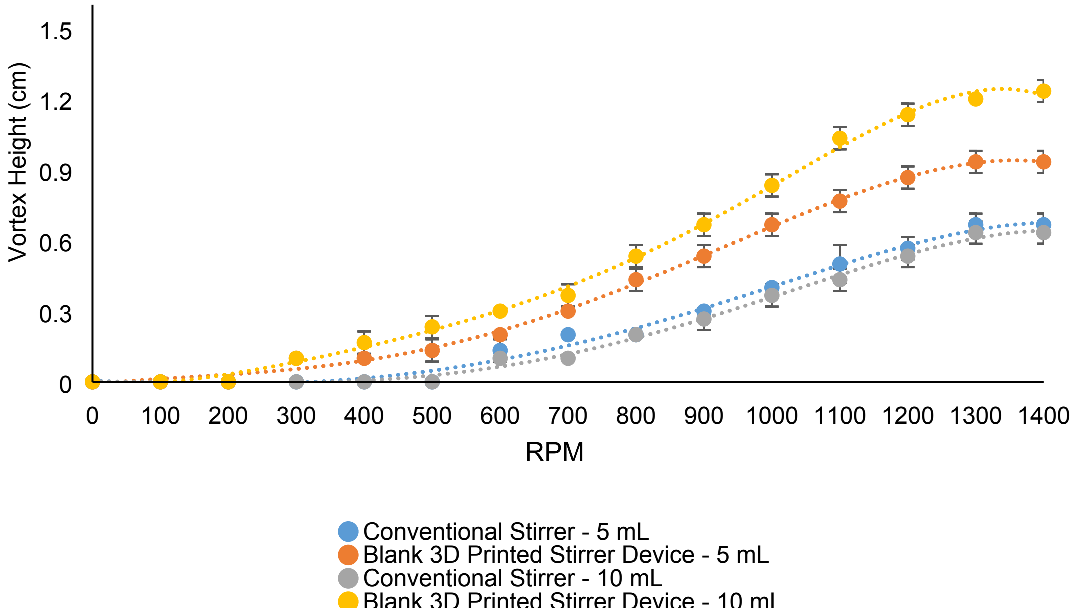


**Supplementary Figure 4:** Vortex Heights achieved using the 3D Printed stirrer devices

## S3.1. Computational Modelling of the Fluid dynamic evaluation of the 3D-printed stirrer

CFD modelling was carried out in Ansys Fluent software version 19.2 (Canonsburg PA, USA). Air temperature was assumed to be 25 ºC with no surface tension considered at the interface between the air and the solvent (ethanol). Solvent density was set at 0.7893 g/cm3 and viscosity at 1.074 mPa s. In order to allow for sufficient analysis of mixing, the stirrer height was set at 12 mm below the solvent/air interface as shown (Supplementary Figure 5).

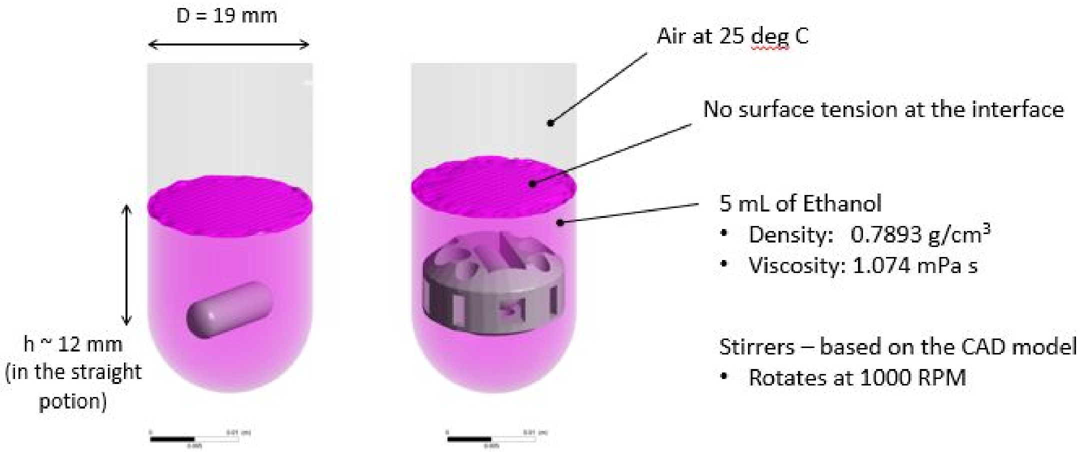


**Supplementary Figure 5:** Vortex Heights achieved using the 3D Printed stirrer devices

Comparison of mixing between a conventional stirrer device and the 3D printed construct was carried out at 1000 RPM and over a range of times as shown below (Supplementary Figure 6).

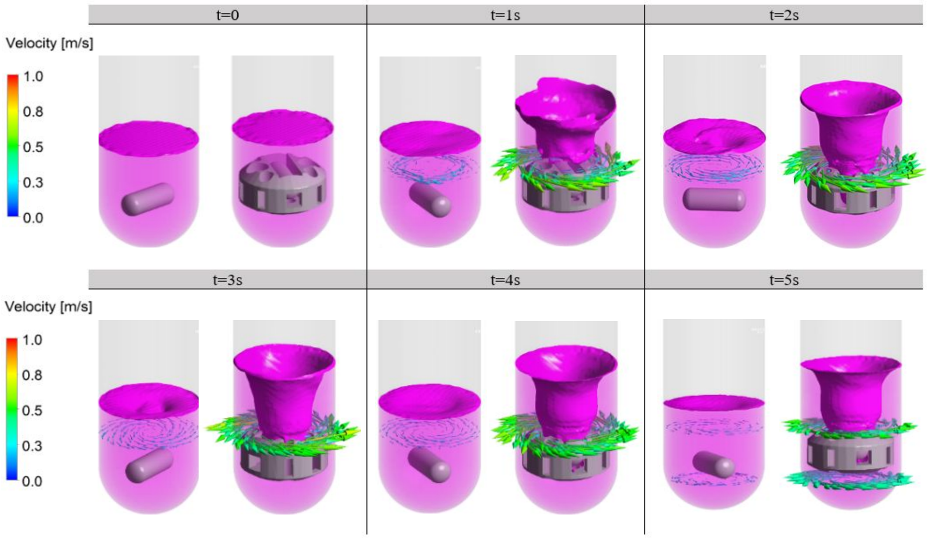


**Supplementary Figure 6:** CFD modelling of vortex formation and mixing at 1000 RPM.

Swirling velocity was calculated, as an average of in-plane velocity magnitude, in the two planes above and below the conventional stirrer and that of the 3D printed device (Supplementary Figure 7).

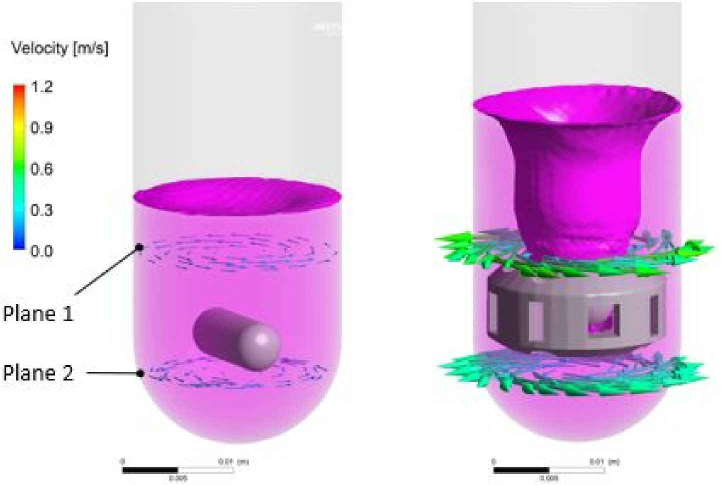


Average 'swirling flow' velocity magnitude in the two planes

| Plane | Standard | 3D printed |
| --- | --- | --- |
| 1 | 0.097 [m/s] | 0.234 [m/s] |
| 2 | 0.278 [m/s] | 0.407 [m/s] |

**Supplementary Figure 7:** CFD modelling of swirling velocity above and below the two types of stirrers analysed.

The results highlight that the 3D printed stirrer is able to induce a greater degree of swirling speed almost instantly after 1 second from start, and continuously providing a higher swirling speed, more than 2-fold, in comparison to the conventional stirrer.

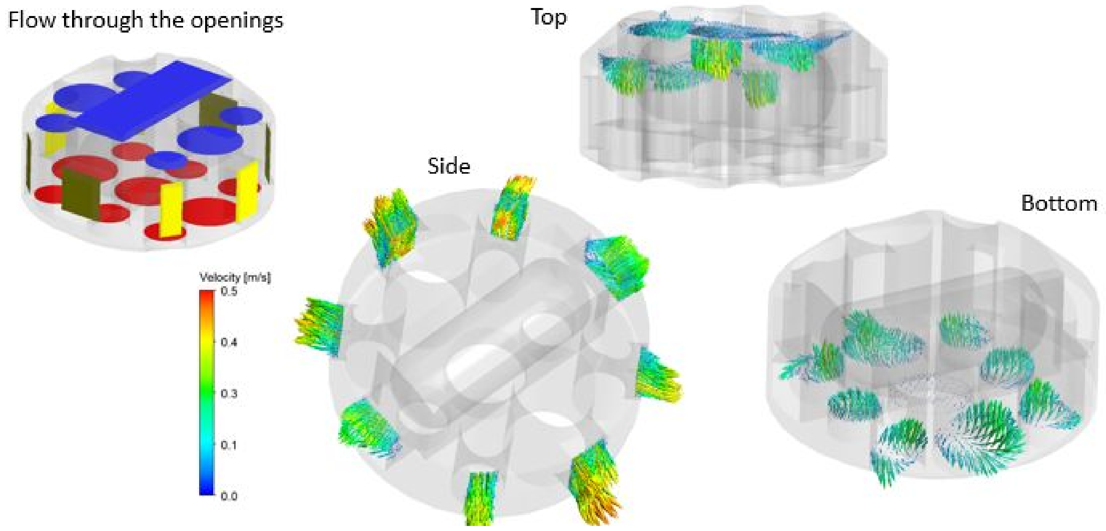


**Supplementary Figure 8:** CFD modelling of fluidic flow through the 3D printed stirrer.

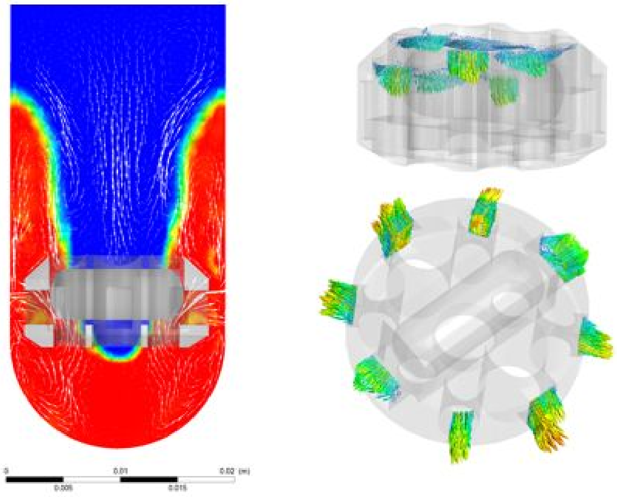

**Supplementary Figure 9:** CFD modelling of mixing and fluidic flow through the sides of the device.

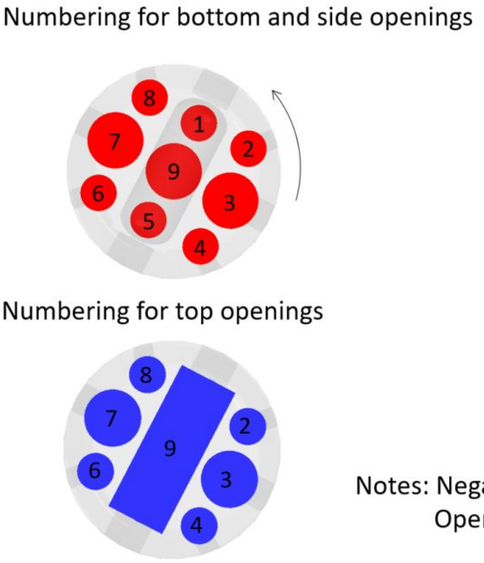


**Quantitative flow** (instantaneous at t=0.4 s from beginning)

**Total flow through the stirrer: 12.5 mL/s**

| Opening | Flow rate [mL/s] | | |
|---|---|---|---|
| | Bottom | Top | Side |
| 1 | -0.69 | -0.75 | 1.18 |
| 2 | -0.66 | – | 1.42 |
| 3 | -1.01 | -1.09 | 2.11 |
| 4 | -0.42 | -1.04 | 1.46 |
| 5 | -0.79 | – | 1.15 |
| 6 | -0.66 | -0.86 | 1.50 |
| 7 | -1.07 | -1.05 | 2.13 |
| 8 | -0.62 | -0.95 | 1.56 |
| 9 | 0.21 | -1.07 | – |

Notes: Negative flow is sucked into, positive flow is going out from the stirrer. Opening 9 (both top and bottom) are mostly occupied by the air.

**Supplementary Figure 10:** CFD modelling of fluidic flow through the 3D printed stirrer device.

Supplementary Figures 8-10 illustrate the flow through the stirrer, which play a significant role to enhance the reaction, the design objective of this novel stirrer. In total, flow of 12.5 mL/s is guided through the internal cavities of the stirrer that is exposed to the surface.

## S4.0. Reactions of Lewis Acid Catalysts Impregnated 3D Printed Stirrer Devices

Firstly, the reaction of benzene-1,2-diamine and benzaldehyde in the presence of various Lewis acid catalysts in ethanol (EtOH) was carried out (Supplementary Scheme 1).

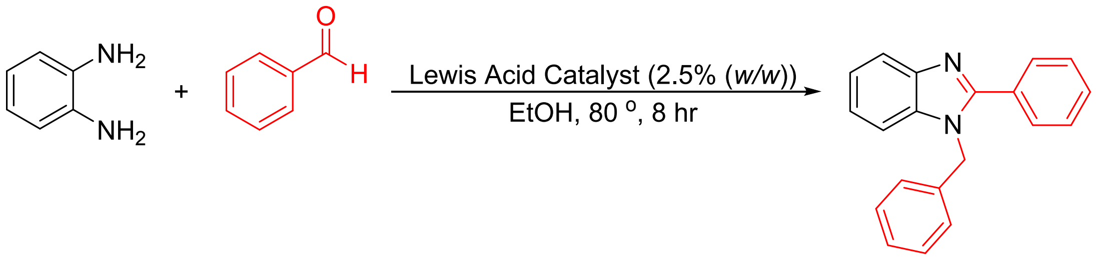


**Supplementary Scheme 1**: Reaction of benzene-1,2-diamine and benzaldehyde in the presence of various Lewis acid catalysts in EtOH.

The control reaction: with no catalysts and a conventional stirrer resulted in a yield of 6%. When the control reaction was repeated using a blank 3D printed stirrer device instead of a conventional stirrer, a yield of 14% was achieved. The increase in yield when using a blank 3D printed stirrer device many have occurred due to the rapid mixing abilities of the 3D printed stirrer devices in comparison to the conventional stirrer (Supplementary Table 5).

All the reactions were carried out in a carousel but the reaction with $Sc(OTf)_3$ was also repeated in a round bottom flask (RBF) using an Asynt DrySyn mantle to further investigate whether similar yields can be achieved using different glassware. The obtained yields show that, when the catalyst impregnated stirrer devices were used both in the carousel and RBF, similar results can be obtained.

There was a visible colour change of the catalyst impregnated stirrer devices after the reaction with benzene-1,2-diamine and benzaldehyde (Supplementary Table 6).

| Isolated Yield (%) | Form of Catalyst | | | | | |
|---|---|---|---|---|---|---|
| | A | | B | | C | |
| $Sc(OTf)_3$ | 61 | 62* | 64 | 65* | 78 | 74* |
| $Yb(OTf)_3$ | 37 | | 54 | | 71 | |
| $In(OTf)_3$ | 49 | | 63 | | 65 | |
| $Zn(OTf)_2$ | 38 | | 53 | | 57 | |
| CuOTf | 25 | | 33 | | 38 | |
| Y(OTf) | 23 | | 25 | | 34 | |

**Supplementary Table 5**: A = Conventional stirrer + powdered catalyst (0.1 mmol, 0.049 g) B = Blank 3D printed stirrer device + powdered catalyst (0.1 mmol, 0.049 g) C = Lewis acid catalyst impregnated 3D printed stirrer device. * = RBF

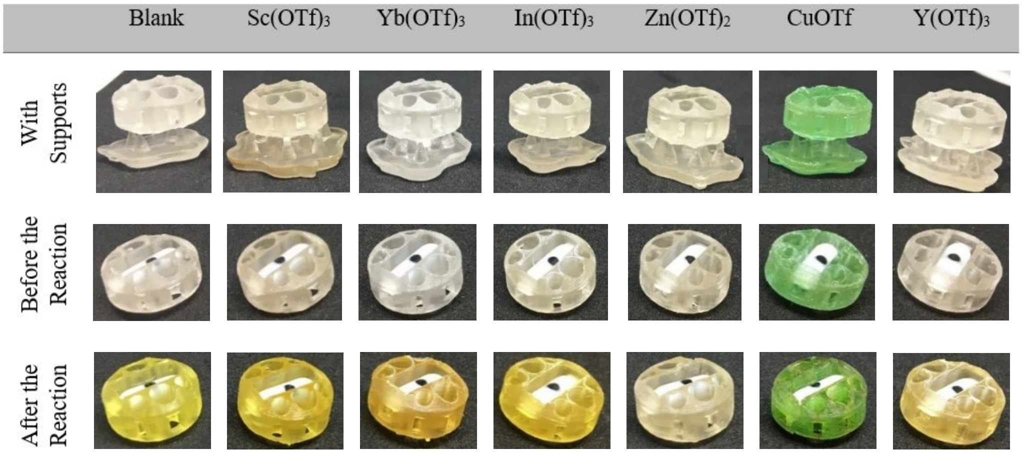


**Supplementary Table 6:** Appearance of the catalyst impregnated stirrer devices before and after the reaction.

Having established that $Sc(OTf)_3$ catalysed the reaction of benzene-1,2-diamine and benzaldehyde most efficiently, a solvent screening test was next carried out. Acetonitrile was found to be the best solvent (Supplementary Table 7), which gave the highest yield and the shortest reaction times, out of all the other solvents screened.

| Solvent | Temperature of Reaction (° C) | Time of Reflux (hr) | Isolated Yield (%) |
|---|---|---|---|
| MeCN | 80 | 2 | 87 |
| MeCN | 80 | 4 | 84 |
| MeCN | 80 | 6 | 79 |
| MeCN (dry) | 80 | 6 | 71 |
| MeCN + water ($H_2O$) (4:1) | 80 | 6 | 52 |
| EtOH | 80 | 2 | 51 |
| Ethyl acetate (EtOAc) | 80 | 6 | 55 |
| Methanol (MeOH) (dry) | 80 | 6 | 82 |
| *tert*-butanol | 80 | 6 | 68 |
| IPA | 80 | 6 | 65 |
| $H_2O$ | 100 | 6 | 42 |
| Tetrahydrofuran (THF) (dry) | 80 | 6 | 27 |
| Toluene | 100 | 6 | 63 |

**Supplementary Table 7**: Screening of polar and non-polar solvents to optimise the reaction conditions.

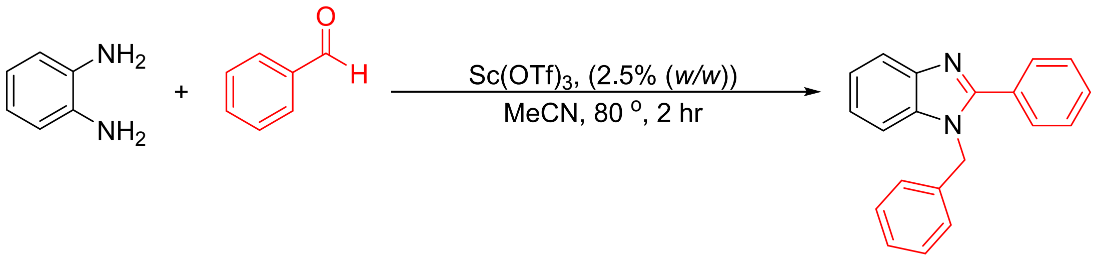


**Supplementary Scheme 2**: Optimised reaction condition for the synthesis of 1-benzyl-2-phenyl-1*H*-benzo[*d*]imidazole using benzene-1,2-diamine, benzaldehyde and $Sc(OTf)_3$ as the catalyst in MeCN.

The LCMS illustrated the reaction purity obtained when using the $Sc(OTf)_3$ impregnated stirrer versus a conventional batch based reaction with powdered $Sc(OTf)_3$. Although a work-up was not carried out for the reaction synthesised using the catalytic device, the LCMS is almost identical to that of the reaction carried out using the traditional method and as such, it did not have any effect on the purity of the final compound. The product peak is at 3.3 minutes and can be clearly seen in both mass spectrums, which are comparable (Supplementary Figure 11).

**A:** Powdered $Sc(OTf)_3$

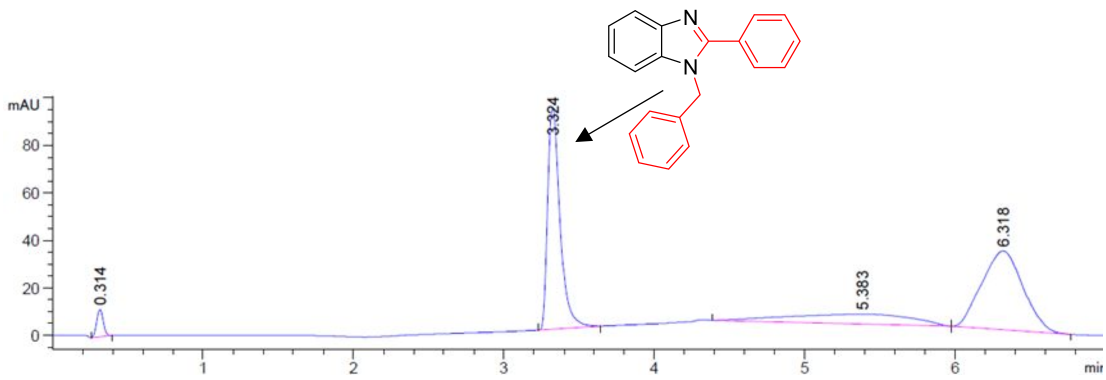


**B:** $Sc(OTf)_3$ Impregnated Stirrer Device

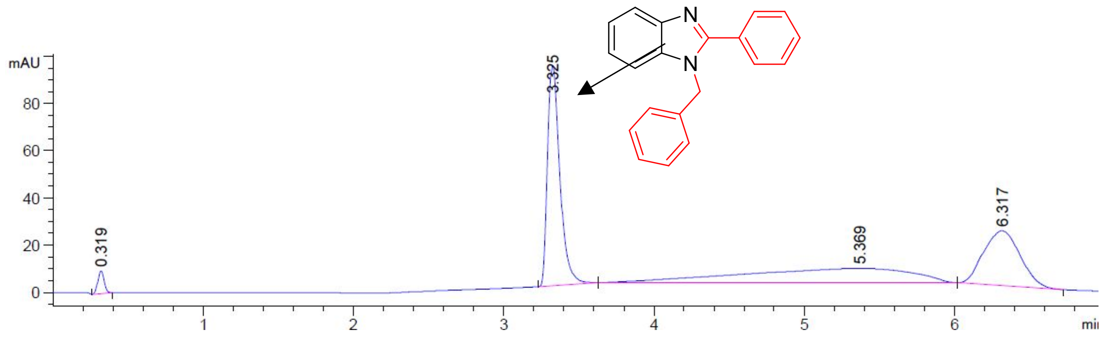


**Supplementary Figure 11:** LCMS comparison of powdered catalyst versus the 3D printed catalyst embedded stirrer device.

Reactions were repeated using the above optimised reaction conditions (Supplementary Scheme 2). According to calculations, there is approximately 20.3 mg of $Sc(OTf)_3$ in each device, so a reaction was also carried out using 20.3 mg of powdered catalyst as a comparison (Supplementary Table 8), which gave the product in 63% yield.

| Form of catalyst | % Isolated Yield |
|---|---|
| No Catalyst + Conventional Stirrer | 0 |
| No Catalyst + Blank 3D Printed Stirrer Device | 0 |
| Powdered Catalyst (0.049 g, 0.1 mmol) + Conventional Stirrer | 68 |
| Powdered catalyst (0.0203 g) + conventional Stirrer | 63 |
| Powdered Catalyst (0.049 g, 0.1 mmol) + Blank 3D Printed Stirrer Device | 79 |
| Powdered catalyst (0.0203 mg) + Blank 3D printed stirrer device | 76 |
| Catalyst Impregnated Stirrer Device | 87 |

**Supplementary Table 8**: Isolated yields obtained from different forms of catalysts.

A reusability test was carried out using the same $Sc(OTf)_3$ impregnated 3D printed stirrer device. The pictures show the change in the stirrer device after each use (Supplementary Table 9). There were visible cracks on the surface of the device, which did not retain its morphology after the second use. We attributed the loss of structural integrity due to mechanical stress, which led to a deterioration of the device over time. The yields of the reaction are consistent for the first two repeats and the yields drops from the third repeat.

| | RUN 1 | RUN 2 | RUN 3 | RUN 4 | RUN 5 |
|---|---|---|---|---|---|
| Appearance of 3D Printed Stirrer Device | 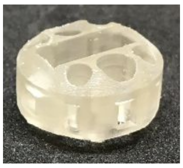 | 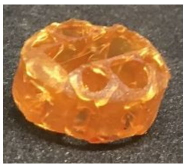 | 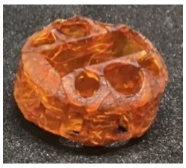 | 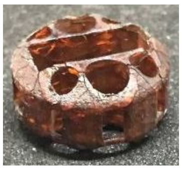 | 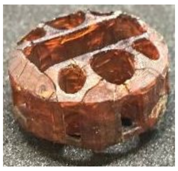 |
| Isolated yield (%) | 86 | 83 | 66 | 58 | 32 |

**Supplementary Table 9**: Appearance of the $Sc(OTf)_3$ impregnated stirrer devices before each use with their corresponding yields.

## S5.0. General Procedures: Benzimidazole Derivatives

### S5.1. General Procedure A: 1-Benzyl-2-phenyl-1*H*-benzo[*d*]imidazole (01)[5]

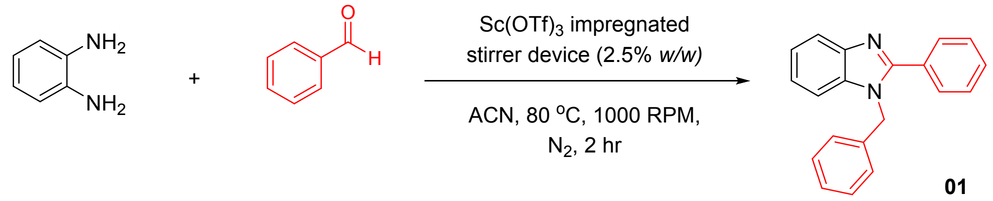


Chemical Formula: $C_6H_8N_2$
Molecular Weight: 108.1440

Chemical Formula: $C_7H_6O$
Molecular Weight: 106.1240

Chemical Formula: $C_{20}H_{16}N_2$
Molecular Weight: 284.3620

Acetonitrile (5 mL) was added to a scandium (III) trifluoromethanesulfonate impregnated 3D printed stirrer device in a Radleys carousel tube on a Radleys carousel reactor (25 mL) under nitrogen, followed by addition of benzene-1,2-diamine (0.6 mmol, 0.065 g). The resulting mixture was heated to 80 °C on a Radleys carousel reactor, whereupon benzaldehyde (1 mmol, 0.106 g, 0.102 mL) was added and heating was continued at 80 °C whilst stirring at 1000 RPM for 2 hours. After completion of the reaction, the mixture was cooled to room temperature and the scandium (III) trifluoromethanesulfonate impregnated 3D printed stirrer device was washed with acetonitrile (1-2 mL). The combined mixture was concentrated under reduced pressure, adsorbed onto silica using dichloromethane and loaded onto a Biotage SNAP cartridge as a fine powder and purified by automatic column chromatography (3:1 Hexane : EtOAc; SNAP KP-Sil 25 g column) to give 1-benzyl-2-phenyl-1*H*-benzo[*d*]imidazole **01** (0.12 g, 87%); m.p. 132-134 °C (lit.[5] 131-133 °C); $^1$H NMR (400 MHz, Chloroform-*d*) δ = 7.88 (d, *J*=8.0, 1H), 7.73 – 7.67 (m, 2H), 7.49 – 7.42 (m, 3H), 7.35 – 7.28 (m, 4H), 7.25 – 7.18 (m, 2H), 7.13 – 7.08 (d, *J*=8.0, 2H), 5.46 (s, 2H); $^{13}$C{1H} NMR (101 MHz, Chloroform-*d*) δ = 154.3, 143.4, 136.6, 136.2, 130.3, 130.0, 129.4, 129.2, 128.9, 127.9, 126.1, 123.2, 122.8, 120.2, 110.6, 48.5; $\tilde{\nu}_{max}$ /cm$^{-1}$ (ATR); 3032, 2946, 1603, 1584, 1493, 1471, 1463, 1449, 1438, 1277, 1249; *m/z* (NSI) Found 285.1387 ($[M+H]^+$ $C_{20}H_{17}N_2$ requires 285.1392).

### 1-(4-Methoxybenzyl)-2-(4-methoxyphenyl)-1*H*-benzo[*d*]imidazole (02)[6]

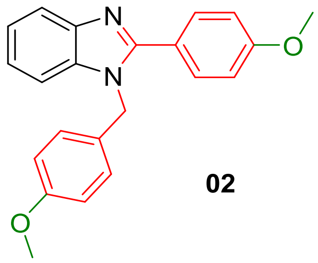

**02**

According to general procedure **A** and purification by automatic column chromatography (3:1 Hexane : EtOAc; SNAP KP-Sil 25 g column), compound **02** was obtained as a colourless solid (0.17 g, 99%); m.p. 129-131 °C (lit.[6] 130-131 °C); $^1$H NMR (400 MHz, Chloroform-*d*) δ = 7.84 (d, *J*=8.0, 1H), 7.75 – 7.34 (m, 2H), 7.29 (ddd, *J*=8.1, 5.3, 3.1, 1H), 7.25 – 7.12 (m, 2H), 7.03 (d, *J*=8.7, 2H), 7.01 – 6.91 (m, 2H), 6.94 – 6.77 (m, 2H), 5.39 (s, 2H), 3.85 (s, 3H), 3.79 (s, 3H); $^{13}$C{1H} NMR (126 MHz, Chloroform-*d*) δ = 161.0, 159.2, 154.3, 143.3, 136.2, 130.8, 127.4, 122.9, 122.6, 122.6, 119.9, 114.6, 114.3, 110.5, 55.5, 55.4, 48.0; $\tilde{\nu}_{max}$ /cm$^{-1}$ (ATR); 3049, 2935, 1608, 1585, 1531, 1509, 1479, 1459, 1442, 1417, 1292, 1241, 1171, 1147, 1107, 1082; *m/z* (NSI) Found 345.1600 ([M+H]$^+$ $C_{22}H_{21}N_2O_2$ requires 345.1603).

### 1-(4-Chlorobenzyl)-2-(4-chlorophenyl)-1*H*-benzo[*d*]imidazole (03)[7]

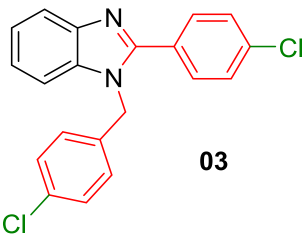

**03**

According to general procedure **A** and purification by automatic column chromatography (9:1 Hexane : EtOAc; SNAP KP-Sil 25 g column), compound **03** was obtained as a colourless solid (0.17 g, 95%); m.p. 138-140 °C (lit.[7] 138-140 °C); $^1$H NMR (400 MHz, Chloroform-*d*) δ = 7.81 (dt, *J*=8.1, 1.0, 1H), 7.55 – 7.50 (m, 2H), 7.41 – 7.34 (m, 2H), 7.30 – 7.23 (m, 3H), 7.19 (dd, *J*=7.2, 1.0, 1H), 7.13 (dt, *J*=8.0, 1.1, 1H), 6.95 (dd, *J*=8.9, 2.2, 2H), 5.33 (s, 2H); $^{13}$C{1H} NMR (101 MHz, Chloroform-*d*) δ = 153.0, 143.3, 136.5, 136.1, 134.8, 134.0, 130.6, 129.5, 129.3, 128.5, 127.4, 123.6, 123.1, 120.3, 110.4, 47.9; $\tilde{\nu}_{max}$ /cm$^{-1}$ (ATR); 3034, 2927, 1612, 1598, 1572, 1489, 1471, 1455, 1440, 1405, 1294, 1250, 743, 728; *m/z* (NSI) Found 353.0610 ([M+H]$^+$ $C_{20}H_{15}Cl_2N_2$ requires 353.0613).

**1-(4-Fluorobenzyl)-2-(4-fluorophenyl)-1*H*-benzo[*d*]imidazole (04)**[8]

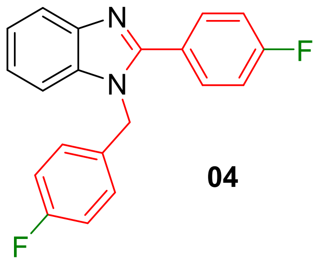

**04**

According to general procedure **A** and purification by automatic column chromatography (9:1 Hexane : EtOAc; SNAP KP-Sil 25 g column), compound **04** was obtained as a colourless solid (0.15 g, 93%); m.p. 114-116 °C (lit.[8] 114-116 °C); $^1$H NMR (400 MHz, Chloroform-*d*) δ = 7.77 (dt, *J*=8.0, 0.9, 1H), 7.59 – 7.51 (m, 2H), 7.25 – 7.04 (m, 5H), 6.99 – 6.89 (m, 4H), 5.31 (s, 2H); $^{13}$C{1H} NMR (101 MHz, Chloroform-*d*) δ = 165.1, 163.7, 162.7, 161.2, 153.2, 143.2, 136.0, 132.1, 131.4, 131.3, 127.8, 127.7, 126.4, 126.3, 123.4, 123.0, 120.2, 116.4, 116.2, 116.1, 116.0, 110.4, 47.8; $\tilde{\nu}_{max}$ /cm$^{-1}$ (ATR) 3075, 2922, 1606, 1509, 1481, 1460, 1444, 1413, 1280, 1220, 1095, 1016; *m/z* (NSI) Found 321.1200 ($[M+H]^+$ $C_{20}H_{15}F_2N_2$ requires 321.1203).

**1-(4-Methoxybenzyl)-2-(4-methoxyphenyl)-5,6-dimethyl-1*H*-benzo[*d*]imidazole (05)**[9]

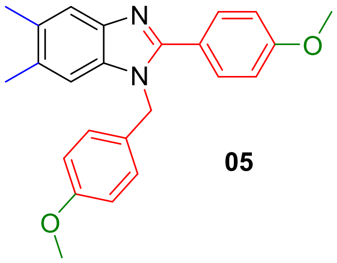

**05**

According to general procedure **A** and purification by automatic column chromatography (9:1 Hexane : EtOAc; SNAP KP-Sil 25 g column), compound **05** was obtained as a colourless solid (0.18 g, 99%); m.p. 184-186 °C (lit.[9] 180-181 °C); $^1$H NMR (500 MHz, Chloroform-*d*) δ = 7.62 – 7.58 (m, 3H), 7.03 (d, *J*=8.7, 2H), 6.97 – 6.93 (m, 3H), 6.86 (d, *J*=8.7, 2H), 5.33 (s, 2H), 3.84 (s, 3H), 3.79 (s, 3H), 2.38 (s, 3H), 2.33 (s, 3H); $^{13}$C{1H} NMR (126 MHz, Chloroform-*d*) δ = 160.8, 159.2, 153.4, 141.9, 134.8, 132.0, 131.5, 130.7, 128.9, 127.3, 122.8, 119.9, 114.5, 114.2, 110.6, 55.5, 55.4, 47.9, 20.7, 20.5; $\tilde{\nu}_{max}$ /cm$^{-1}$ (ATR) 3033, 2934, 1609, 1580, 1536, 1510, 1480, 1464, 1443, 1415, 1294, 1244, 1172, 1110, 1064; *m/z* (NSI) Found 373.1912 ($[M+H]^+$ $C_{24}H_{25}N_2O_2$ requires 373.1916).

**1-(4-Fluorobenzyl)-2-(4-fluorophenyl)-5,6-dimethyl-1*H*-benzo[*d*]imidazole (06)**

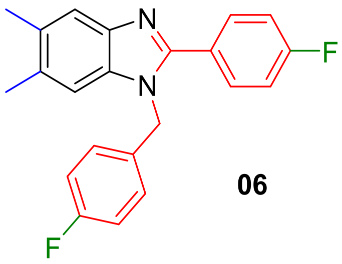

**06**

According to general procedure **A** and purification by automatic column chromatography (9:1 Hexane : EtOAc; SNAP KP-Sil 25 g column), compound **06** was obtained as a colourless solid (0.16 g, 92%); m.p. 150-152 °C; $^{1}$H NMR (400 MHz, Chloroform-*d*) δ = 7.61 (tt, *J*=5.0, 2.5, 3H), 7.16 – 7.08 (m, 2H), 7.08 – 6.93 (m, 5H), 5.34 (s, 2H), 2.39 (s, 3H), 2.34 (s, 3H); $^{13}$C{1H} NMR (101 MHz, Chloroform-*d*) δ = 141.9, 134.7, 132.6, 131.9, 131.3, 131.2, 127.7, 127.7, 120.3, 116.31, 116.1, 116.1, 115.9, 110.5, 47.7, 20.7, 20.4; $\tilde{\nu}_{max}$ /cm$^{-1}$ (ATR); 3040, 2941, 1606, 1595, 1534, 1511, 1480, 1467, 1439, 1424, 1411, 1299, 1229, 1096, 1059; *m/z* (NSI) Found 349.1510 ([M+H]$^{+}$ $C_{22}H_{19}F_{2}N_{2}$ requires 349.1516).

**1-(4-Chloro-3-fluorobenzyl)-2-(4-chloro-3-fluorophenyl)-5,6-dimethyl-1*H*-benzo[*d*]imidazole (07)**

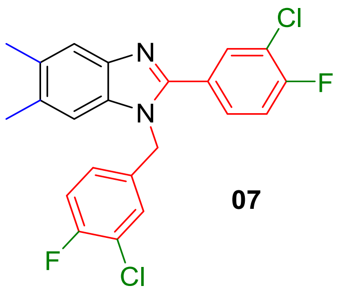

**07**

According to general procedure **A** and purification by automatic column chromatography (9:1 Hexane : EtOAc; SNAP KP-Sil 25 g column), compound **07** was obtained as a colourless solid (0.19 g, 89%); m.p. 171-173 °C; $^{1}$H NMR (400 MHz, Chloroform-*d*) δ = 7.73 (dd, *J*=7.0, 2.2, 1H), 7.61 (s, 1H), 7.43 (ddd, *J*=8.5, 4.5, 2.2, 1H), 7.21 – 7.09 (m, 3H), 6.97 (s, 1H), 6.90 (ddd, *J*=8.5, 4.3, 2.3, 1H), 5.32 (s, 2H), 2.40 (s, 3H), 2.36 (s, 3H); $^{13}$C{1H} NMR (101 MHz, Chloroform-*d*) δ = 156.6, 150.9, 141.7, 134.5, 133.5, 133.4, 133.4, 132.5, 131.8, 128.9, 128.8, 128.3, 127.5, 127.4, 125.7, 125.7, 122.3, 122.2, 122.1, 122.0, 120.4, 117.6, 117.4, 117.3, 117.1, 110.4, 47.4, 20.8, 20.4; $\tilde{\nu}_{max}$ /cm$^{-1}$ (ATR); 3022, 2925, 1602, 1523, 1497, 1477, 1463, 1446, 1477, 1463, 1446, 1428, 1414, 1405, 1264, 1247, 1073, 1054, 841, 816; *m/z* (NSI) Found 417.0731 ([M+H]$^{+}$ $C_{22}H_{17}Cl_{2}F_{2}N_{2}$ requires 417.0737).

### 1-Benzyl-6-chloro-2-phenyl-1*H*-benzo[*d*]imidazole (08)[10]

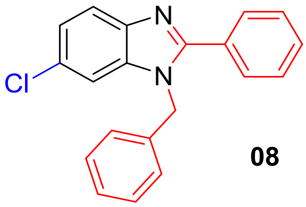


**08**

According to general procedure **A** and purification by automatic column chromatography (4:1 Hexane : EtOAc; SNAP KP-Sil 25 g column), compound **08** was obtained as a colourless solid (0.14 g, 85%); m.p. 160-162 °C (lit.[10] 159-161 °C); $^1$H NMR (400 MHz, Chloroform-*d*) δ = 7.83 (d, *J*=1.8, 1H), 7.69 – 7.66 (m, 2H), 7.47 (qd, *J*=6.2, 5.4, 2.1, 3H), 7.37 – 7.29 (m, 3H), 7.20 (dd, *J*=8.6, 1.9, 1H), 7.12 – 7.06 (m, 3H), 5.44 (s, 2H); $^{13}$C{1H} NMR (126 MHz, Chloroform-*d*) δ = 144.1, 136.1, 134.8, 130.4, 129.8, 129.4, 129.3, 129.0, 128.4, 128.1, 126.0, 123.6, 119.9, 111.5, 48.7; $\tilde{\nu}_{max}$/cm$^{-1}$ (ATR); 3047, 2929, 1606, 1591, 1577, 1551, 1517, 1496, 1469, 1453, 1426, 1283, 1256, 794, 774; *m/z* (NSI) Found 319.1000 ([M+H]$^+$ $C_{20}H_{16}ClN_2$ requires 319.1002).

### 6-Chloro-1-(4-methoxybenzyl)-2-(4-methoxyphenyl)-1*H*-benzo[*d*]imidazole (09)

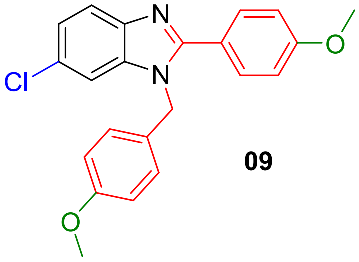


**09**

According to general procedure **A** and purification by automatic column chromatography (9:1 Hexane : EtOAc; SNAP KP-Sil 25 g column), compound **09** was obtained as a colourless solid (0.17 g, 88%); m.p. 142-144 °C; $^1$H NMR (400 MHz, Chloroform-*d*) δ = 7.80 (d, *J*=1.7, 1H), 7.62 (d, *J*=8.7, 2H), 7.17 (dd, *J*=8.6, 1.8, 1H), 7.10 (d, *J*=8.5, 1H), 7.02 – 6.95 (m, 4H), 6.88 – 6.83 (m, 2H), 5.36 (s, 2H), 3.85 (s, 3H), 3.78 (s, 3H); $^{13}$C{1H} NMR (101 MHz, Chloroform-*d*) δ = 161.3, 134.8, 130.8, 128.3, 128.1, 123.3, 119.6, 114.7, 114.4, 111.3, 55.5, 55.4, 48.2; $\tilde{\nu}_{max}$/cm$^{-1}$ (ATR); 3036, 2928, 1609, 1578, 1532, 1510, 1479, 1463, 1443, 1413, 1291, 1244, 1172, 1155, 1110, 1080, 1057, 791; *m/z* (NSI) Found 379.1207 ([M+H]$^+$ $C_{22}H_{20}ClN_2O_2$ requires 379.1213).

**6-Chloro-1-(4-chlorobenzyl)-2-(4-chlorophenyl)-1*H*-benzo[*d*]imidazole (10)**

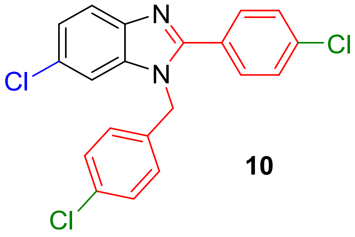


**10**

According to general procedure **A** and purification by automatic column chromatography (9:1 Hexane : EtOAc; SNAP KP-Sil 25 g column), compound **10** was obtained as a colourless solid (0.17 g, 86%); m.p. 161-163 °C; $^1$H NMR (400 MHz, Chloroform-*d*) δ = 7.83 (d, *J*=1.9, 1H), 7.59 – 7.56 (m, 2H), 7.46 – 7.43 (m, 2H), 7.34 – 7.30 (m, 2H), 7.23 (dd, *J*=8.6, 1.9, 1H), 7.10 (d, *J*=8.6, 1H), 6.99 (d, *J*=8.5, 2H), 5.38 (s, 2H); $^{13}$C{1H} NMR (126 MHz, Chloroform-*d*) δ = 154.2, 144.1, 136.8, 134.7, 134.3, 134.2, 130.6, 129.6, 129.4, 128.8, 127.3, 124.1, 120.1, 111.3, 48.1; $\tilde{\nu}_{max}$ /cm$^{-1}$ (ATR); 3026, 2929, 1600, 1573, 1515, 1490, 1466, 1432, 1405, 1300, 1251, 832, 797; *m/z* (NSI) Found 387.0219 ([M+H]$^+$ $C_{20}H_{14}Cl_3N_2$ requires 387.0223).

**6-Chloro-1-(4-fluorobenzyl)-2-(4-fluorophenyl)-1*H*-benzo[*d*]imidazole (11)**

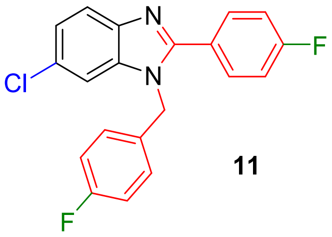


**11**

According to general procedure **A** and purification by automatic column chromatography (9:1 Hexane : EtOAc; SNAP KP-Sil 25 g column), compound **11** was obtained as a colourless solid (0.15 g, 83%); m.p. 182-184 °C; $^1$H NMR (400 MHz, Chloroform-*d*) δ = 7.82 (d, *J*=1.8, 1H), 7.66 – 7.60 (m, 2H), 7.24 – 7.09 (m, 4H), 7.03 (d, *J*=6.9, 4H), 5.38 (s, 2H); $^{13}$C{1H} NMR (126 MHz, Chloroform-*d*) δ = 163.5, 154.4, 144.0, 134.6, 131.6, 131.6, 131.4, 131.3, 128. 7, 127.7, 127.7, 125.9, 125.9, 123.9, 120.0, 116.5, 116.4, 116.3, 116.2, 111.3, 48.0; $\tilde{\nu}_{max}$ /cm$^{-1}$ (ATR); 3042, 2925, 1604, 1584, 1529, 1508, 1470, 1457, 1434, 1411, 1285, 1253, 1095, 1056, 808, 797, 778; *m/z* (NSI) Found 355.0810 ([M+H]$^+$ $C_{20}H_{14}ClF_2N_2$ requires 355.0814).

**6-Chloro-1-(3-chloro-4-fluorobenzyl)-2-(3-chloro-4-fluorophenyl)-1*H*-benzo[*d*]imidazole (12)**

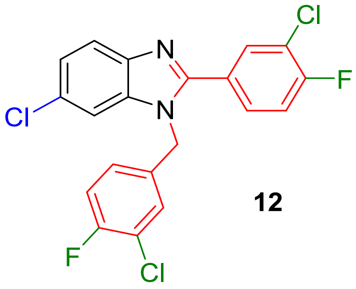
**12**

According to general procedure **A** and purification by automatic column chromatography (9:1 Hexane : EtOAc; SNAP KP-Sil 25 g column), compound **12** was obtained as a colourless solid (0.17 g, 79%); m.p. 158-160 °C; $^{1}$H NMR (500 MHz, Chloroform-*d*) δ = 7.83 (d, *J*=1.5, 1H), 7.75 (dd, *J*=6.9, 1.9, 1H), 7.47 (ddd, *J*=8.2, 4.3, 2.1, 1H), 7.34 – 7.27 (m, 1H), 7.26 – 7.18 (m, 1H), 7.14 – 7.11 (m, 3H), 6.91 – 6.88 (m, 1H), 5.36 (s, 2H); $^{13}$C{1H} NMR (126 MHz, Chloroform-*d*) δ = 157.0, 143.9, 134.5, 132.7, 132.7, 132.0, 129.1, 129.0, 128.9, 128.4, 126.8, 126.7, 125.7, 125.7, 124.5, 122.5, 122.5, 122.4, 122.3, 120.3, 117.8, 117.6, 117.5, 117.3, 47.6; $\tilde{\nu}_{max}$ /cm$^{-1}$ (ATR); 3069, 2926, 1601, 1586, 1523, 1497, 1468, 1455, 1433, 1281, 1248, 1086, 1056, 816, 797; *m/z* (NSI) Found 423.0019 ([M+H]$^{+}$ $C_{20}H_{12}Cl_3F_2N_2$ requires 423.0034).

**1-Benzyl-6-fluoro-2-phenyl-1*H*-benzo[*d*]imidazole (13)[11]**

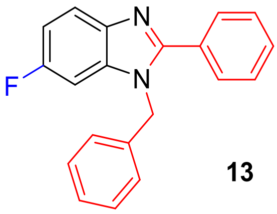
**13**

According to general procedure **A** and purification by automatic column chromatography (9:1 Hexane : EtOAc; SNAP KP-Sil 25 g column), compound **13** was obtained as a colourless solid (0.14 g, 91%); m.p. 131-133 °C (lit.[11] 132-134 °C); $^{1}$H NMR (400 MHz, Chloroform-*d*) δ = 7.68 (dd, *J*=7.7, 1.6, 2H), 7.54 – 7.43 (m, 4H), 7.38 – 7.28 (m, 3H), 7.10 (dd, *J*=7.9, 5.6, 3H), 6.98 (td, *J*=9.1, 2.4, 1H), 5.45 (s, 2H); $^{13}$C{1H} NMR (126 MHz, Chloroform-*d*) δ = 155.8, 136.2, 132.7, 130.3, 129.9, 129.3, 129.3, 129.0, 128.1, 126.1, 111.6, 111.4, 111.1, 111.0, 106.0, 105.8, 48.7; $\tilde{\nu}_{max}$ /cm$^{-1}$ (ATR); 3051, 2930, 1603, 1578, 1556, 1513, 1470, 1447, 1433, 1286, 1252, 1078, 1026; *m/z* (NSI) Found 303.1295 ([M+H]$^{+}$ $C_{20}H_{16}FN_2$ requires 303.1298).

## S6.0. Reactions of Yttrium Chloride Hexahydrate Impregnated Stirrer Devices

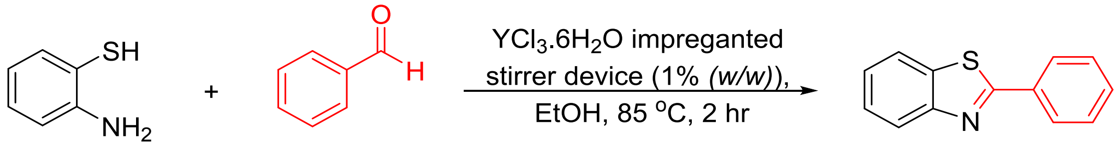


**14**

Chemical Formula: $C_6H_7NS$
Molecular Weight: 125.19

Chemical Formula: $C_7H_6O$
Molecular Weight: 106.12

Chemical Formula: $C_{13}H_9NS$
Molecular Weight: 211.28

An yttrium chloride hexahydrate impregnated 3D printed stirrer device was placed in a round bottom flask (25 mL) containing EtOH (10 mL) and heated to 85 °C using an Asynt DrySyn heating apparatus whilst stirring was set at 1000 RPM. Once the temperature reached 85 °C, 2-aminobenzenethiol (1.1 mmol, 0.138 g, 0.123 mL) and benzaldehyde (1.0 mmol, 0.106 g, 0.102 mL) were added to the reaction mixture. The progress of the reaction was monitored using TLC and the reaction was stopped after 95 minutes. The reaction mixture was cooled to room temperature and the stirrer device was washed with EtOH (1-2 mL). The combined reaction mixture was concentrated under reduced pressure and purified by column chromatography via Biotage (98:2 Hexane:EtOAc; Zip 30 g column) to give 2-phenylbenzo[*d*]thiazole **14** (0.18 g, 85%) as a colourless solid.

In order to scrutinize the progress of product formation over the course of the reaction, we accurately determined the % conversion of product using the LCMS. The first step was to construct a calibration curve.

Preparation of the calibration curve involved a two-step dilution, the first step proceeded as 2-phenylbenzo[*d*]thiazole (0.0105 g) was dissolved in EtOH (2 mL). An aliquot (400 µL) of this solution was dissolved in MeOH (600 µL) known as max product. Preparation of the internal standard also involved a two-step dilution, the first step proceeded as caffeine (0.050 g) was dissolved in EtOH (10 mL). An aliquot (100 µL) of this solution was dissolved in MeOH (900 µL). From this, subsequent solutions were prepared from 0-100%.

| Percentage product (%) | Max Product (μL) | Caffeine ((μL) | MeOH (μL) |
|---|---|---|---|
| 0 | 0 | 100 | 1000 |
| 10 | 10 | 100 | 990 |
| 20 | 20 | 100 | 980 |
| 30 | 30 | 100 | 970 |
| 40 | 40 | 100 | 960 |
| 50 | 50 | 100 | 950 |
| 60 | 60 | 100 | 940 |
| 70 | 70 | 100 | 930 |
| 80 | 80 | 100 | 920 |
| 90 | 90 | 100 | 910 |
| 100 | 100 | 100 | 900 |

**Supplementary Table 10:** The table displaying the construction of the calibration curve based on concentrations prepared from 0-100%

The percentage yield of 2-phenylbenzo[*d*]thiazole was determined by calculating the ratio of peak area of 2-phenylbenzo[*d*]thiazole and caffeine using the LCMS at 245 nm.

A few of the reactions was chosen to determine the % conversion of 2-phenylbenzo[*d*]thiazole and the rate of reaction.

**General Procedure B:** Caffeine (0.05 g, 0.26 mmol) was added to a round bottom flask (25 mL) containing ethanol (10 mL) and heated to 50 $^{o}$C for 15 minutes using an Asynt DrySyn heating apparatus while stirring at 1000 RPM using a conventional stirrer. The conventional stirrer was replaced with an yttrium chloride hexahydrate impregnated 3D stirrer and the temperature was increased to 85 $^{o}$C followed by the addition of 2-mercaptoaniline (0.123 mL, 1.1 mmol) and benzaldehyde (0.102 mL, 1.0 mmol). The reaction was followed by LCMS: first 5 minutes of the reaction then every 10 mins for 75 mins by preparing a sample of reaction mixture (10 μL) and LCMS MeOH (990 μL) each time. The percentage yield of 2-phenylbenzo[*d*]thiazole was determined by calculating the ratio of peak area of 2-phenylbenzo[*d*]thiazole and

caffeine using the LCMS at 245 nm and the calibration curve. This reaction was repeated another 2 times to obtain results in triplicates.

The general procedure **B** was repeated using:

- Powdered catalyst + conventional stirrer
- Powdered catalyst + blank 3D printed stirrer device
- no catalyst + conventional stirrer
- no catalyst + blank 3D printed stirrer device

The reusability test using the same yttrium chloride hexahydrate impregnated stirrer device was carried out up to three times to determine whether the device can maintain its catalytic activity.

Caffeine (0.05 g, 0.26 mmol) was added to a round bottom flask (25 mL) containing ethanol (10 mL) and heated to 50 $^{o}$C for 15 mins while stirring at 1000 RPM using a conventional stirrer. The conventional stirrer was replaced with an yttrium chloride hexahydrate impregnated 3D stirrer and the temperature was increased to 85 $^{o}$C followed by the addition of 2-mercaptoaniline (0.123 mL, 1.1 mmol) and benzaldehyde (0.102 mL, 1.0 mmol). The reaction was followed by LCMS: first 5 minutes of the reaction then every 10 mins for 75 mins by preparing a sample of reaction mixture (10 µL) and LCMS MeOH (990 µL) each time. The percentage yield of 2-phenylbenzo[*d*]thiazole was determined by calculating the ratio of peak area of 2-phenylbenzo[*d*]thiazole and caffeine using the LCMS at 245 nm and the calibration curve. In order to determine the reusability of the devices, this reaction was repeated another 2 times using the same yttrium chloride hexahydrate impregnated 3D stirrer device. After each use, the catalyst impregnated stirrer device was washed thoroughly with EtOH (1-2 mL), dried and used in the same reaction.

According to the LCMS trace of the reaction mixture at 35 minutes, the by-products are greatly diminished in the 3D printed stirrer device catalysed reaction compared to the traditional reaction involving the use of powdered catalysts (Supplementary Figure 12).

**A:** Powdered $YCl_3.6H_2O$ at t = 35 mins

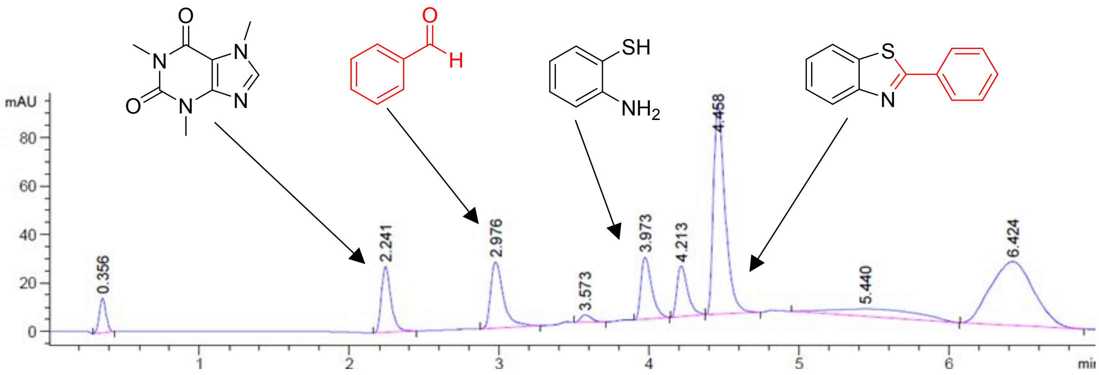


**B:** $YCl_3.6H_2O$ Impregnated 3D Printed Stirrer Device at t = 35 mins

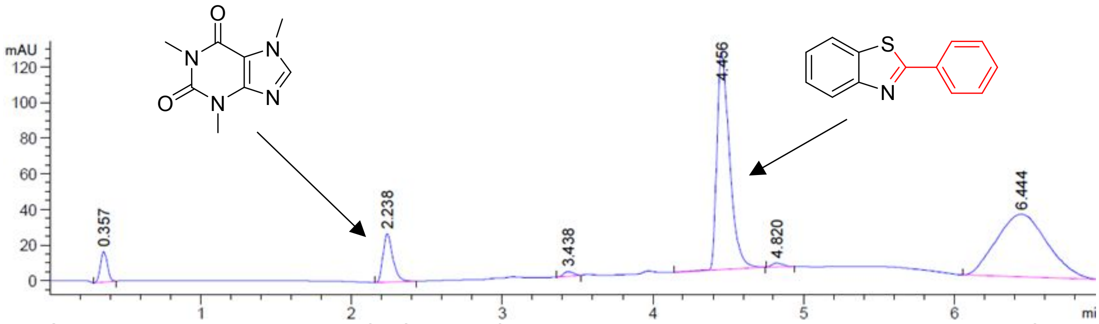


**Supplementary Figure 12:** LCMS trace of crude reaction mixture at 35 mins catalyzed by $YCl_3.6H_2O$. **A:** Powered $YCl_3.6H_2O$ **B:** $YCl_3.6H_2O$ impregnated stirrer devices.

| | Yield (%) calculated by LCMS |
|---|---|
| RUN 1 | 95 |
| RUN 2 | 92 |
| RUN 3 | 18 |

| | RUN 1 | RUN 2 | RUN 3 |
|---|---|---|---|
| Appearance of 3D Printed Stirrer Device | 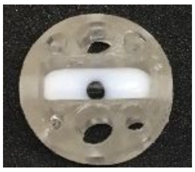 | 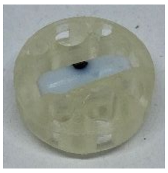 | 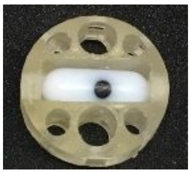 |
| Yield (%) | 95 | 92 | 18 |

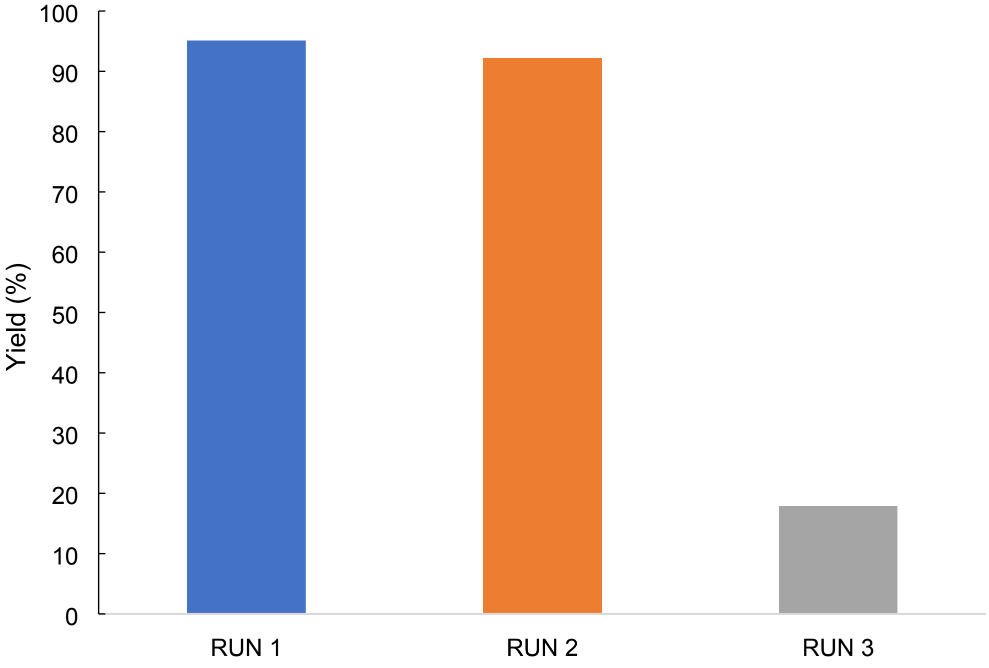


**Supplementary Table 11:** The reusability of a single yttrium chloride hexahydrate impregnated stirrer device in the reaction of *o*-aminothiaphenol and benzaldehyde.

In order to determine whether yttrium was being lost to the reaction through leaching or whether the reaction was taking place at the surface of the device, we carried out an analysis of metal leaching from the reaction, which was determined by ICP-OES.

**General Procedure B:** An yttrium chloride hexahydrate impregnated 3D printed stirrer device was placed in a round bottom flask (25 mL) containing EtOH (10 mL) and heated till the temperature reached 85 °C using an Asynt DrySyn heating apparatus while stirring at 1000 RPM. Once the temperature reached 85 °C, 2-aminobenzenethiol (1.1 mmol, 0.138 g, 0.123 mL) and benzaldehyde (1.0 mmol, 0.106 g, 0.102 mL) were added to the reaction mixture and was continued heating at 85 °C while stirring at 1000 RPM for 35 mins. The blank 3D printed stirrer device was removed and washed with EtOH before reducing under pressure. The crude mixture was placed on high vac to ensure that all the EtOH was removed. The crude mixture was dissolved in DCM and transferred into a glass vial. The vial was covered in foil with tiny holes for a slow evaporation. After the evaporation of DCM, the vial placed in a drying pistol under high vac to ensure all the DCM has evaporated.
Total mass of sample = 205.19 mg

The general procedure **B** was repeated using powdered catalyst + conventional stirrer.

Post drying, the remaining mass was digested in aqua regia (20 mL) at 110 °C for 1 hour. The digestate was made up to 50 mL in volume using deionised water then subsequently analysed by ICP-OES looking for yttrium. We scanned the following wavelengths: 371.030 nm, 360.073 and 377.433 nm. The results were blank corrected then latter converted from mg/L (the results provided by the instrument based on our calibration) to mg/kg using the initial mass of the samples and the volume of the digestate.

## S7.0. General Procedures: Benzothiazole Derivatives

### S7.1. General Procedure B: 2-phenylbenzo[*d*]thiazole (14)[12]

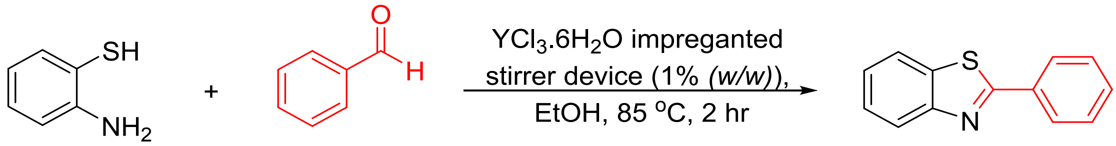


**14**

Chemical Formula: $C_6H_7NS$
Molecular Weight: 125.19

Chemical Formula: $C_7H_6O$
Molecular Weight: 106.12

Chemical Formula: $C_{13}H_9NS$
Molecular Weight: 211.28

An yttrium chloride hexahydrate catalyst impregnated 3D printed stirrer device was placed in a round bottom flask (25 mL) containing EtOH (10 mL) and heated till the temperature reached 85 °C using an Asynt DrySyn heating apparatus whilst stirring was set at 1000 RPM. Once the temperature reached 85 °C, 2-aminobenzenethiol (1.1 mmol, 0.138 g, 0.123 mL) and 4-methoxybenzaldehyde (1.0 mmol, 0.136 g, 1.22 mL) were added to the reaction mixture. Once complete, the reaction mixture was cooled to room temperature and concentrated under reduced pressure and the residue purified by column chromatography via Biotage (9:1 Hexane : EtOAc; Zip 10 g column) to give 2-(4-methoxyphenyl)benzo[d]thiazole **14** (0.18 g, 85%) as a colourless solid; m.p. 111-113 °C (lit.[12] 112-114 °C); $^1$H NMR (400 MHz, Chloroform-*d*) δ = 8.15 – 8.06 (m, 3H), 7.89 (d, *J*=8.0, 1H), 7.53 – 7.47 (m, 4H), 7.41 – 7.35 (m, 1H); $^{13}$C{1H} NMR (101 MHz, Chloroform-*d*) δ = 154.3, 135.2, 131.0, 129.1, 127.7, 126.4, 125.3, 123.3, 121.7; $\tilde{\nu}_{max}$ /cm$^{-1}$ (ATR) 3062, 1588, 1554, 1510, 1477, 1445, 1432; *m/z* (NSI) Found 212.0529 ([M+H]$^+$ $C_{13}H_{10}NS$ requires 212.0534).

### 2-(4-methoxyphenyl)benzo[*d*]thiazole (15)[13]

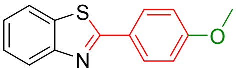


**15**

According to general procedure **B** and purification by automatic column chromatography (9:1 Hexane : EtOAc; Zip 10 g column), compound **15** was obtained as a colourless solid (0.22 g, 93%); m.p. 119-121 °C (lit.[13] 120-121 °C); $^1$H NMR (400 MHz, Chloroform-*d*) δ = 8.26 – 7.92 (m, 3H), 7.87 (ddd, *J*=8.0, 1.2, 0.6, 1H), 7.47 (ddd, *J*=8.3, 7.2, 1.3, 1H), 7.40 – 7.31 (m, 1H), 7.04 – 6.95 (m, 2H), 3.88 (s, 3H); $^{13}$C{1H} NMR (101 MHz, Chloroform-*d*) δ = 162.1, 154.4, 135.0, 129.3, 126.6, 126.3, 124.9, 123.0, 121.7, 114.5; $\tilde{\nu}_{max}$ /cm$^{-1}$ (ATR)

2995, 2922, 1604, 1557, 1521, 1483, 1455, 1433, 1411, 1114; *m/z* (ESI) Found 241.9 ($[M+H]^+$ $C_{14}H_{12}NOS$ requires 242.1).

**4-(Benzo[d]thiazol-2-yl)phenol (16)[14]**

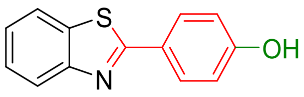

**16**

According to general procedure **B** and purification by automatic column chromatography (9:1 Hexane : EtOAc; Zip 10 g column), compound **16** was obtained as an off-white solid (0.22 g, 97%); m.p. 223-225 °C (lit.[14] 224-225 °C); $^1$H NMR (400 MHz, DMSO-$d_6$) δ = 10.23 (s, 1H), 8.07 – 8.01 (m, 1H), 8.00 – 7.96 (m, 1H), 7.96 – 7.90 (m, 2H), 7.48 (ddd, *J*=8.3, 7.3, 1.3, 1H), 7.38 (td, *J*=7.7, 7.3, 1.2, 1H), 6.97 – 6.93 (m, 2H, Ar-**H**); $^{13}$C{1H} NMR (101 MHz, DMSO-$d_6$) δ = 160.6, 153.8, 134.2, 129.1, 126.4, 124.9, 124.1, 122.3, 122.1, 116.1; $\tilde{\nu}_{max}$ /cm$^{-1}$ (ATR) 3628, 2999, 2943, 1604, 1586, 1521, 1481, 1454, 1428; *m/z* (ESI) Found 227.9 ($[M+H]^+$ $C_{13}H_{10}NOS$ requires 228.0).

**2-(4-Chlorophenyl)benzo[d]thiazole (17)[15]**

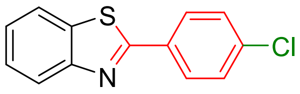

**17**

According to general procedure **B** and purification by automatic column chromatography (9:1 Hexane : EtOAc; Zip 10 g column), compound **17** was obtained as a colourless solid (0.22 g, 89%); m.p. 116-118 °C (lit.[15] 116-118 °C); $^1$H NMR (400 MHz, Chloroform-*d*) δ = 8.07 (ddd, *J*=8.2, 1.1, 0.6, 1H), 8.03 – 7.98 (m, 2H), 7.88 (ddd, *J*=8.0, 1.2, 0.6, 1H), 7.49 (ddd, *J*=8.3, 7.2, 1.3, 1H), 7.46 – 7.43 (m, 2H), 7.38 (ddd, *J*=8.3, 7.3, 1.2, 1H); $^{13}$C{1H} NMR (101 MHz, Chloroform-*d*) δ = 166.7, 154.2, 137.1, 135.2, 132.2, 129.4, 128.8, 126.6, 125.5, 123.4, 121.7; $\tilde{\nu}_{max}$ /cm$^{-1}$ (ATR) 3041, 2952, 1632, 1588, 1556, 1505, 1474, 1456, 1434, 756; *m/z* (ESI) Found 245.8 ($[M+H]^+$ $C_{13}H_9ClNS$ requires 246.0).

**2-(4-Fluorophenyl)benzo[d]thiazole (18)[16]**

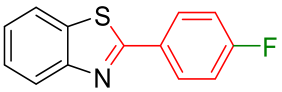

**18**

According to general procedure **B** and purification by automatic column chromatography (9:1 Hexane : EtOAc; Zip 10 g column), compound **18** was obtained as a colourless solid (0.20 g, 87%); m.p. 100-102 °C (lit.[16] 100-102 °C); $^1$H NMR (400 MHz, Chloroform-*d*) δ = 8.09 – 8.00 (m, 3H), 7.84 (d, *J*=8.0, 1H), 7.51 – 7.45 (m, 1H), 7.38 – 7.32 (m, 1H), 7.17 – 7.11 (m, 2H); $^{13}$C{1H} NMR (101 MHz, Chloroform-*d*) δ = 166.7, 163.2, 154.1, 135.1, 130.0, 129.5, 126.4, 125.2, 123.2, 121.6, 116.0; $\tilde{\nu}_{max}$/cm$^{-1}$ (ATR) 3051, 2922, 1600, 1556, 1519, 1479, 1455, 1434, 1407, 1226; *m/z* (ESI) Found 229.9 ($[M+H]^+$ $C_{13}H_9FNS$ requires 230.0).

**2-(3-chloro-4-Fluorophenyl)benzo[*d*]thiazole (19)**

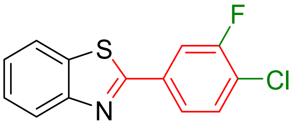

**19**

According to general procedure **B** and purification by automatic column chromatography (9:1 Hexane : EtOAc; Zip 10 g column), compound **19** was obtained as a colourless solid (0.20 g, 74%); m.p. 141-143 °C; $^1$H NMR (400 MHz, Chloroform-*d*) δ = 8.20 (dd, *J*=7.0, 2.2, 1H), 8.07 (ddd, *J*=8.2, 1.1, 0.6, 1H), 7.97 – 7.90 (m, 2H), 7.52 (ddd, *J*=8.3, 7.2, 1.3, 1H), 7.42 (ddd, *J*=8.3, 7.3, 1.2, 1H), 7.28 (d, *J*=8.6, 1H); $^{13}$C{1H} NMR (126 MHz, Chloroform-*d*) δ = 165.4, 158.8, 154.1, 135.3, 131.1, 131.0, 129.9, 127.6, 127.5, 126.8, 125.8, 123.5, 122.4, 122.2, 121.9, 117.5, 117.3; $\tilde{\nu}_{max}$/cm$^{-1}$ (ATR) 3046, 2924, 1600, 1557, 1514, 1474, 1457, 1434, 1402, 1226, 1215, 754; *m/z* (ESI) Found 263.8 ($[M+H]^+$ $C_{13}H_8ClFNS$ requires 264.0).

**2-(4-Nitrophenyl)benzo[d]thiazole (20)[17]**

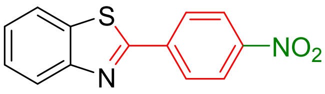

**20**

According to general procedure **B** and purification by automatic column chromatography (9:1 Hexane : EtOAc; Zip 10 g column), compound **20** was obtained as a colourless solid (0.22 g, 85%); m.p. 230-232 °C (lit.[17] 230-232 °C); $^1$H NMR (500 MHz, Chloroform-*d*) δ = 8.37 – 8.33 (m, 2H), 8.29 – 8.24 (m, 2H), 8.13 (d, *J*=8.2, 1H), 7.96 (d, *J*=8.0, 1H), 7.58 – 7.54 (m, 1H), 7.49 – 7.45 (m, 1H); $^{13}$C{1H} NMR (126 MHz, Chloroform-d) δ = 165.0, 154.3, 149.2, 139.3, 127.1, 126.4, 124.5, 124.1,

122.0; $\tilde{\nu}_{max}$ /cm$^{-1}$ (ATR) 3059, 2927, 1638, 1572, 1518, 1486, 1435, 1422, 1248, 1112; *m/z* (ESI) Found 256.8 ($[M+H]^+$ $C_{13}H_9N_2O_2S$ requires 257.0).

### 5-Chloro-2-phenylbenzo[d]thiazole (21)[18]

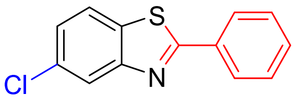

**21**

According to general procedure **B** and purification by automatic column chromatography (9:1 Hexane : EtOAc; Zip 10 g column), compound **21** was obtained as a colourless solid (0.20 g, 81%); m.p. 136-138 °C (lit.[18] 135-137 °C); $^1$H NMR (400 MHz, Chloroform-*d*) δ = 8.11 – 8.04 (m, 3H), 7.83 – 7.79 (m, 1H), 7.53 – 7.48 (m, 3H), 7.36 (dd, *J*=8.5, 2.0, 1H); $^{13}$C{1H} NMR (101 MHz, Chloroform-*d*) δ = 170.1, 155.1, 133.5, 133.4, 132.5, 131.5, 129.2, 127.8, 125.8, 123.2, 122.4; $\tilde{\nu}_{max}$ /cm$^{-1}$ (ATR) 3080, 2919, 1612, 1582, 1542, 1505, 1476, 1445, 1431, 1409, 687, 762; *m/z* (ESI) Found 245.8 ($[M+H]^+$ $C_{13}H_9ClNS$ requires 246.0).

### 5-Chloro-2-(4-methoxyphenyl)benzo[d]thiazole (22)[19]

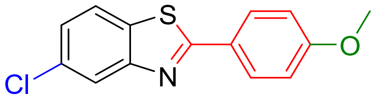

**22**

According to general procedure **B** and purification by automatic column chromatography (9:1 Hexane : EtOAc; Zip 10 g column), compound **22** was obtained as a colourless solid (0.24 g, 86%); m.p. 149-151 °C (lit.[19] 149-150 °C); $^1$H NMR (400 MHz, Chloroform-*d*) δ = 8.10 – 7.90 (m, 3H), 7.76 (d, *J*=8.5, 1H), 7.31 (dd, *J*=8.5, 2.0, 1H), 7.01 – 6.97 (m, 2H), 3.88 (s, 3H); $^{13}$C{1H} NMR (101 MHz, Chloroform-*d*) δ = 169.8, 162.3, 155.2, 133.2, 132.3, 129.3, 126.2, 122.7, 122.3, 114.5, 55.6; $\tilde{\nu}_{max}$ /cm$^{-1}$ (ATR) 2961, 2923, 1600, 1542, 1475, 1430, 1111, 882, 829, 805; *m/z* (ESI) Found 275.8 ($[M+H]^+$ $C_{14}H_{11}ClNOS$ requires 276.0).

**5-Chloro-2-(4-fluorophenyl)benzo[d]thiazole (23)[20]**

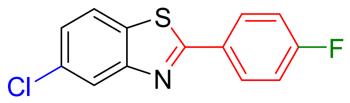


**23**

According to general procedure **B** and purification by automatic column chromatography (9:1 Hexane : EtOAc; Zip 10 g column), compound **23** was obtained as a colourless solid (0.19 g, 71%) as a colourless solid; m.p. 113-115 °C (lit.[6] 112-115 °C); $^{1}$H NMR (400 MHz, Chloroform-*d*) δ = 8.10 – 8.03 (m, 3H), 7.82 – 7.78 (m, 1H), 7.37 (dd, *J*=8.5, 2.0, 1H), 7.22 – 7.17 (m, 2H); $^{13}$C{1H} NMR (126 MHz, Chloroform-*d*) δ = 168.7, 165.8, 133.4, 132.6, 129.8, 129.8, 125.9, 123.2, 122.5, 116.5, 116.4; $\tilde{\nu}_{max}$ /cm$^{-1}$ (ATR) 3029, 2920, 1620, 1599, 1546, 1522, 1479, 1434, 1409, 1235, 839, 813; *m/z* (ESI) Found 263.8 ([M+H]$^{+}$ $C_{13}H_{8}ClFNS$ requires 264.0).

# S8.0. NMR Spectra: Benzimidazole Derivatives

## $^1$H NMR (400 MHz, Chloroform-*d*)

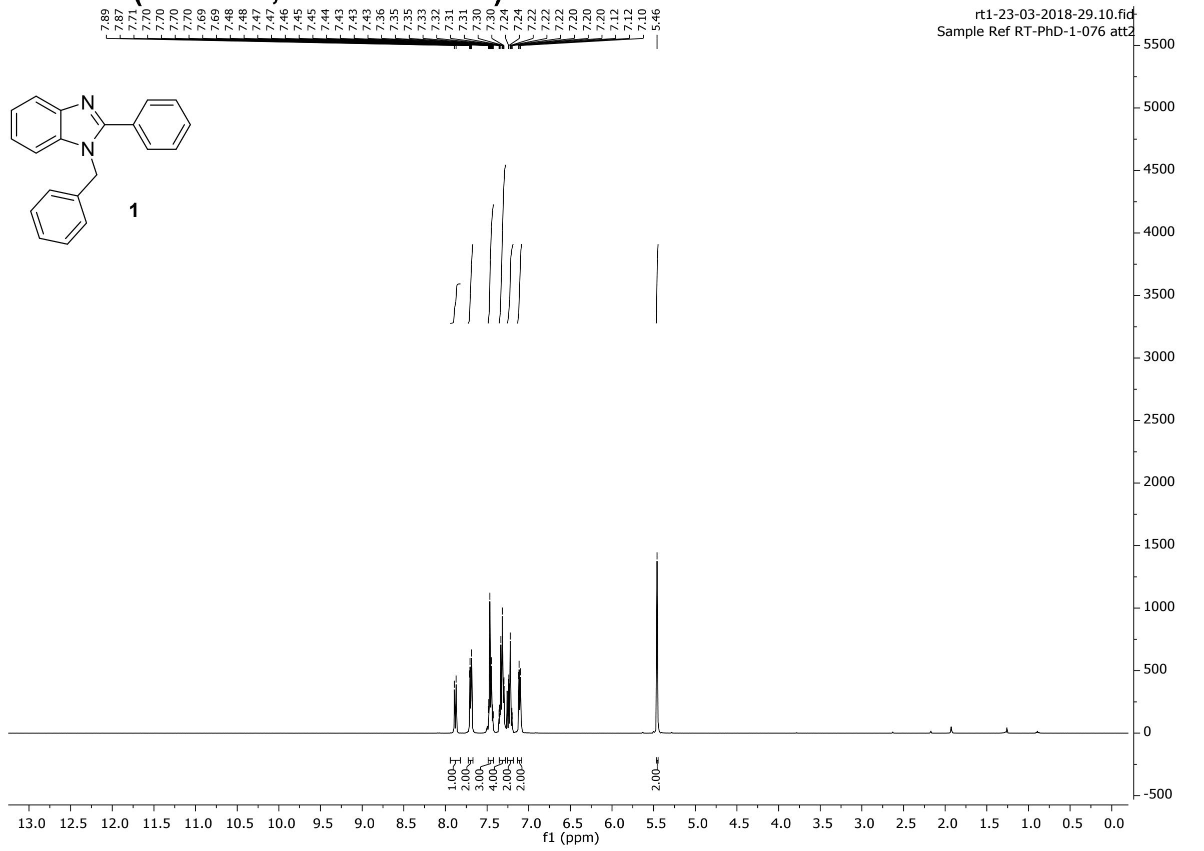


## $^{13}$C{1H} NMR (101 MHz, Chloroform-*d*)

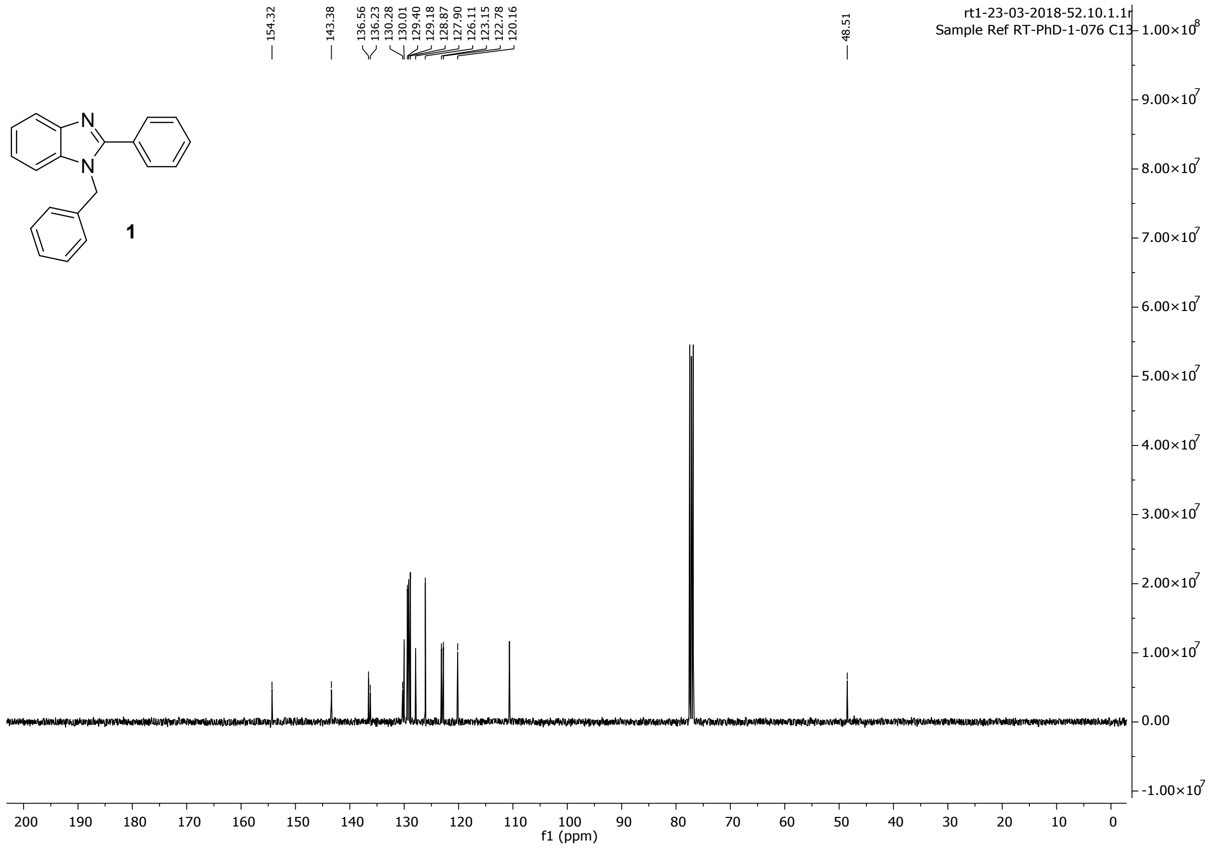

## $^{1}$H NMR (400 MHz, Chloroform-*d*)

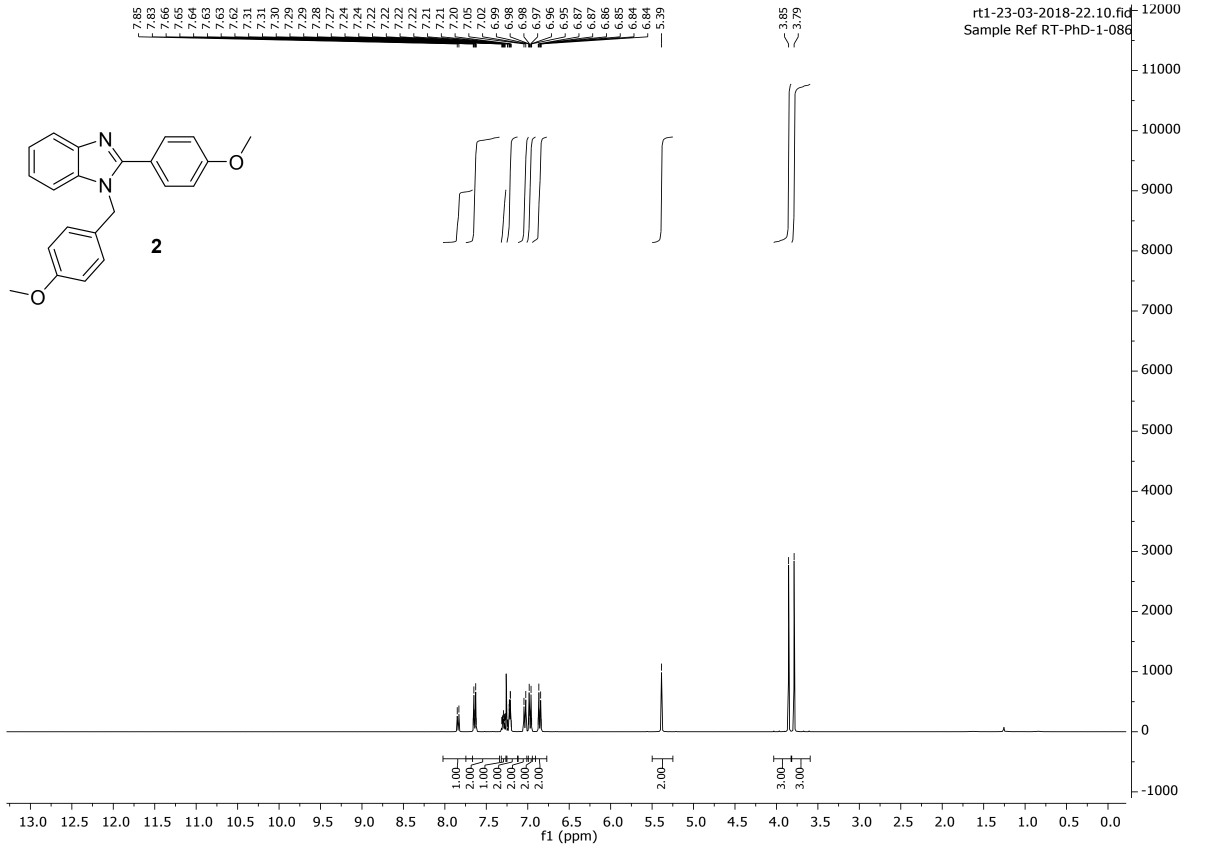


## $^{13}$C{1H} NMR (101 MHz, Chloroform-*d*)

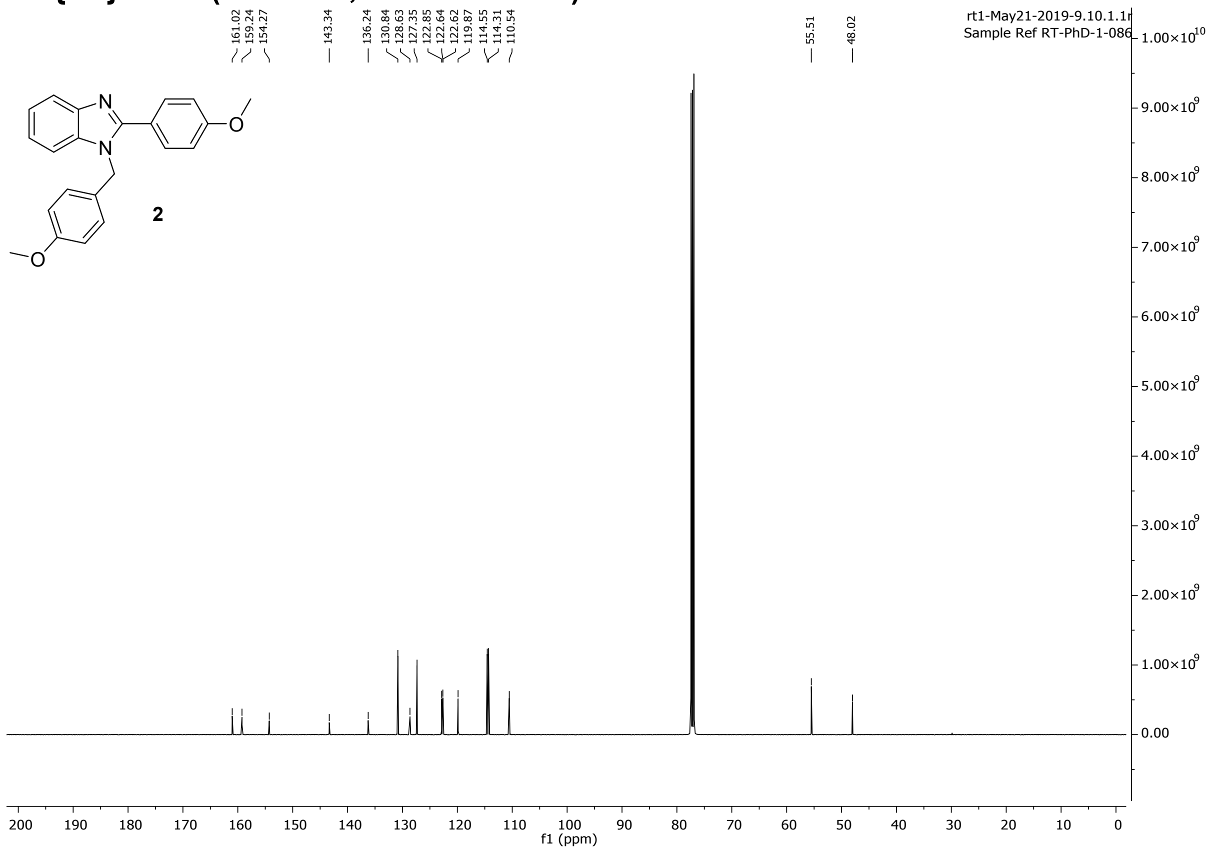

## $^1H$ NMR (400 MHz, Chloroform-*d*)

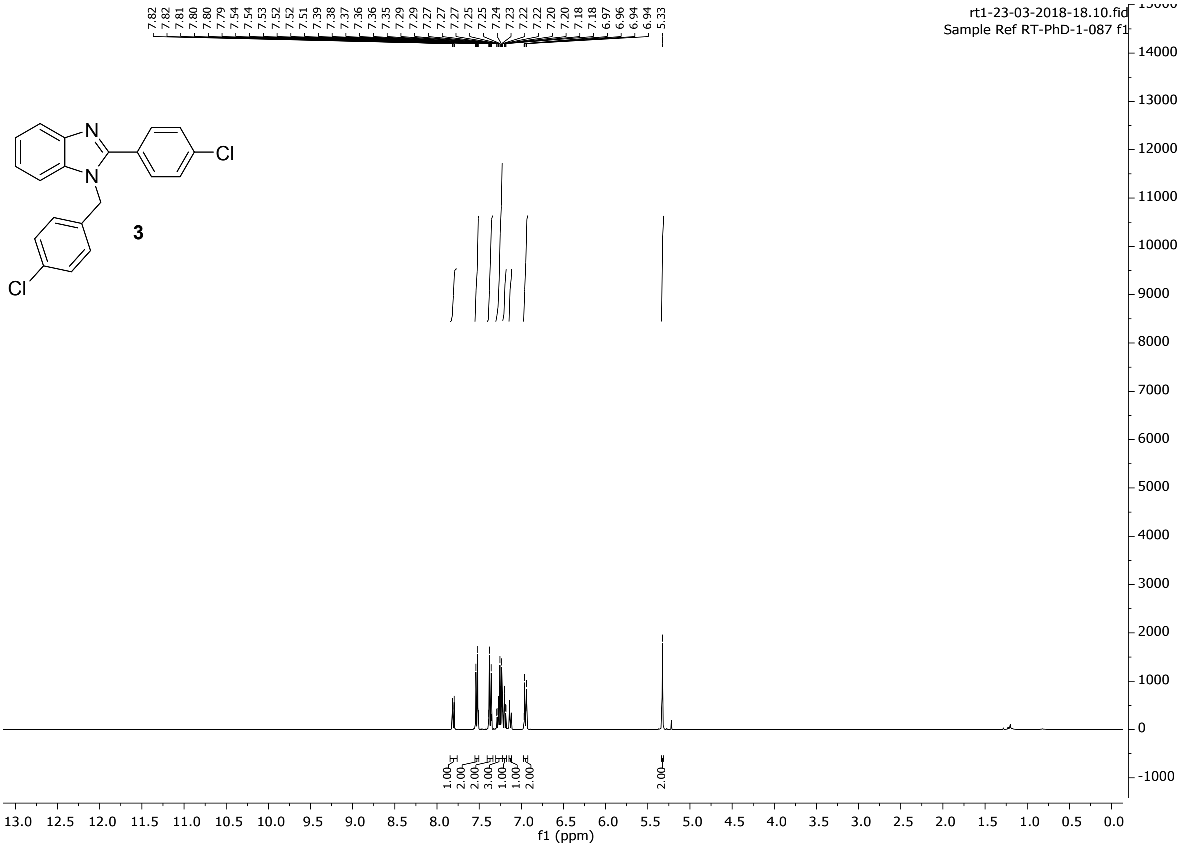


## $^{13}C\{1H\}$ NMR (101 MHz, Chloroform-*d*)

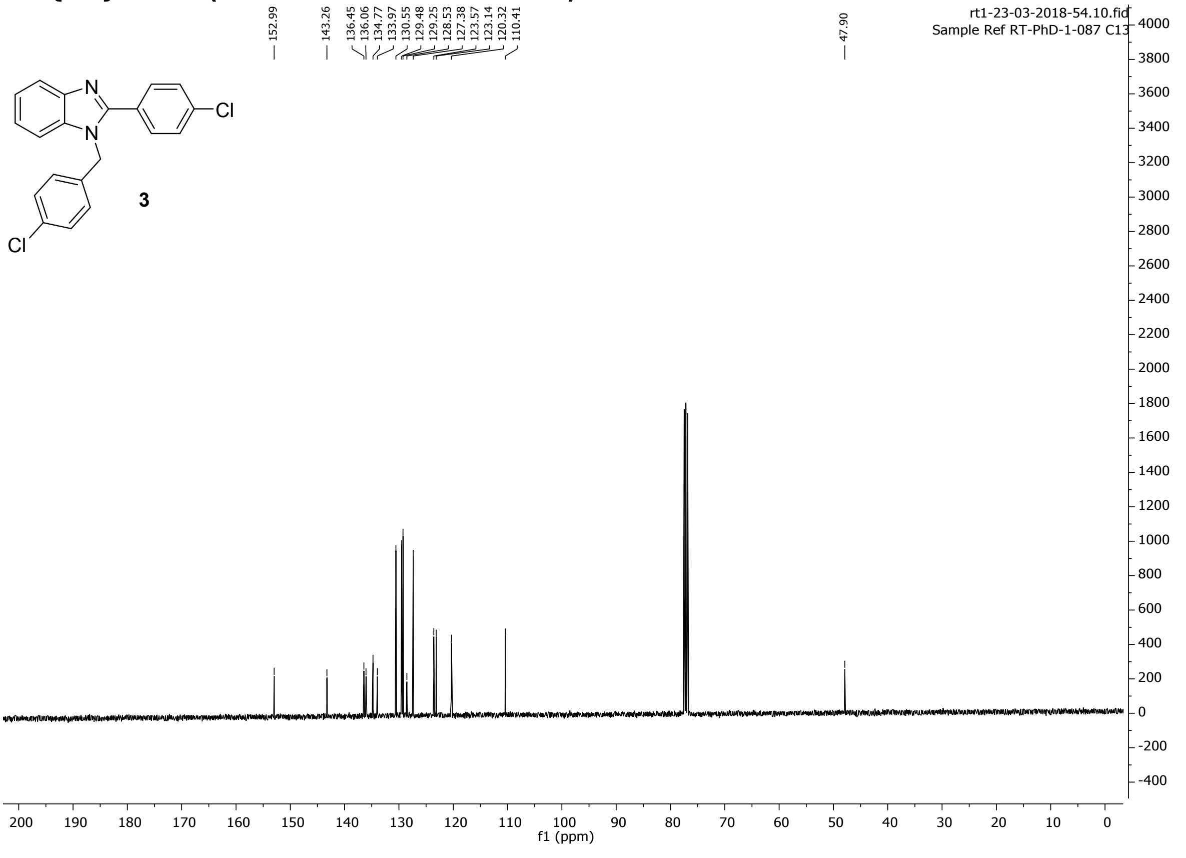

## $^{1}$H NMR (400 MHz, Chloroform-*d*)

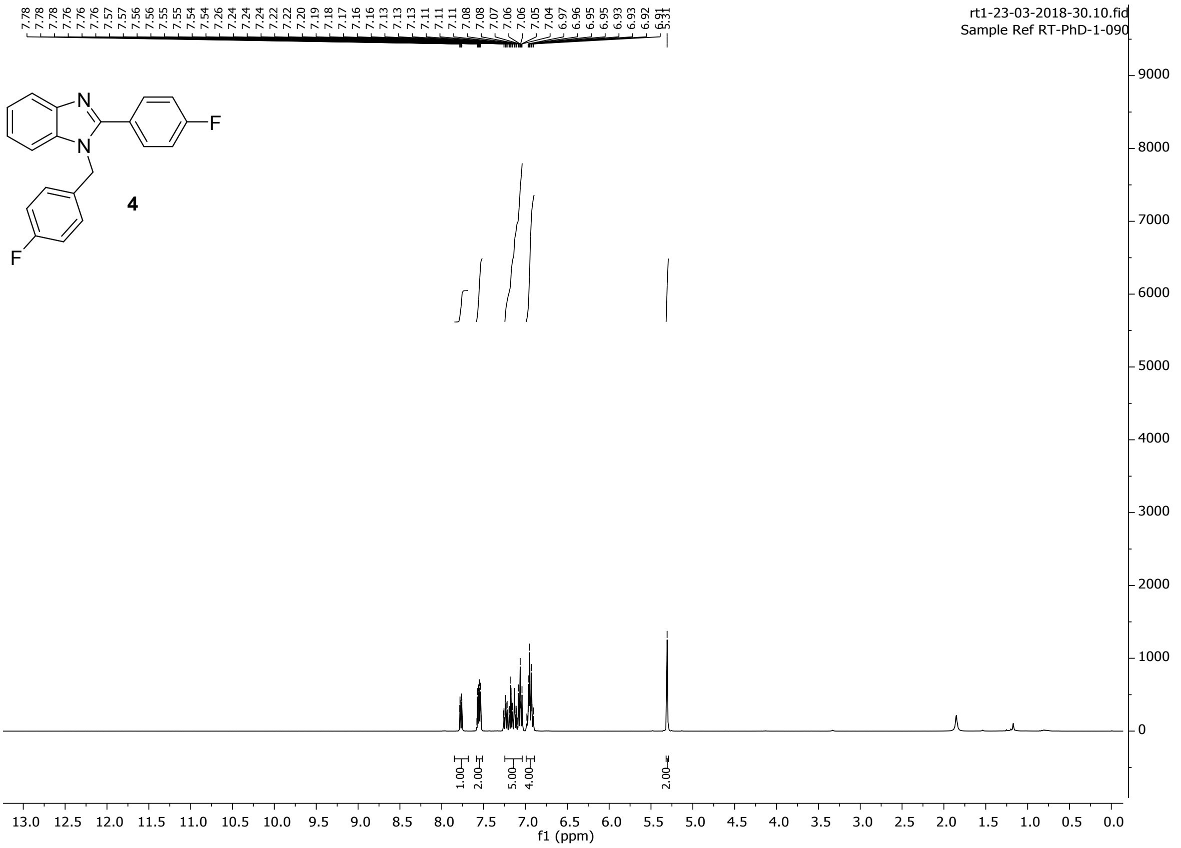


## $^{13}$C{1H} NMR (101 MHz, Chloroform-*d*)

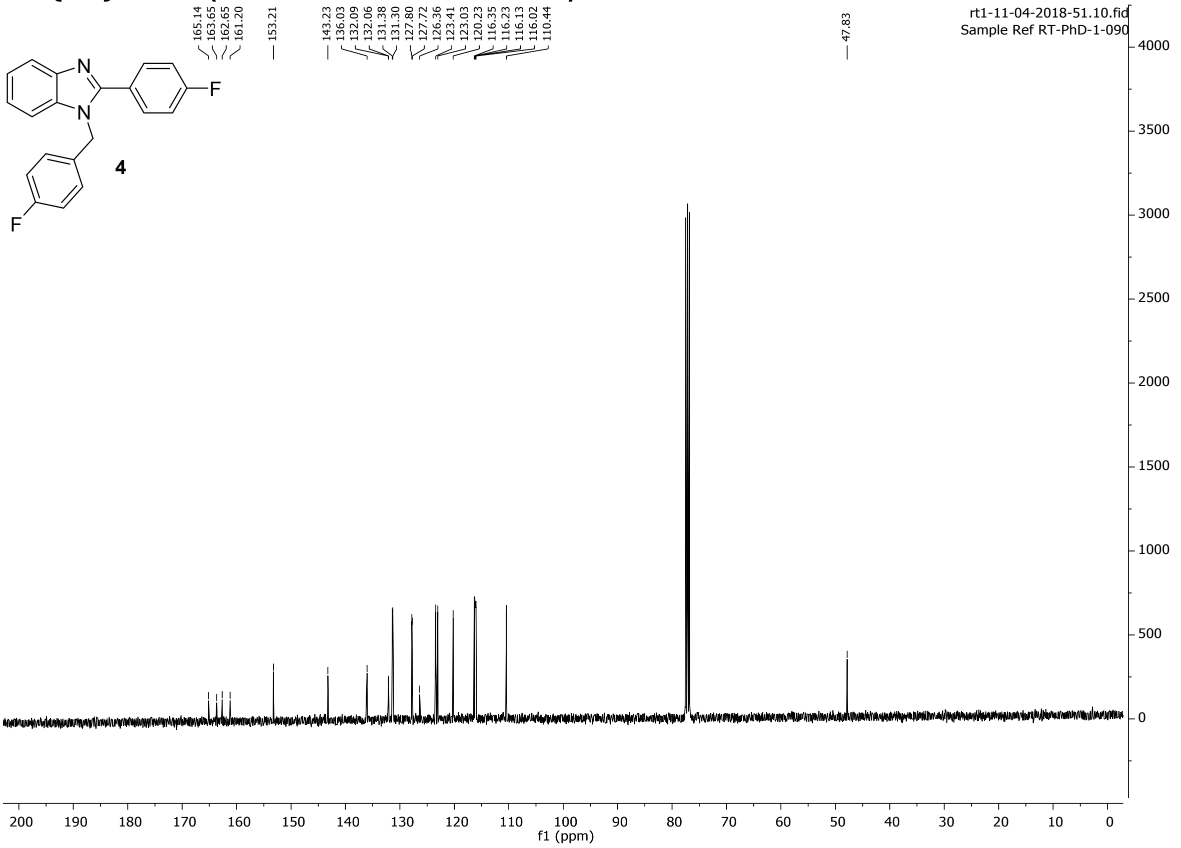

## $^{1}$H NMR (500 MHz, Chloroform-*d*)

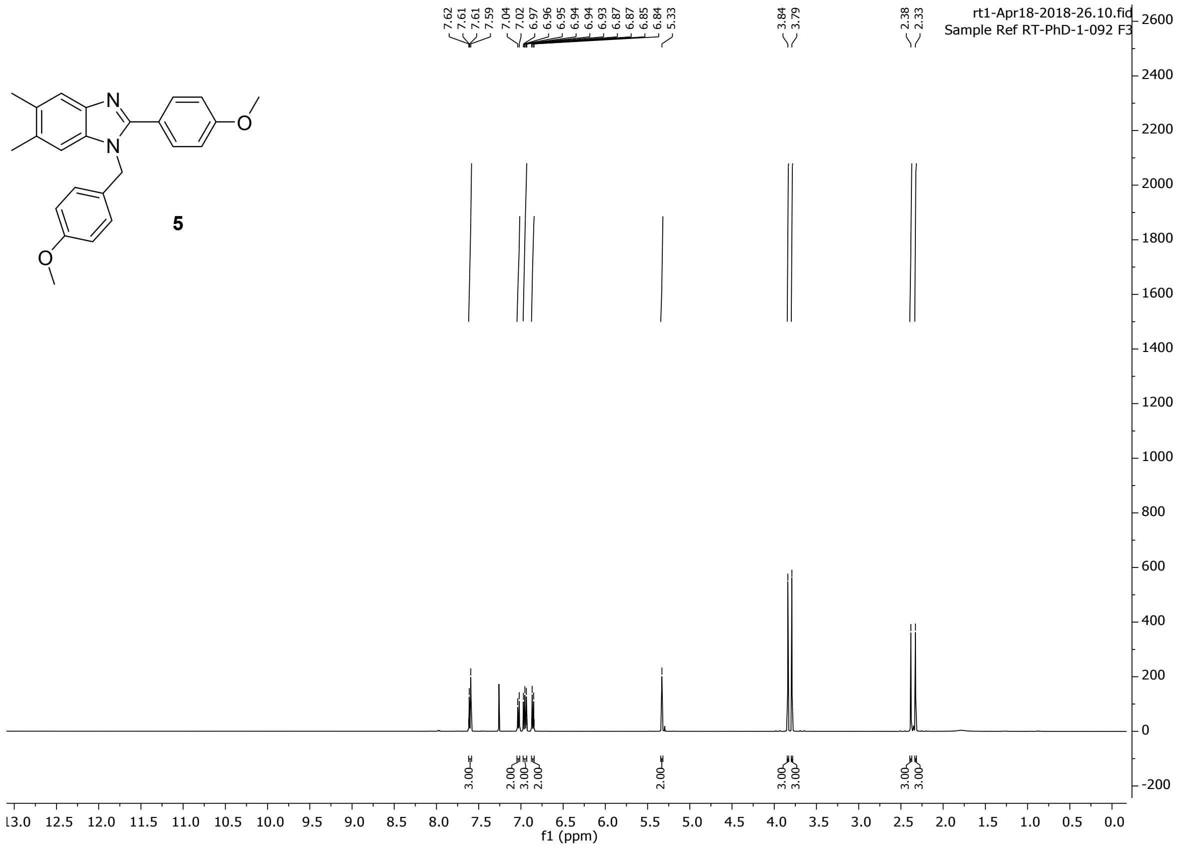

## $^{13}$C{1H} NMR (126 MHz, Chloroform-*d*)

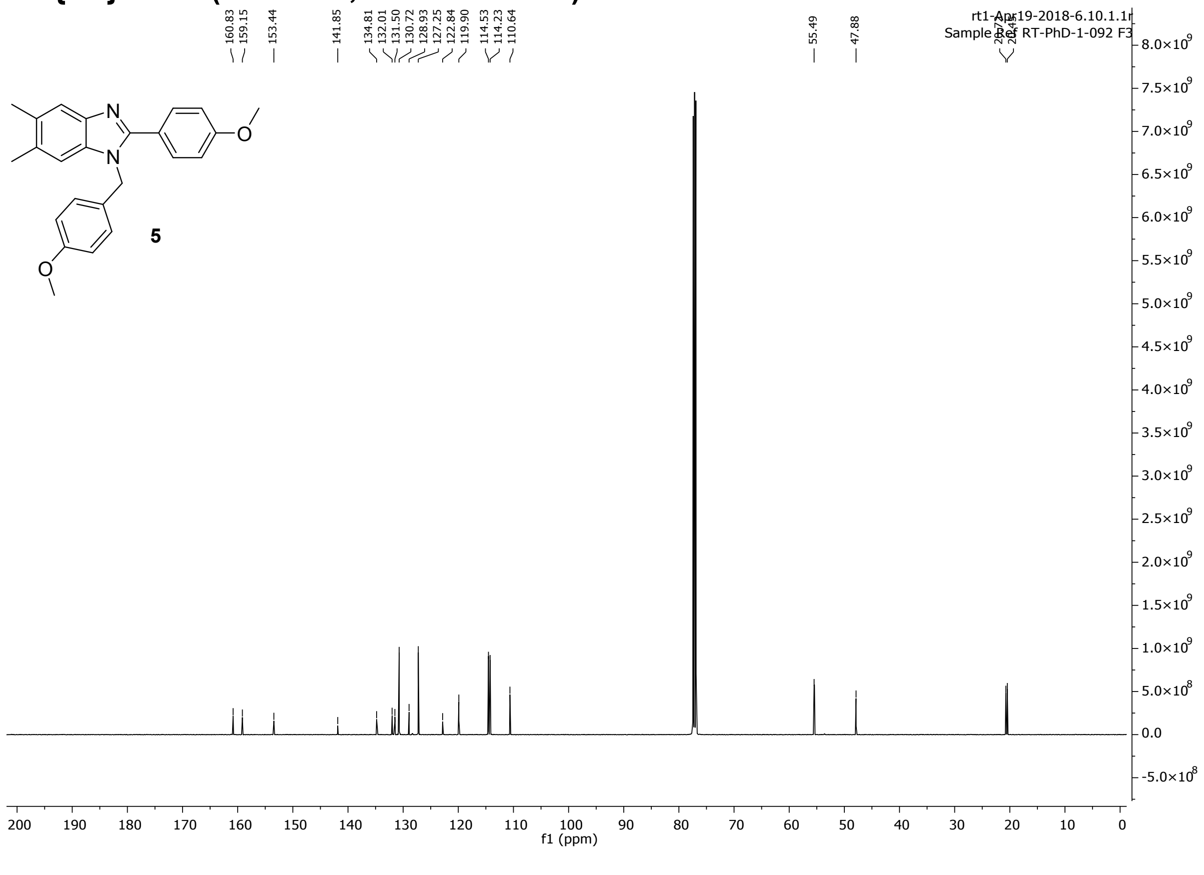

## $^{1}$H NMR (400 MHz, Chloroform-*d*)

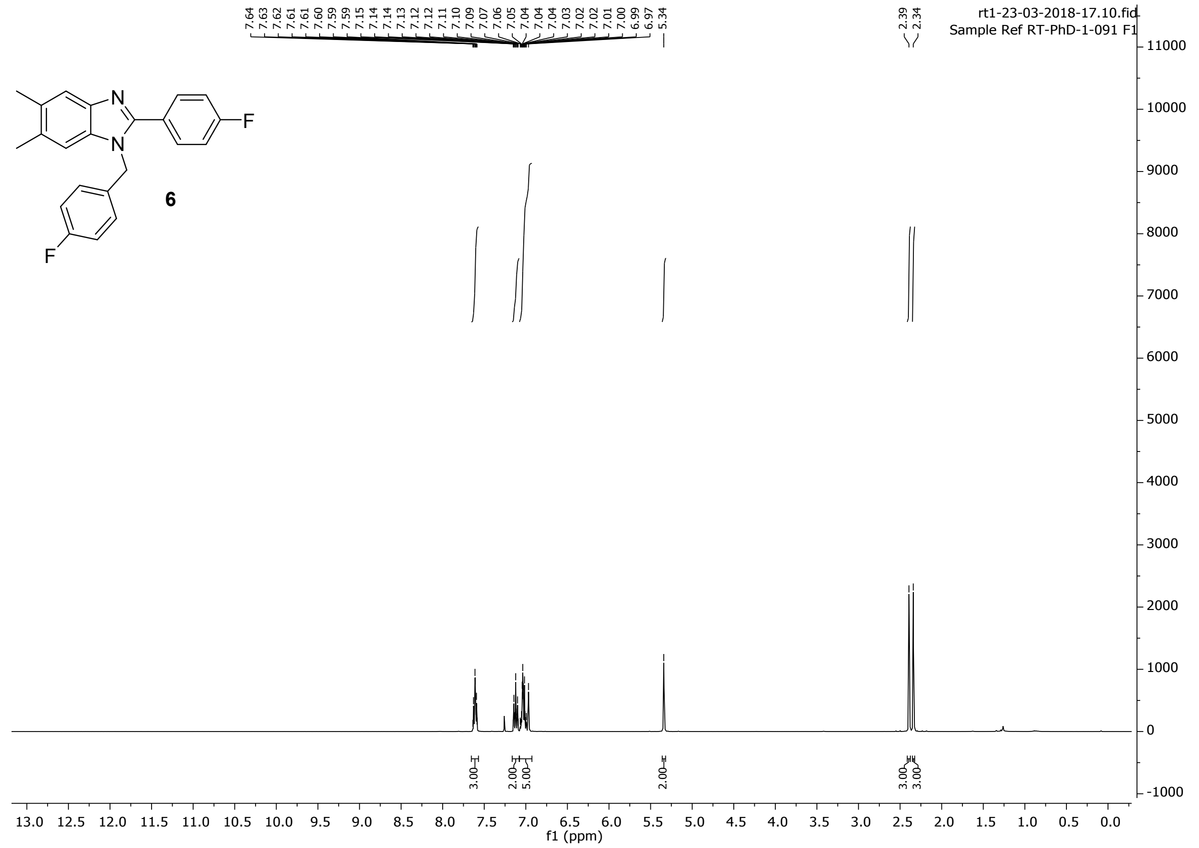

## $^{13}$C{1H} NMR (101 MHz, Chloroform-*d*)

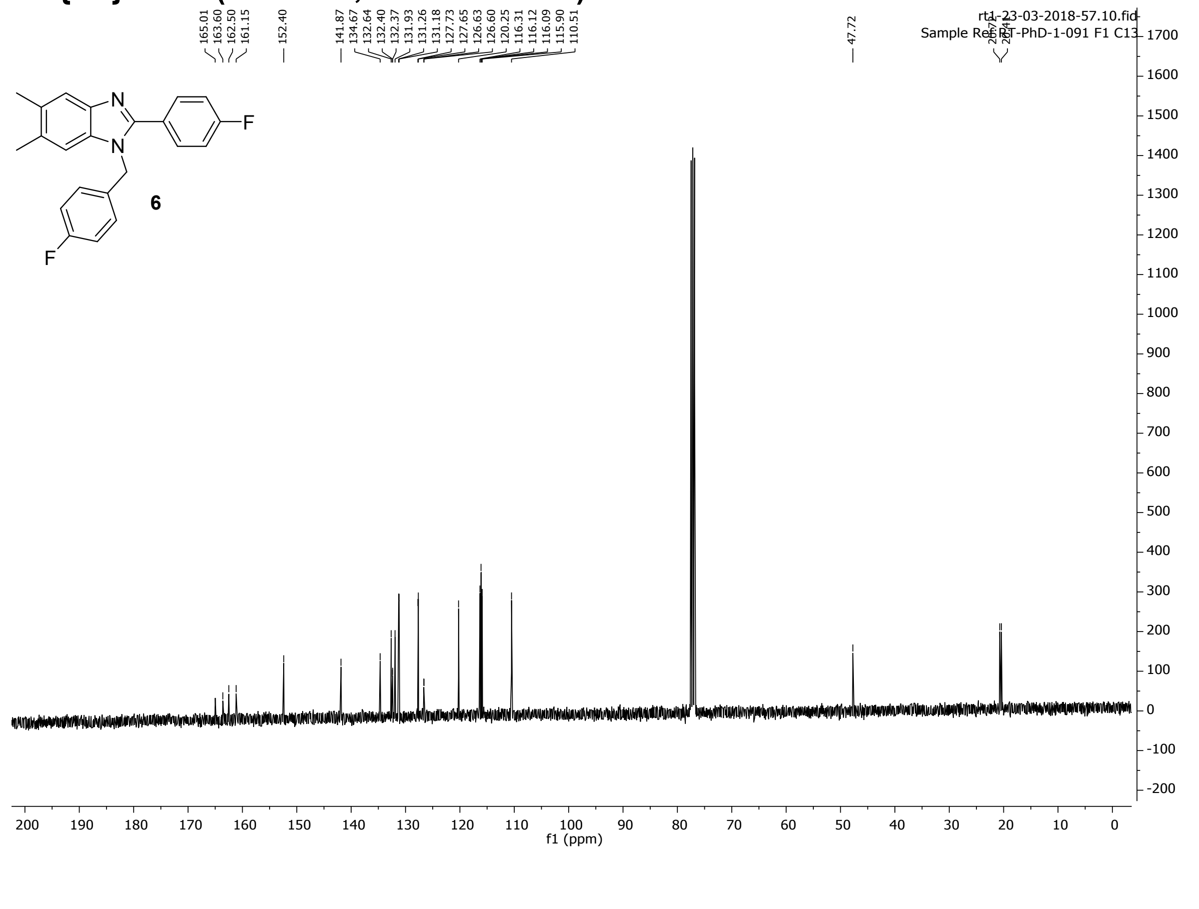

## $^{1}$H NMR (400 MHz, Chloroform-*d*)

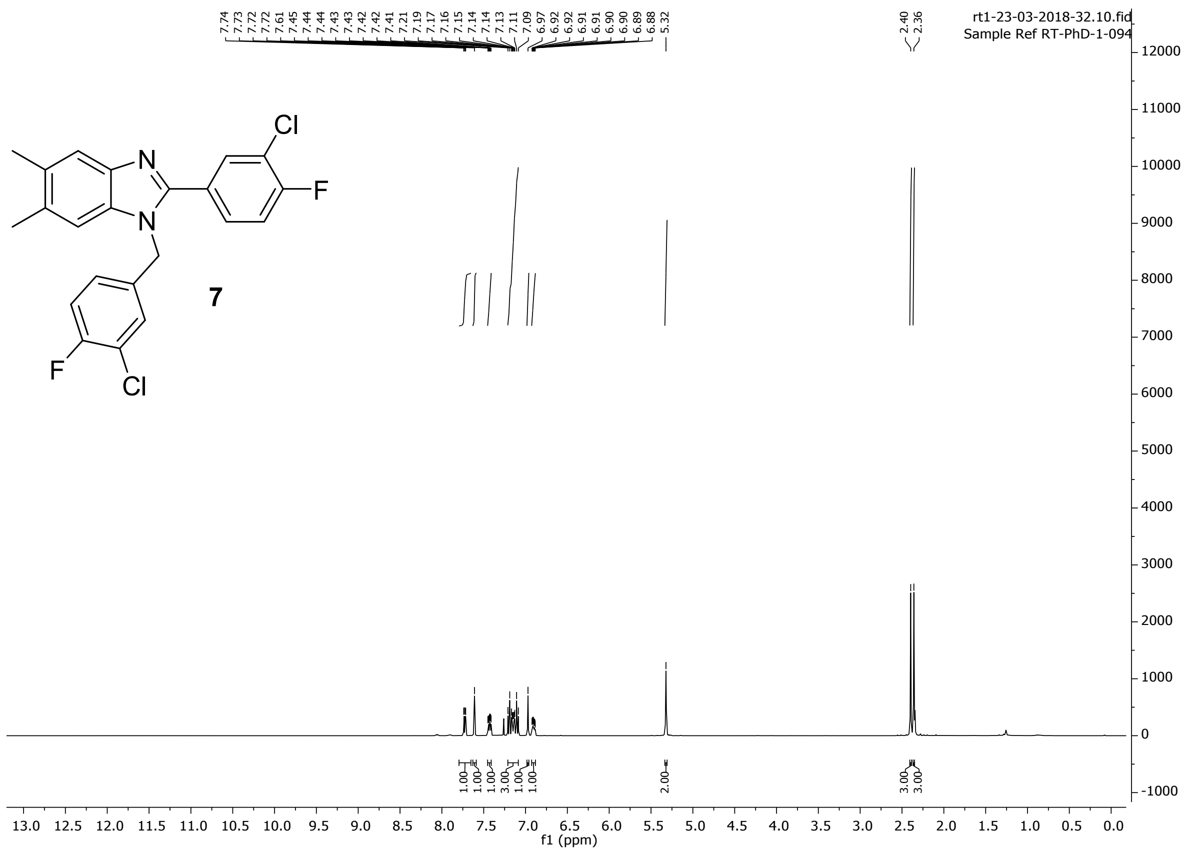


## $^{13}$C{1H} NMR (101 MHz, Chloroform-*d*)

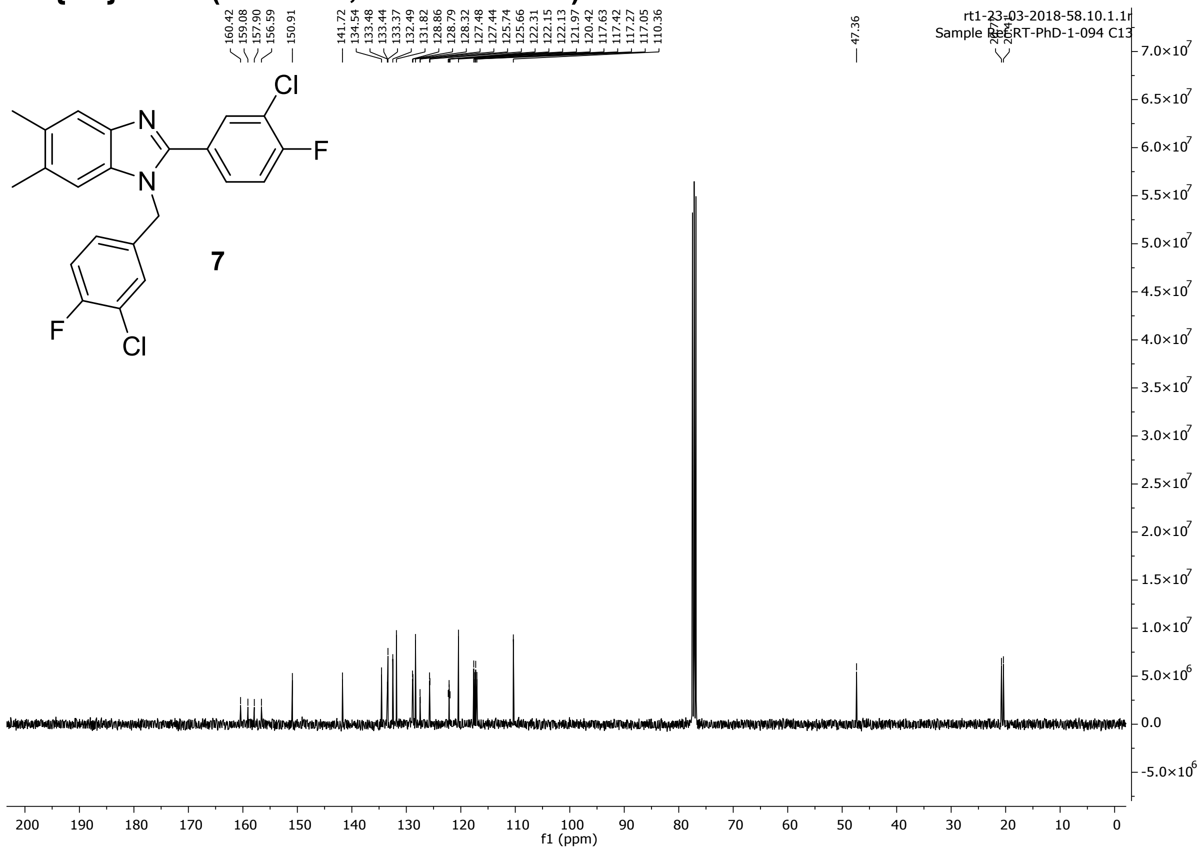

## $^1$H NMR (400 MHz, Chloroform-*d*)

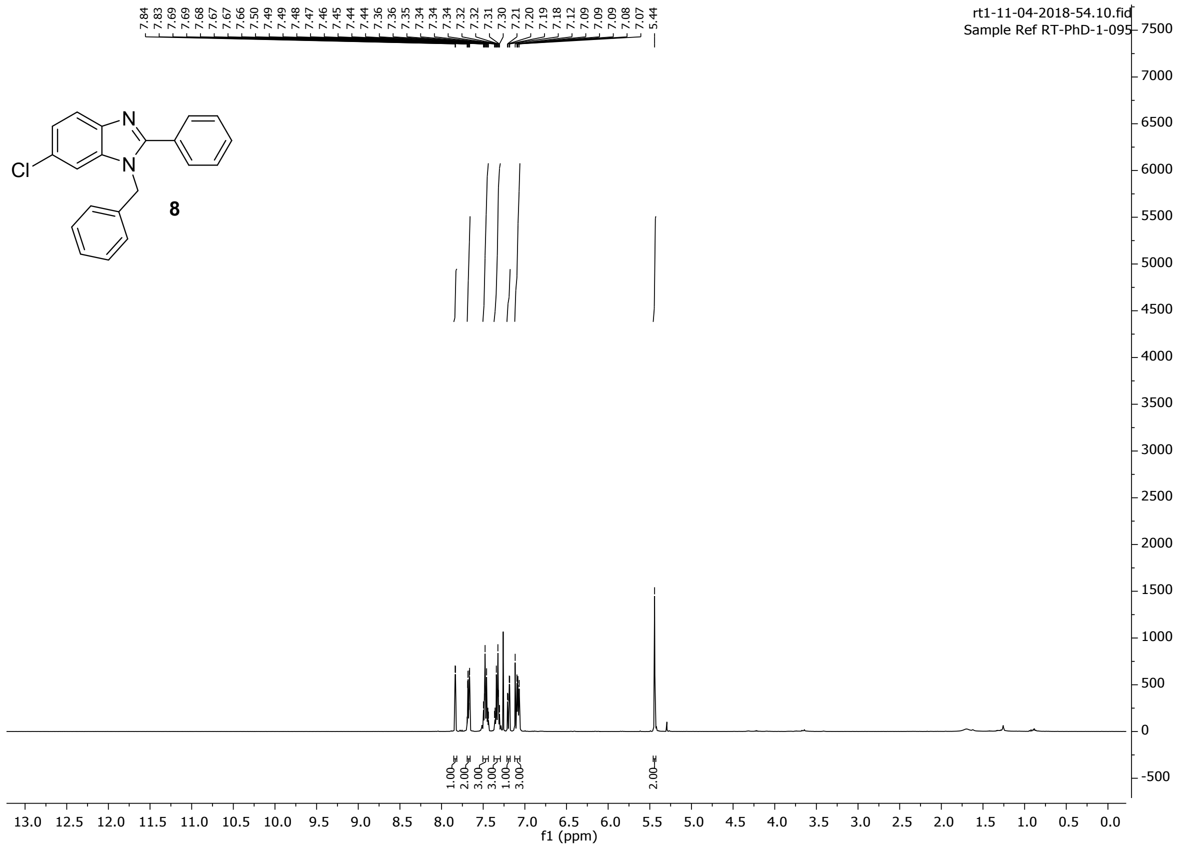


## $^{13}$C{1H} NMR (126 MHz, Chloroform-*d*)

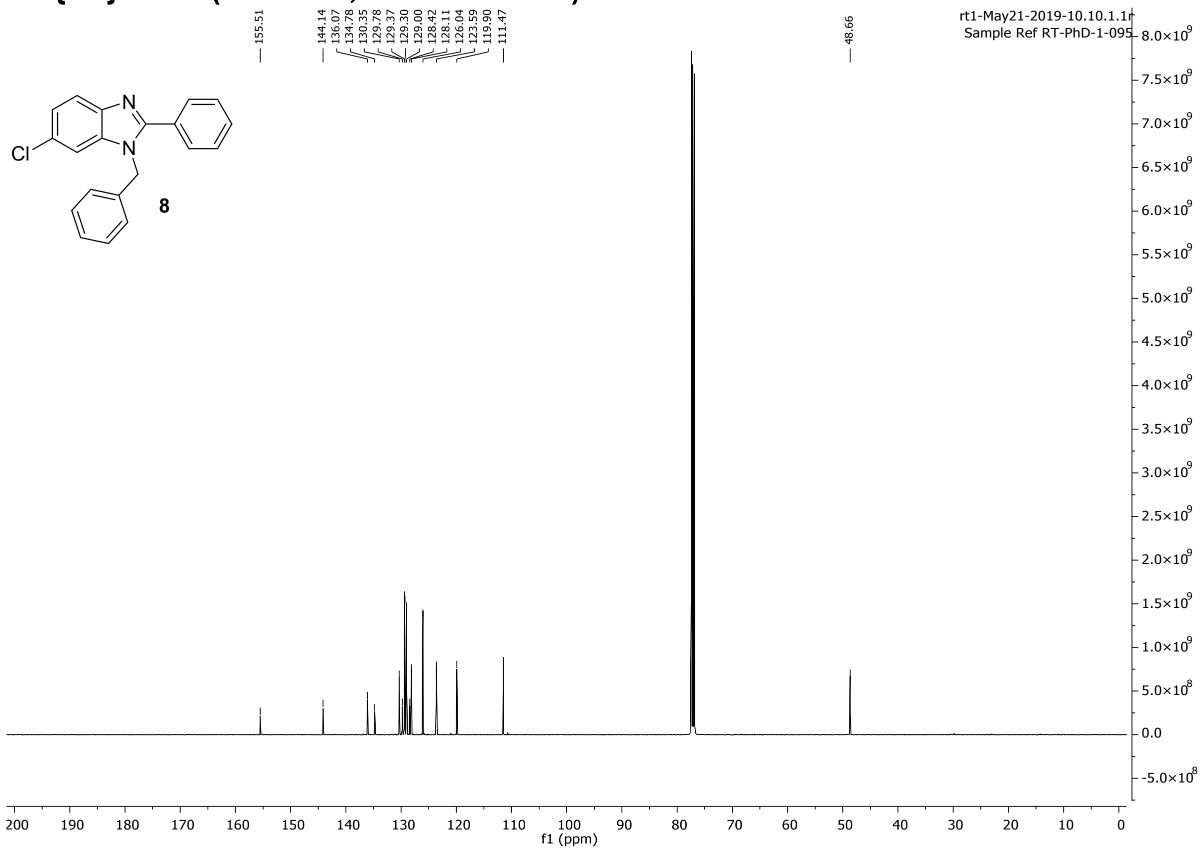

## $^{1}H$ NMR (400 MHz, Chloroform-*d*)

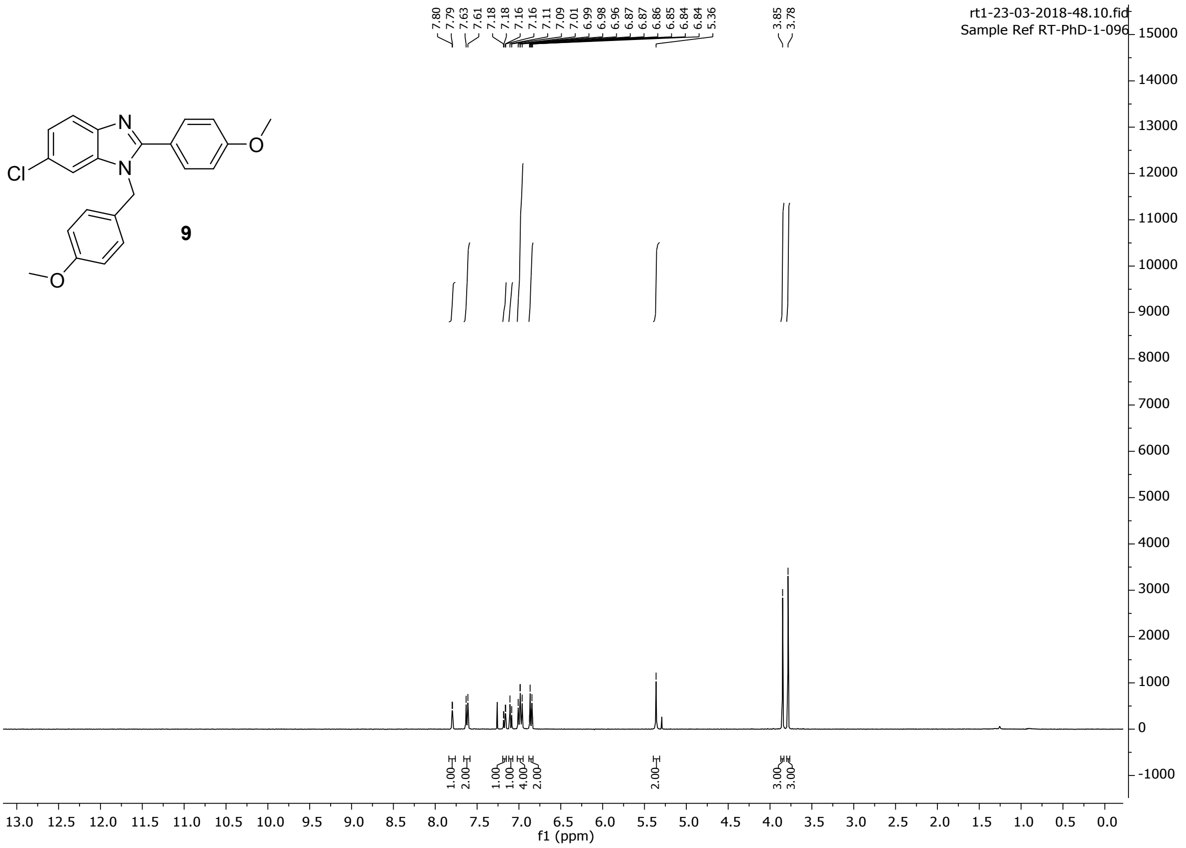


## $^{13}C\{1H\}$ NMR (101 MHz, Chloroform-*d*)

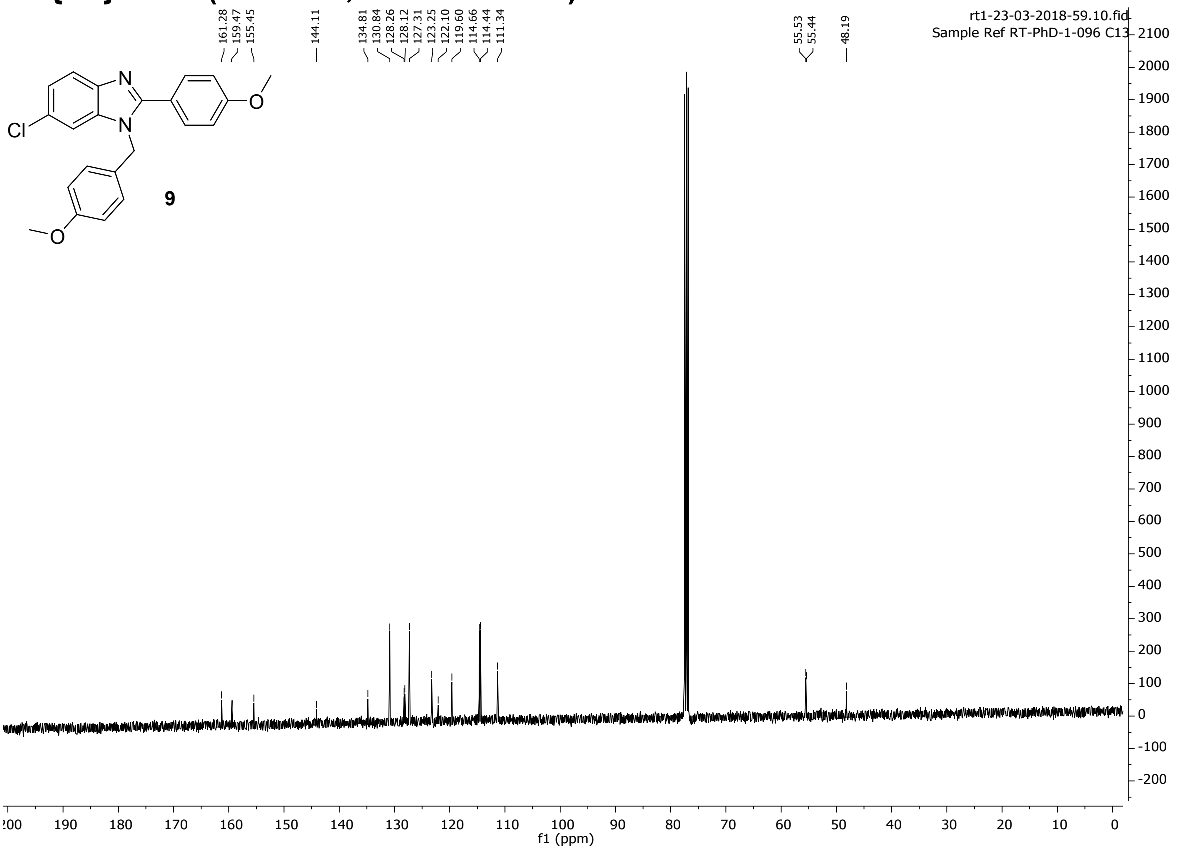

## $^{1}H$ NMR (400 MHz, Chloroform-*d*)

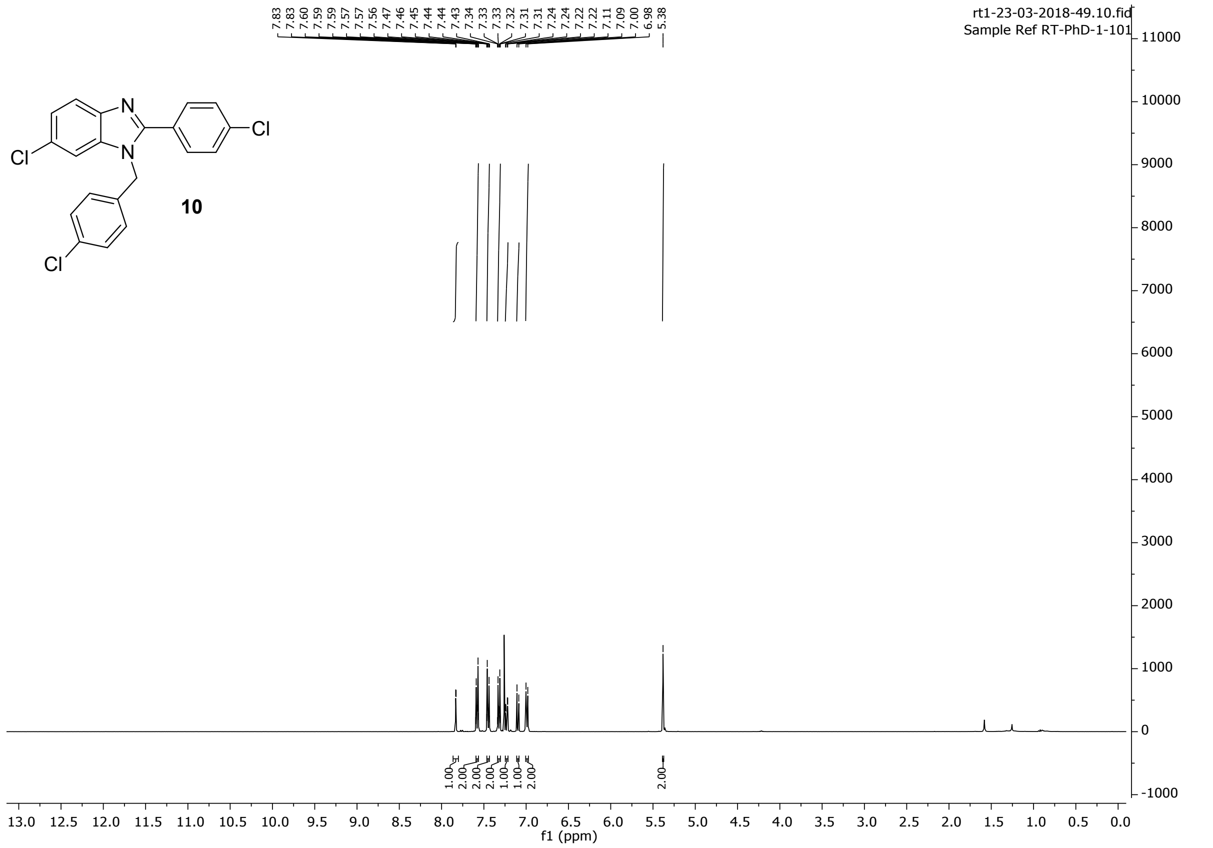


## $^{13}C\{1H\}$ NMR (126 MHz, Chloroform-*d*)

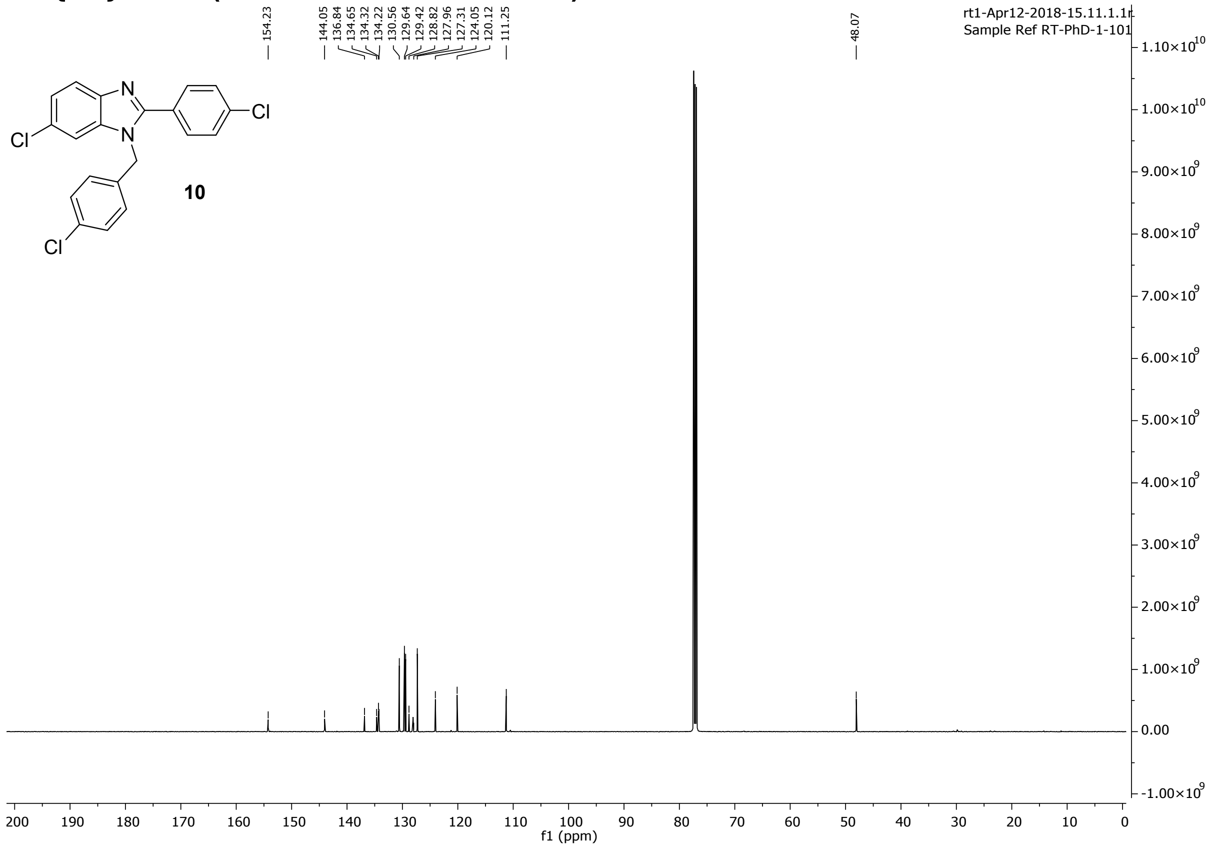

## $^1$H NMR (400 MHz, Chloroform-*d*)

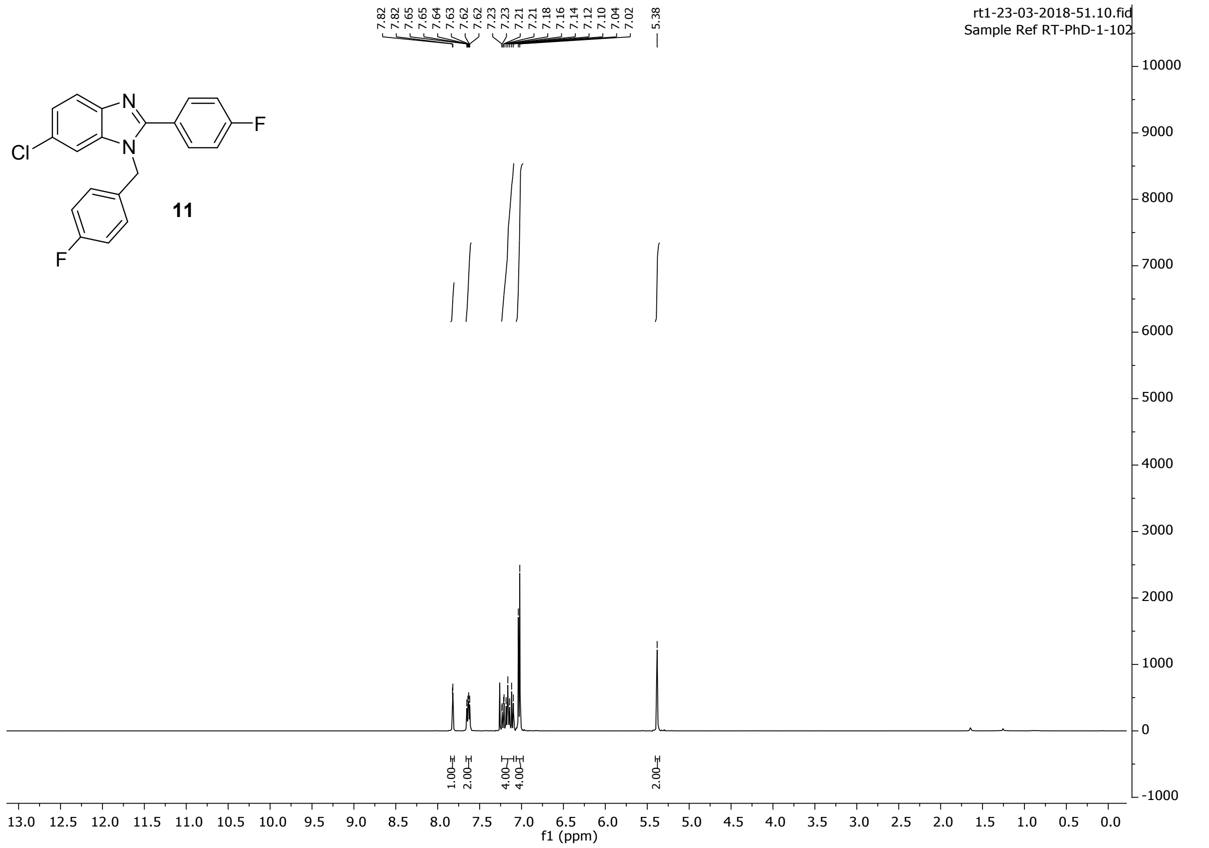


## $^{13}$C{1H} NMR (126 MHz, Chloroform-*d*)

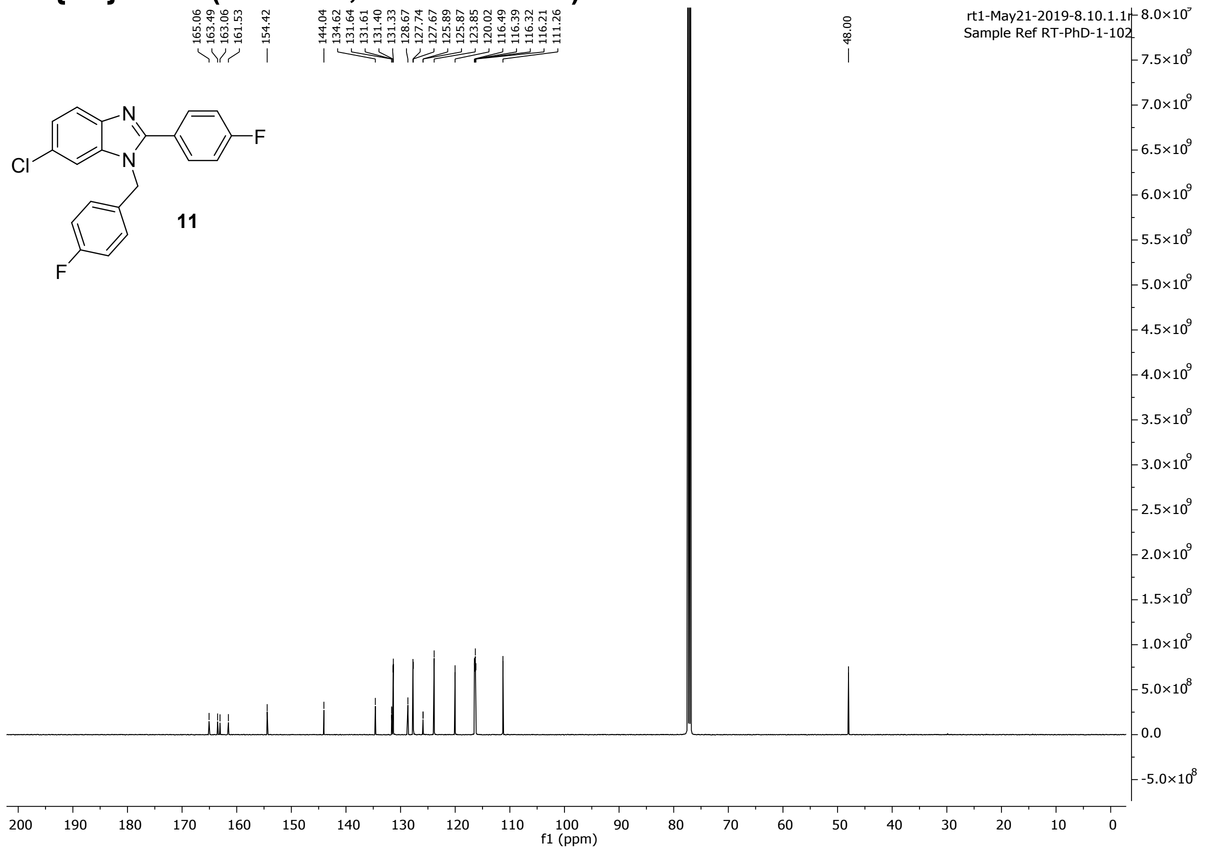

## $^1$H NMR (500 MHz, Chloroform-*d*)

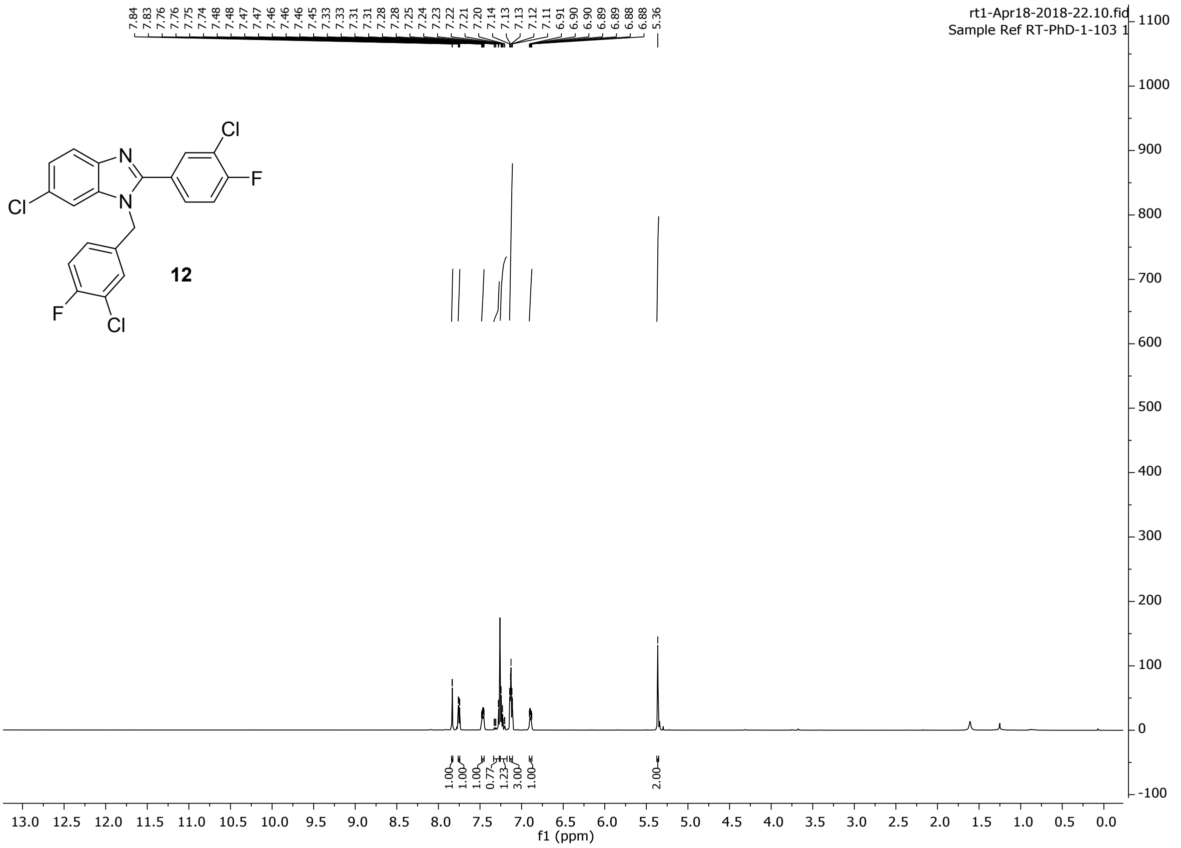


## $^{13}$C{1H} NMR (126 MHz, Chloroform-*d*)

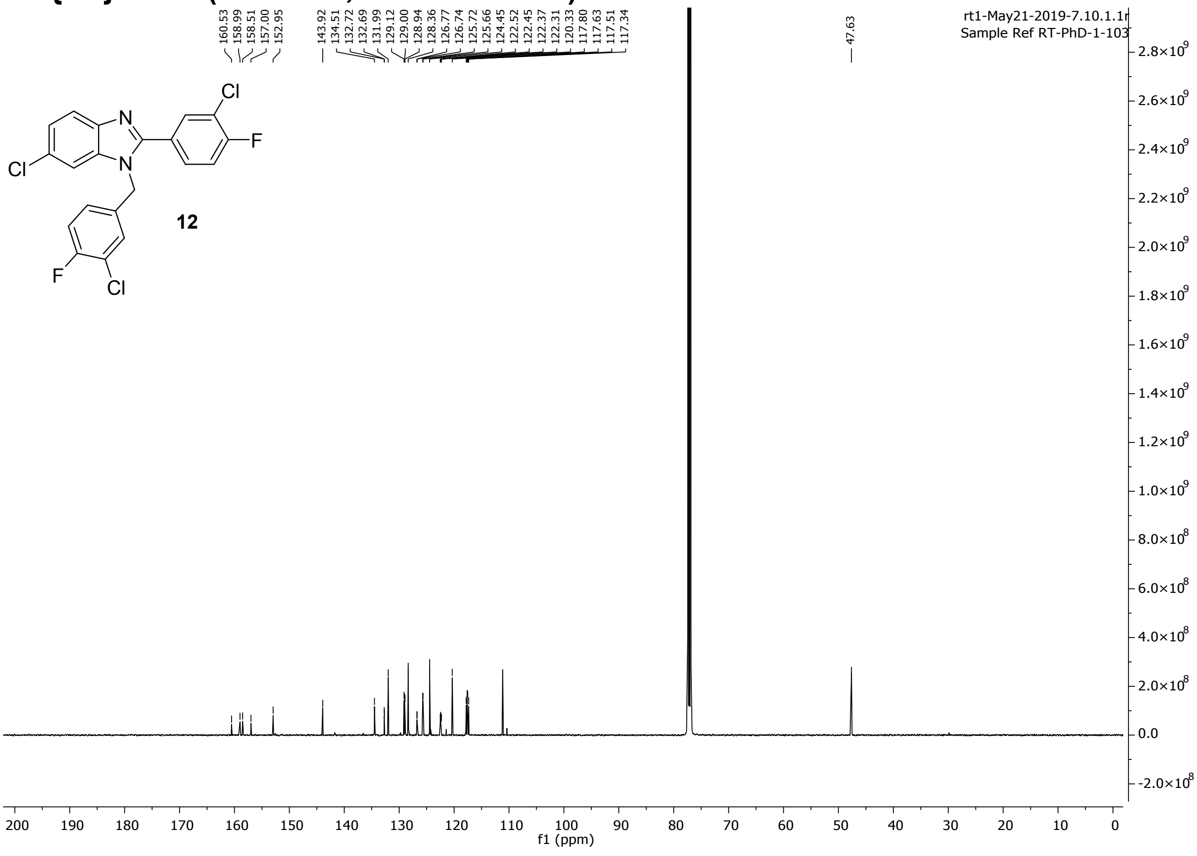

## $^{1}H$ NMR (400 MHz, Chloroform-*d*)

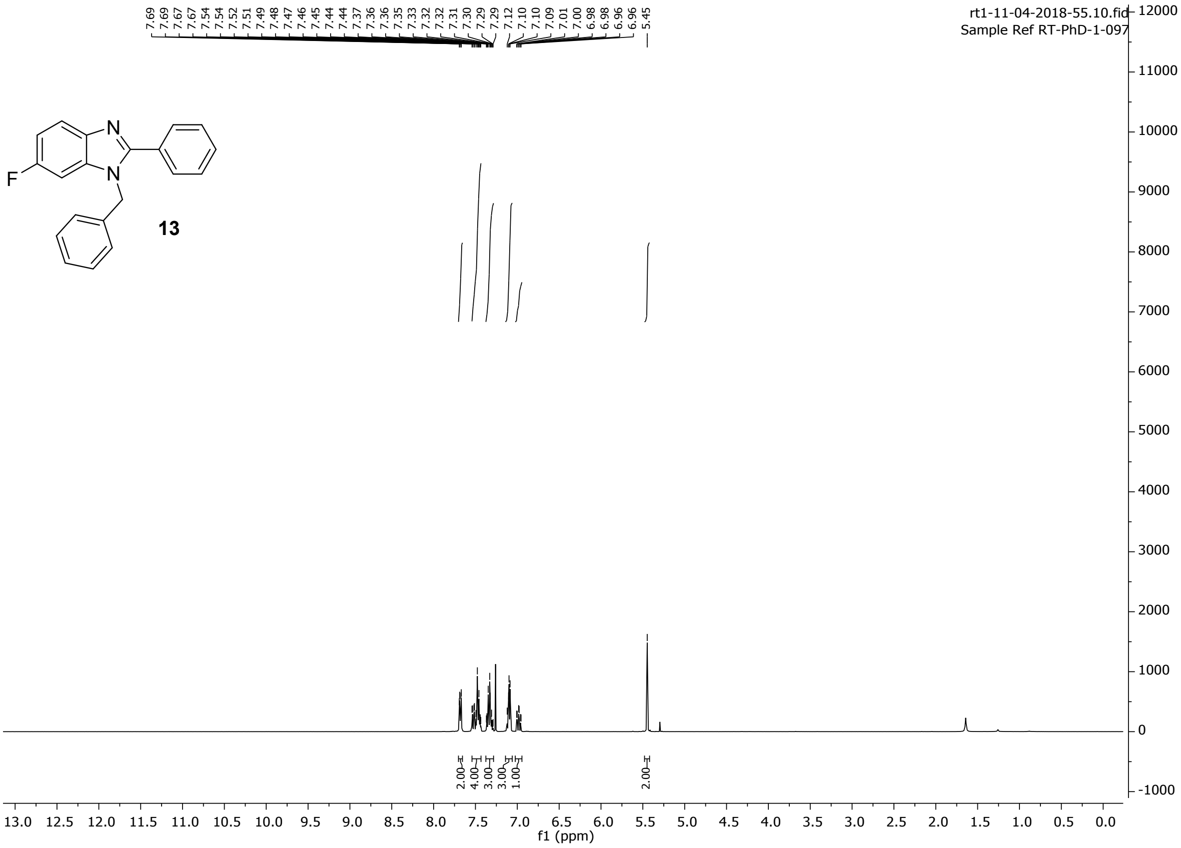


## $^{13}C\{^{1}H\}$ NMR (126 MHz, Chloroform-*d*)

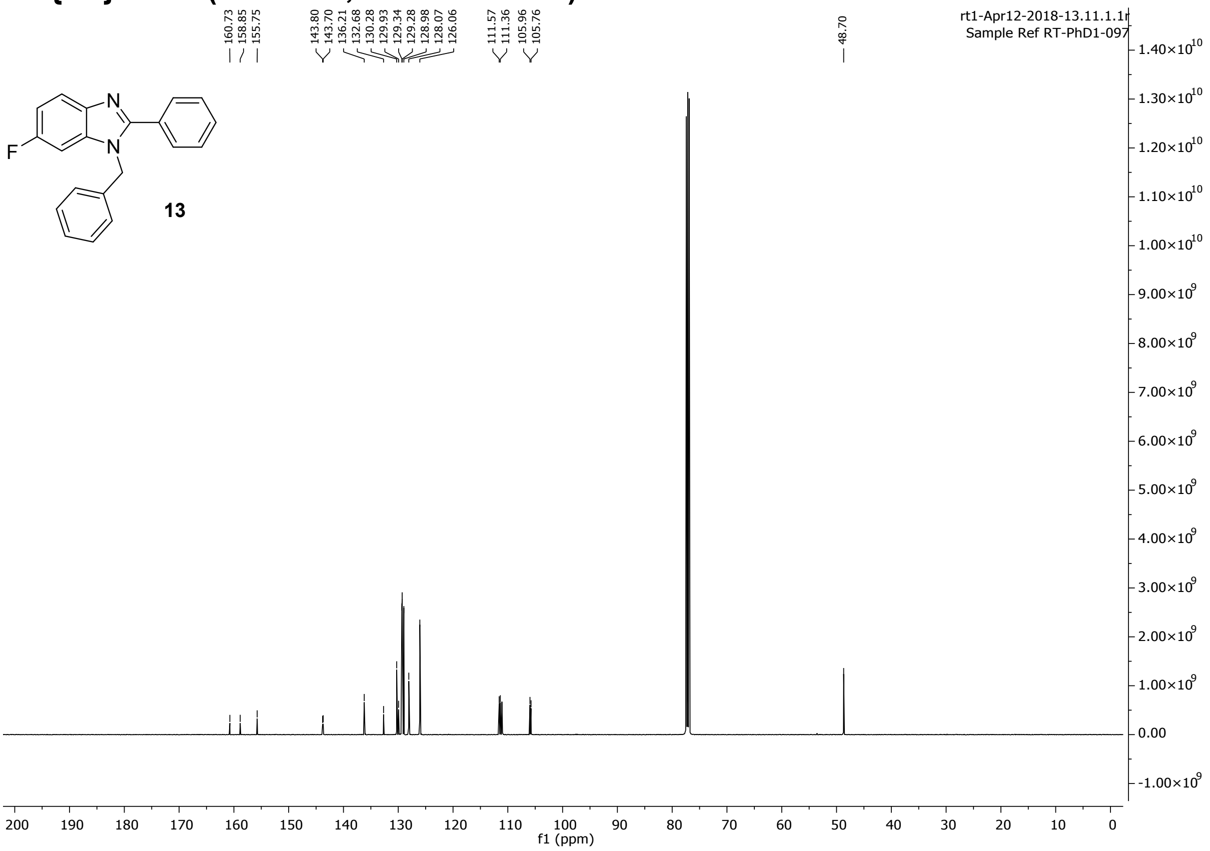

# S6.0. NMR Spectra: Benzothiazole Derivatives

## $^1$H NMR (400 MHz, Chloroform-*d*)

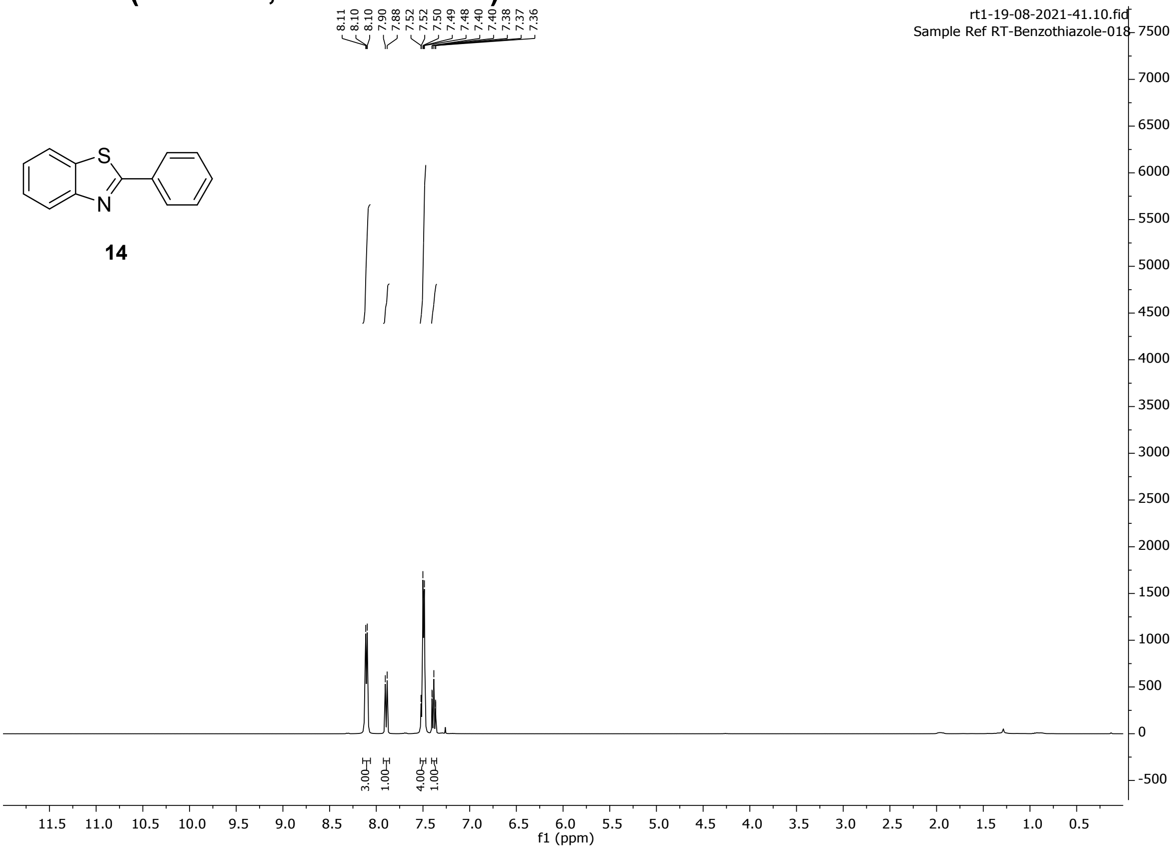


## $^{13}$C{1H} NMR (101 MHz, Chloroform-*d*)

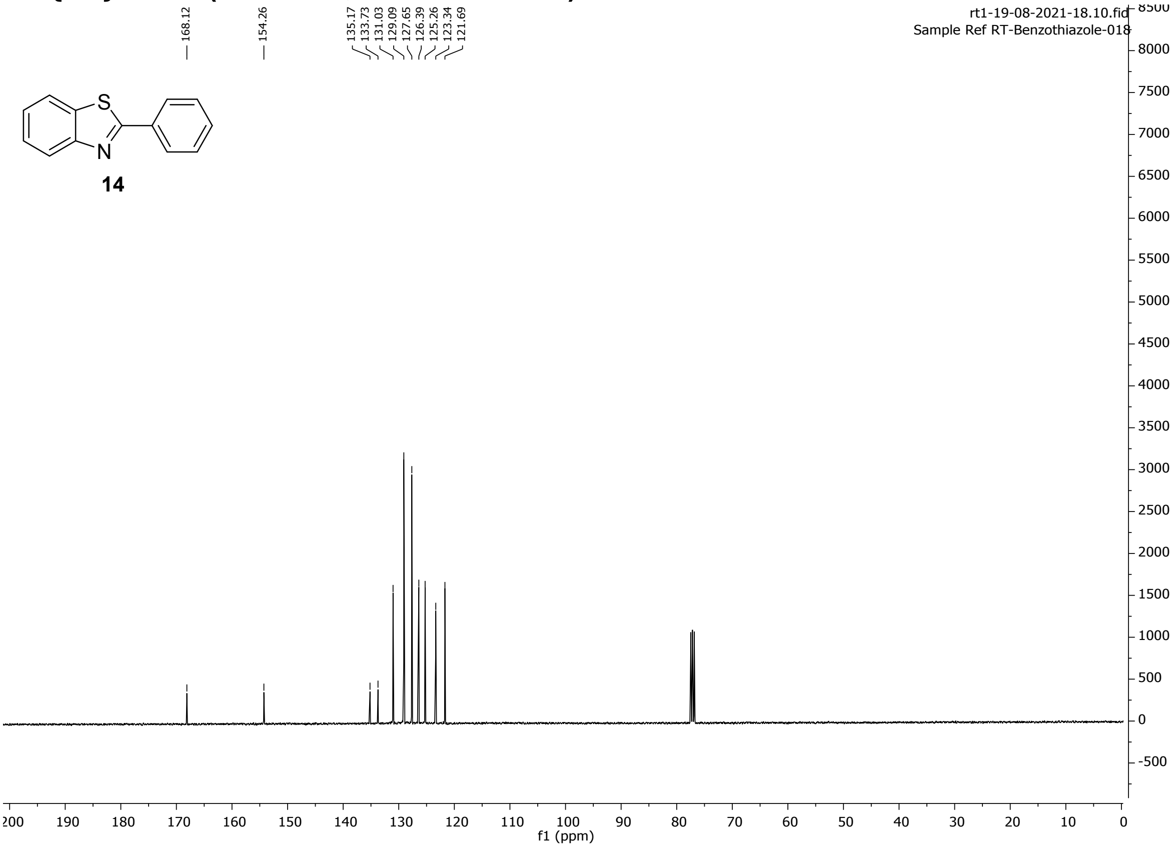

# $^{1}$H NMR (400 MHz, Chloroform-*d*)

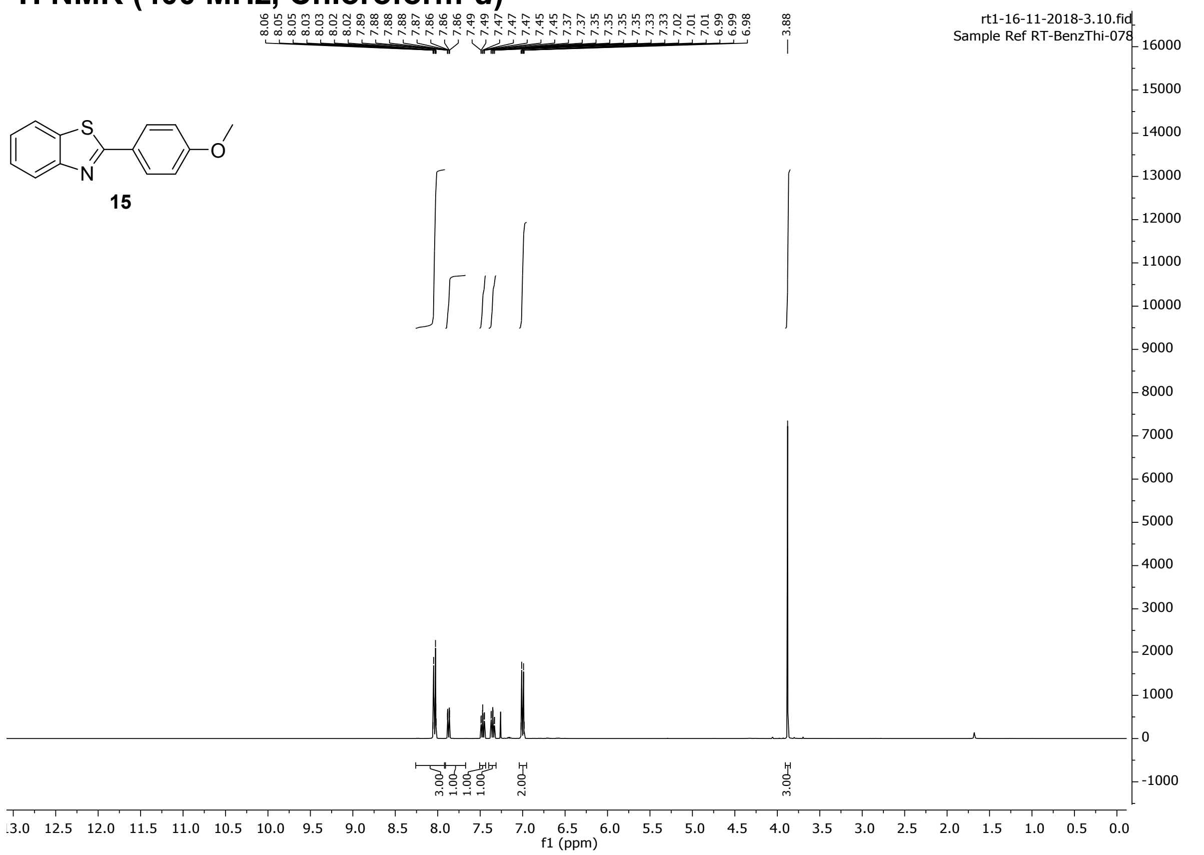


15

# $^{13}$C{1H} NMR (101 MHz, Chloroform-*d*)

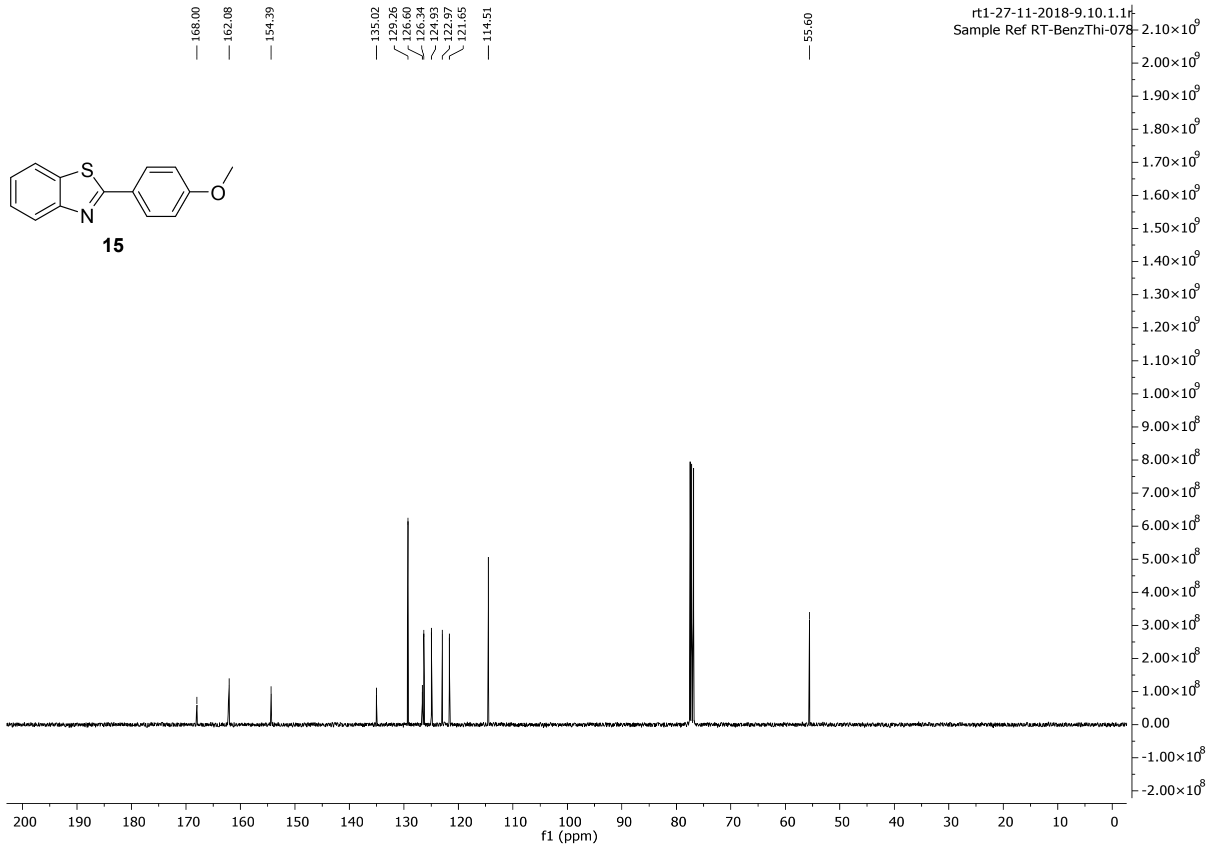


15

## $^1H$ NMR (400 MHz, DMSO-$d_6$)

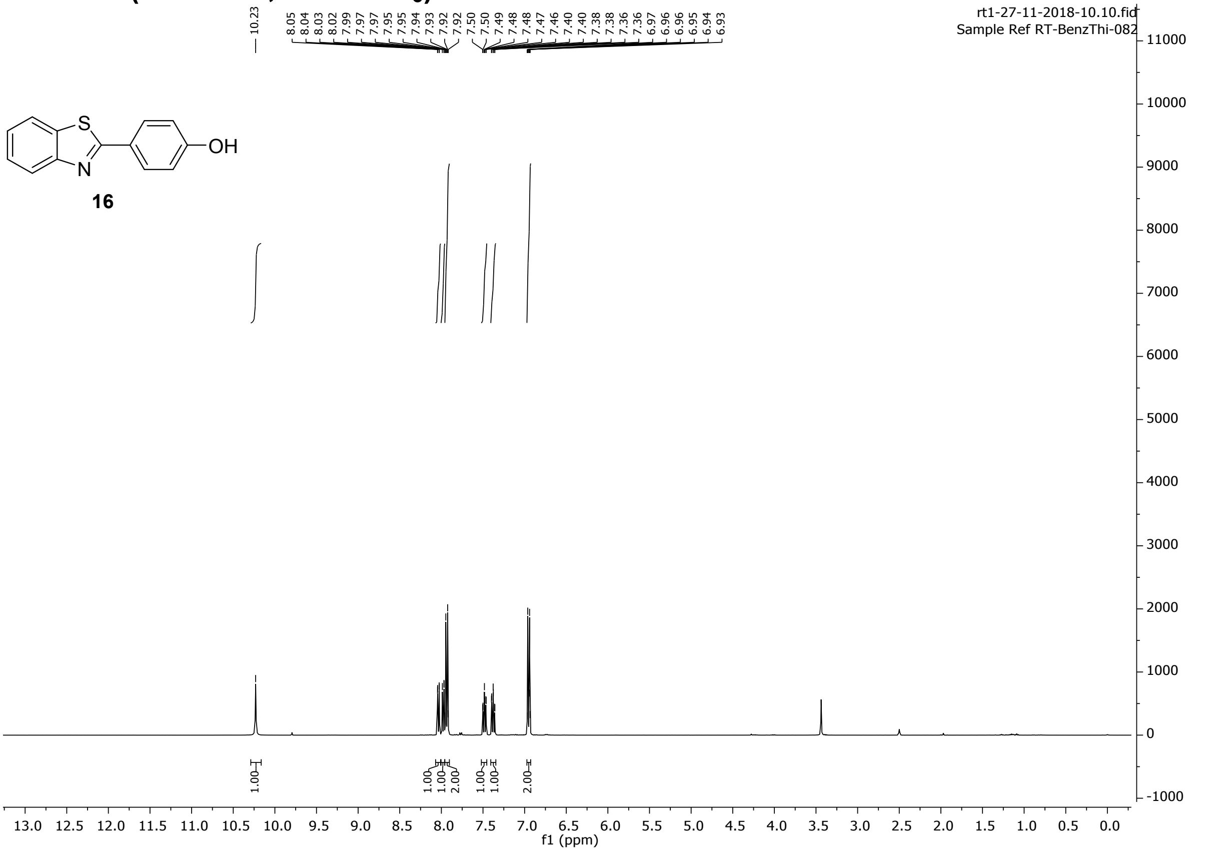

16

## $^{13}C\{1H\}$ NMR (101 MHz, DMSO-$d_6$)

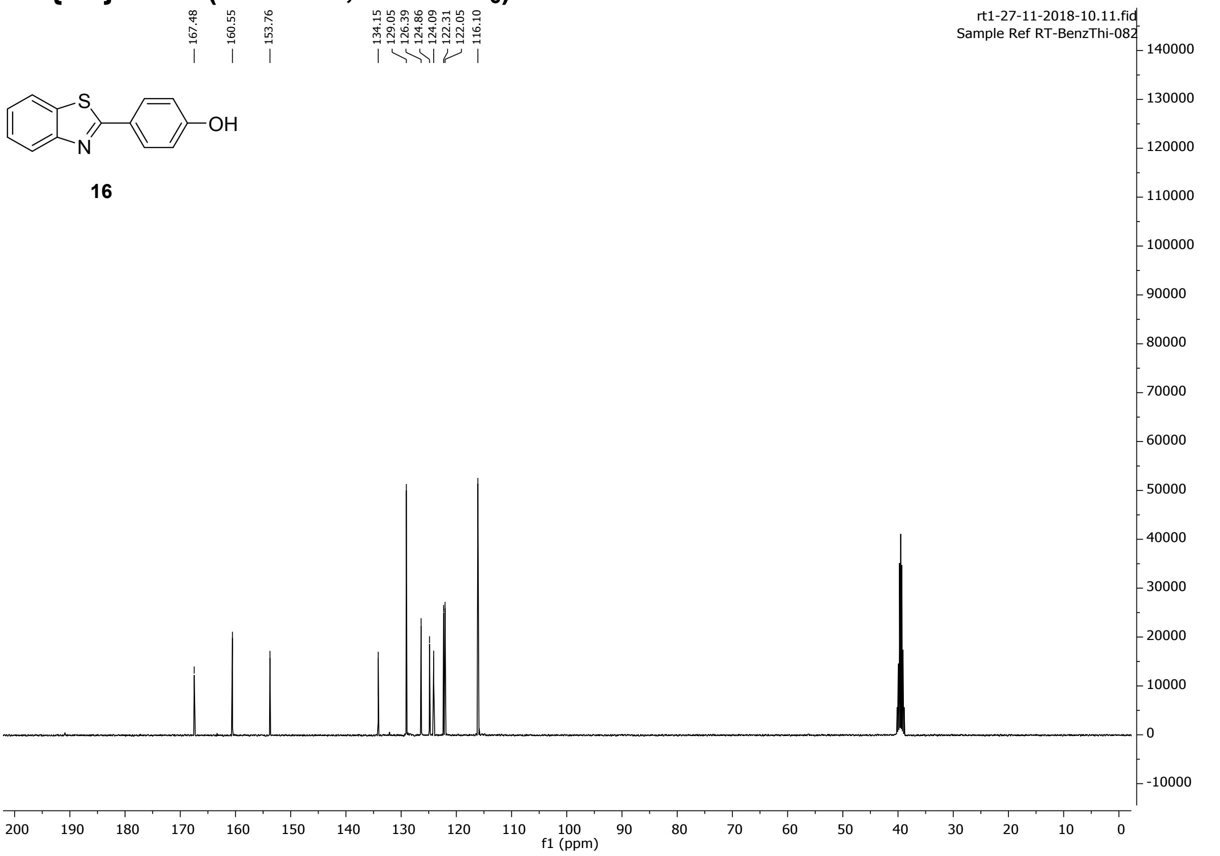

16

## $^{1}H$ NMR (400 MHz, Chloroform-*d*)

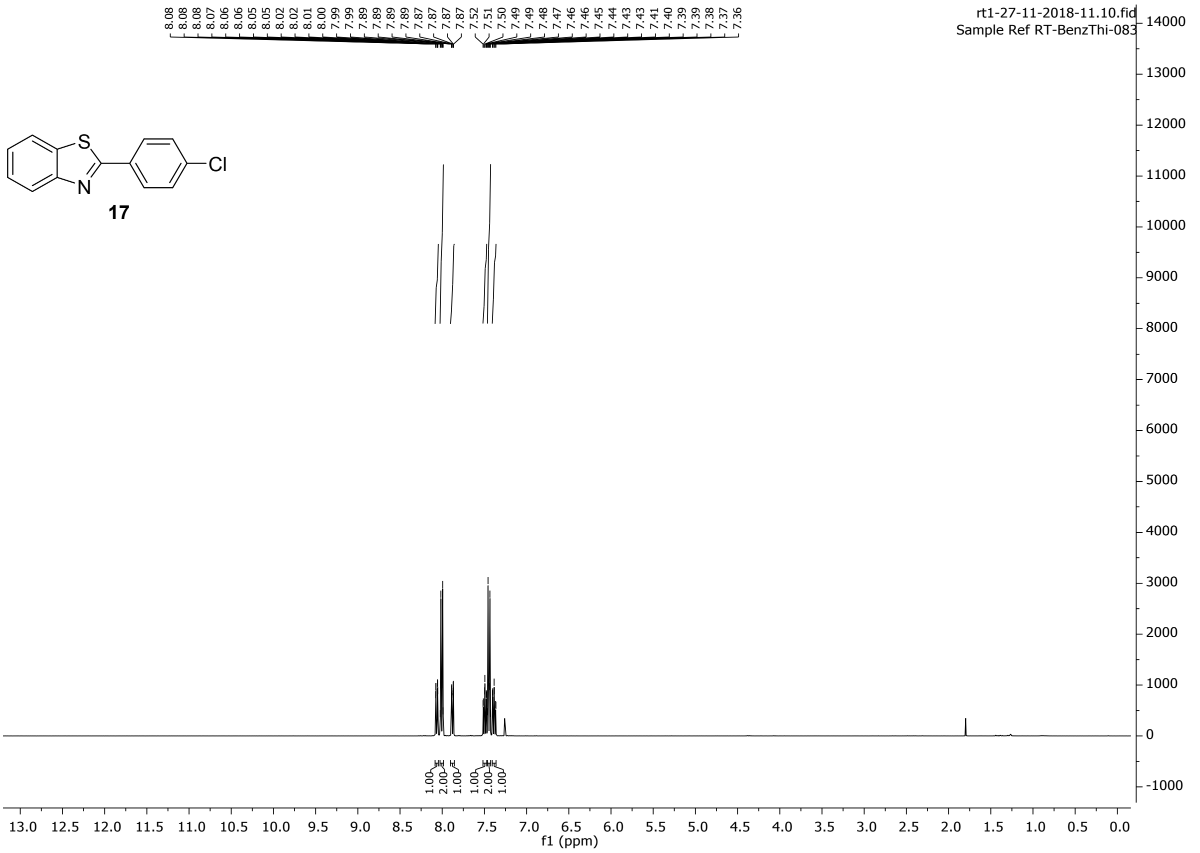


## $^{13}C\{1H\}$ NMR (101 MHz, Chloroform-*d*)

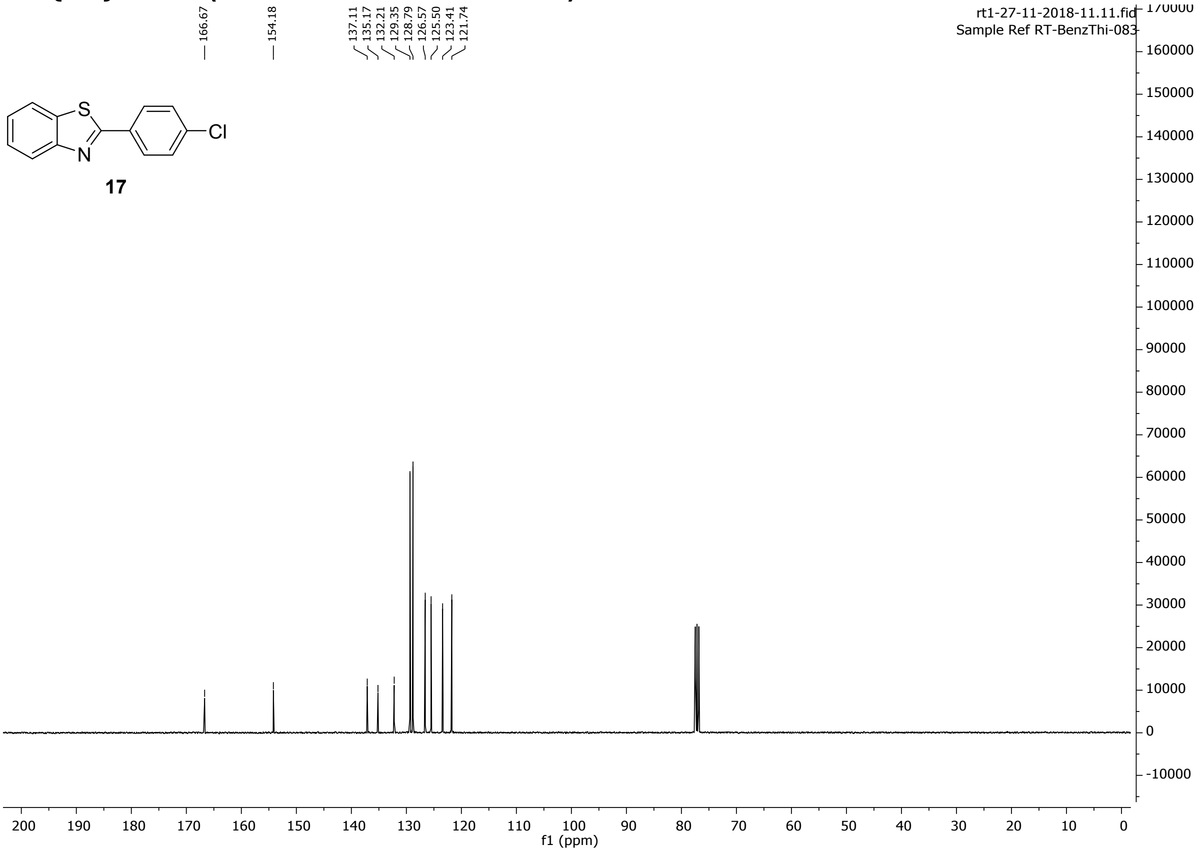

# $^1$H NMR (400 MHz, Chloroform-*d*)

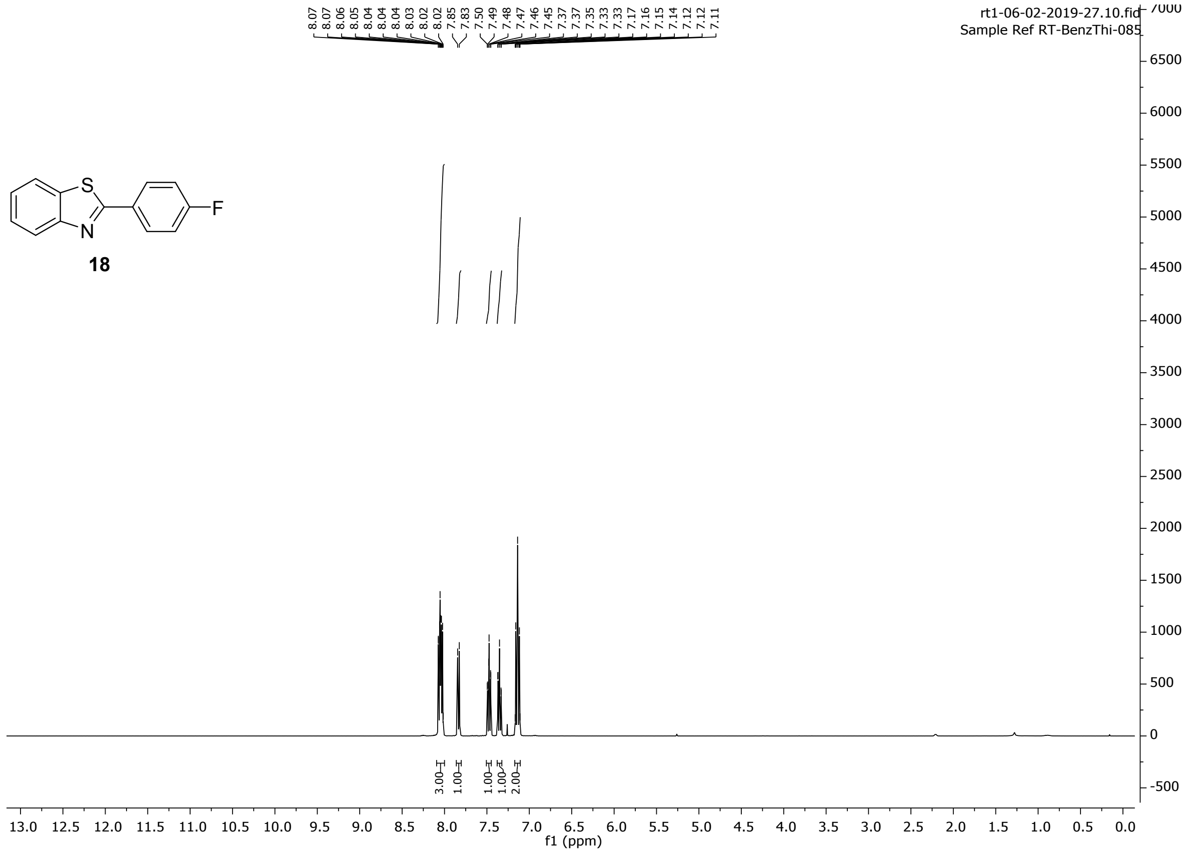


# $^{13}$C{1H} NMR (101 MHz, Chloroform-*d*)

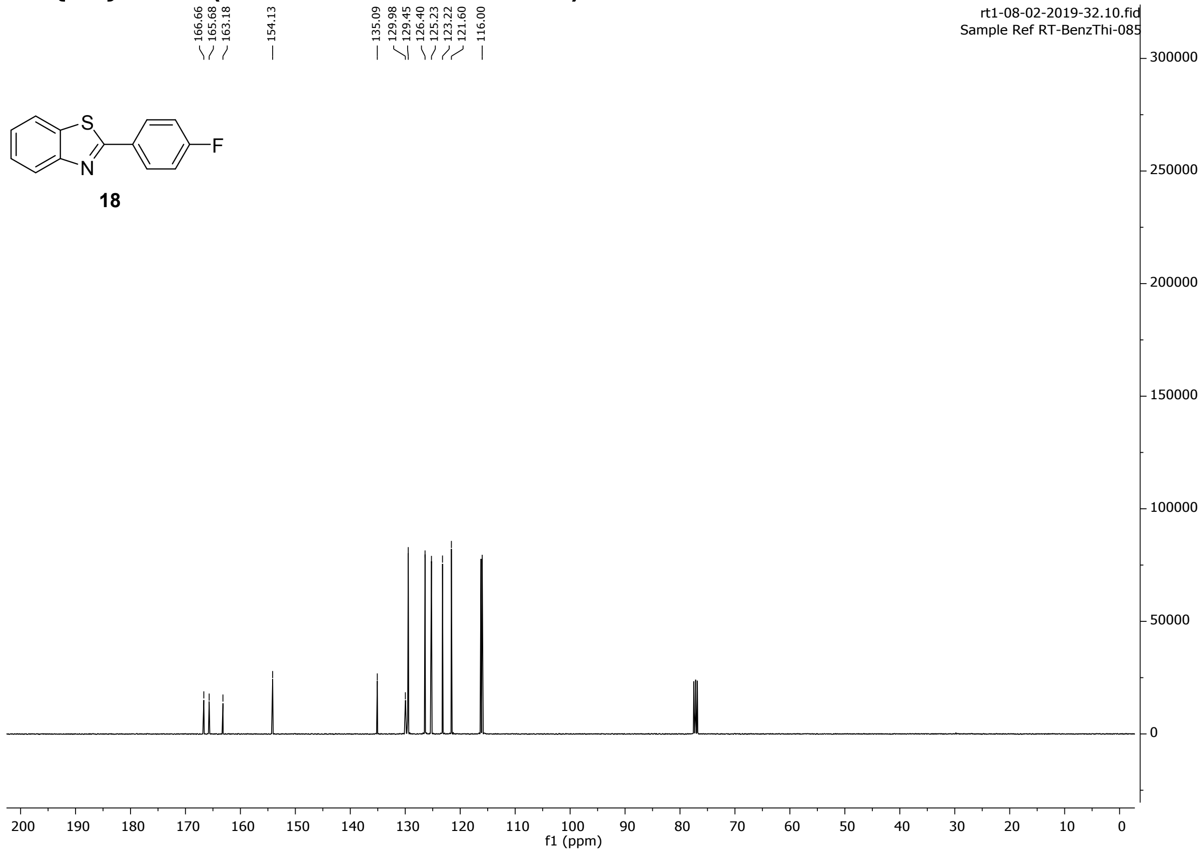

## $^1$H NMR (400 MHz, Chloroform-*d*)

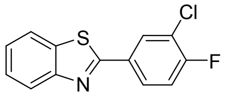

19

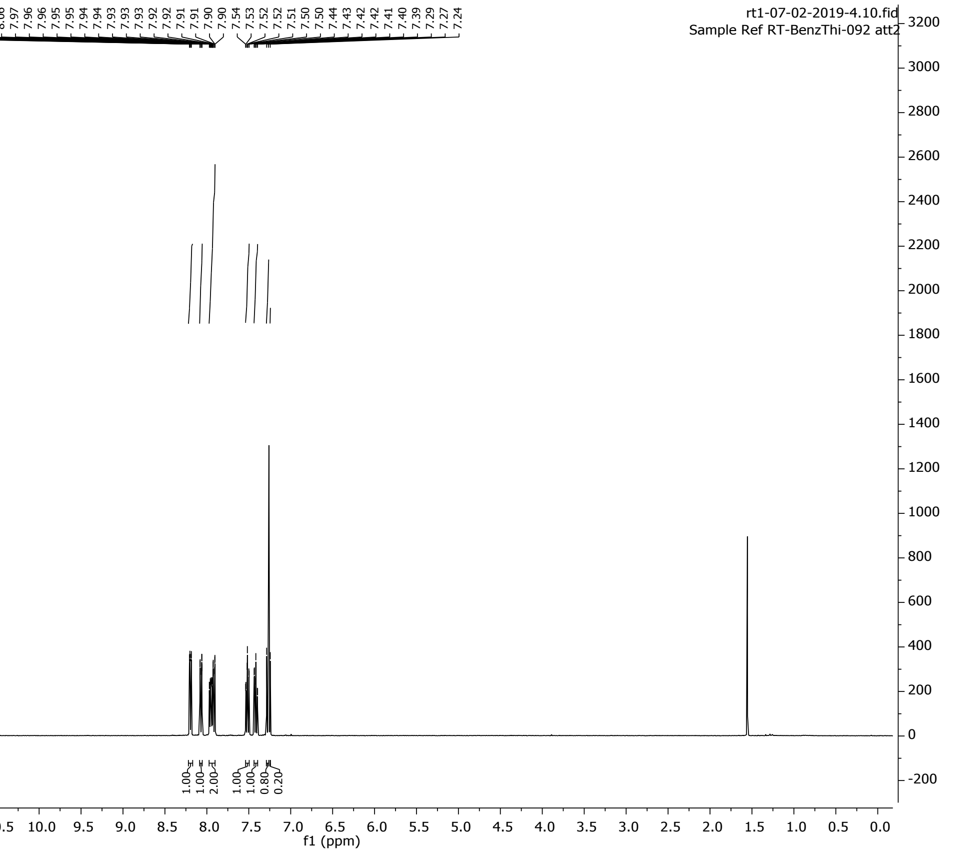


## $^{13}$C{1H} NMR (126 MHz, Chloroform-*d*)

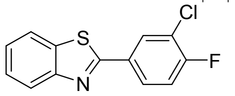

19

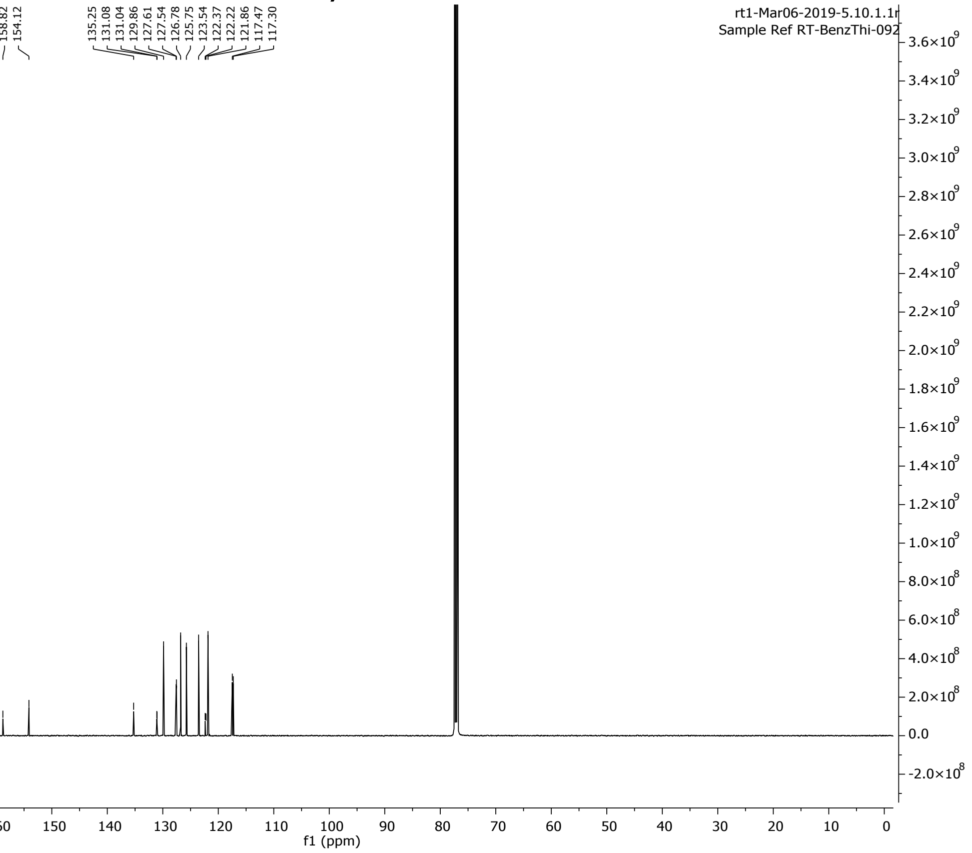

## $^{1}$H NMR (500 MHz, Chloroform-*d*)

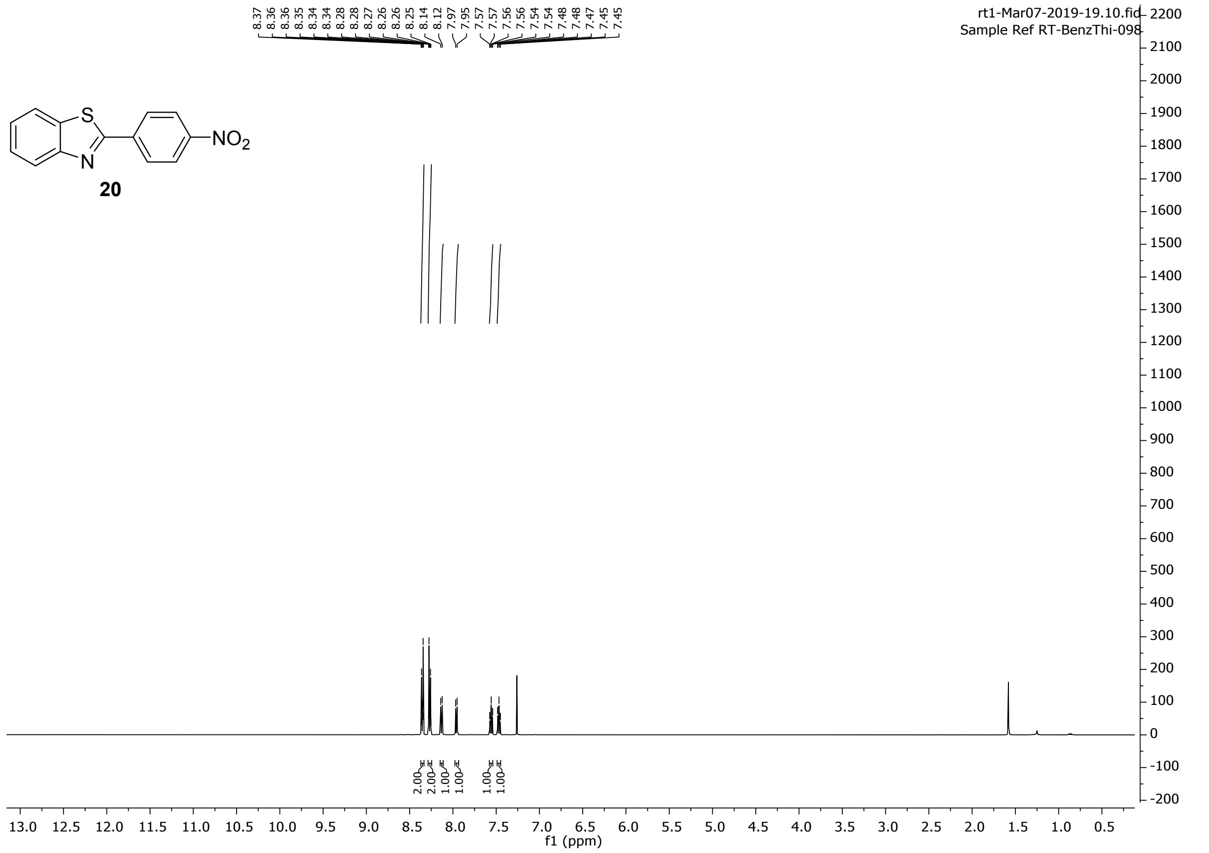


## $^{13}$C{1H} NMR (126 MHz, Chloroform-*d*)

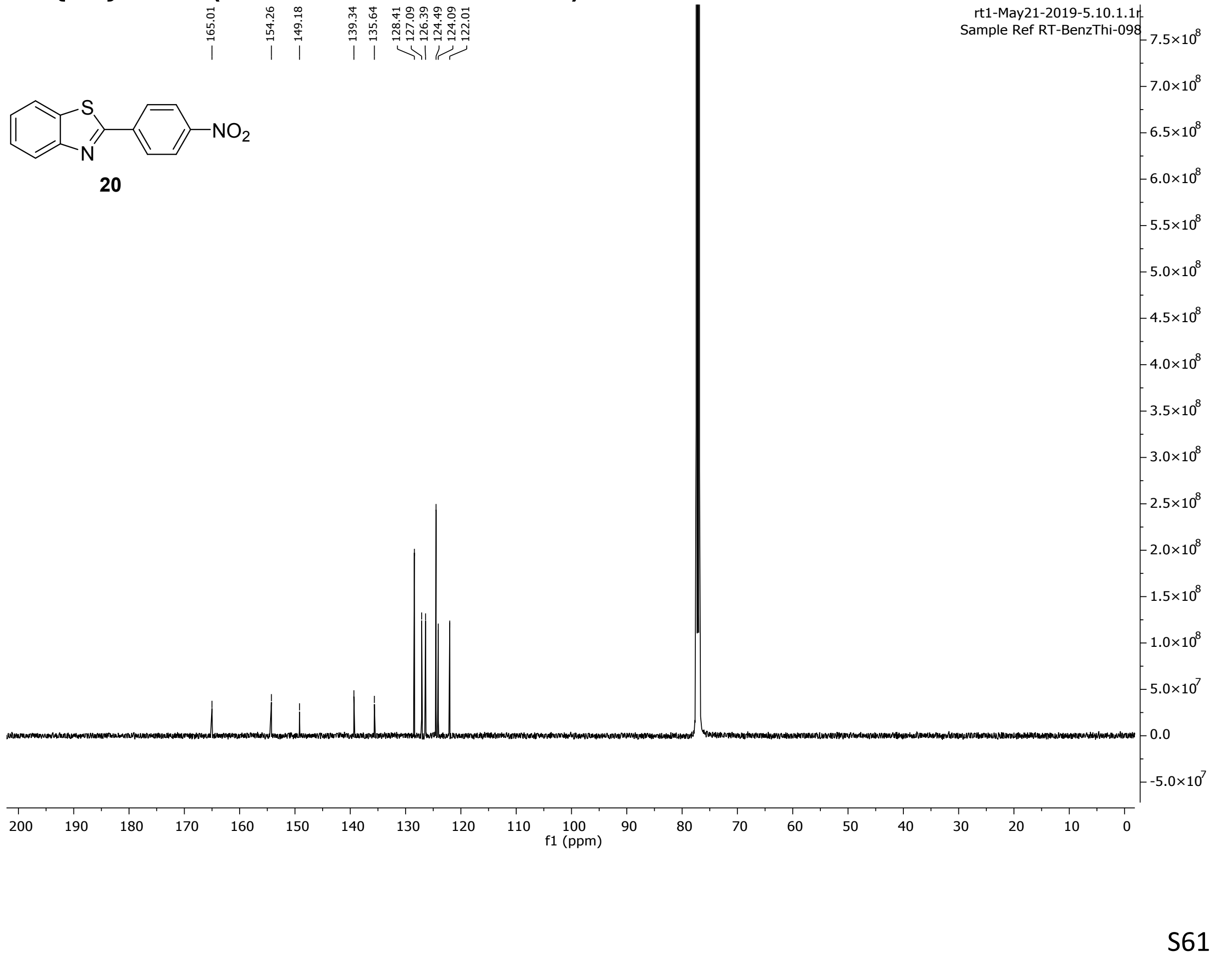

## $^{1}H$ NMR (400 MHz, Chloroform-*d*)

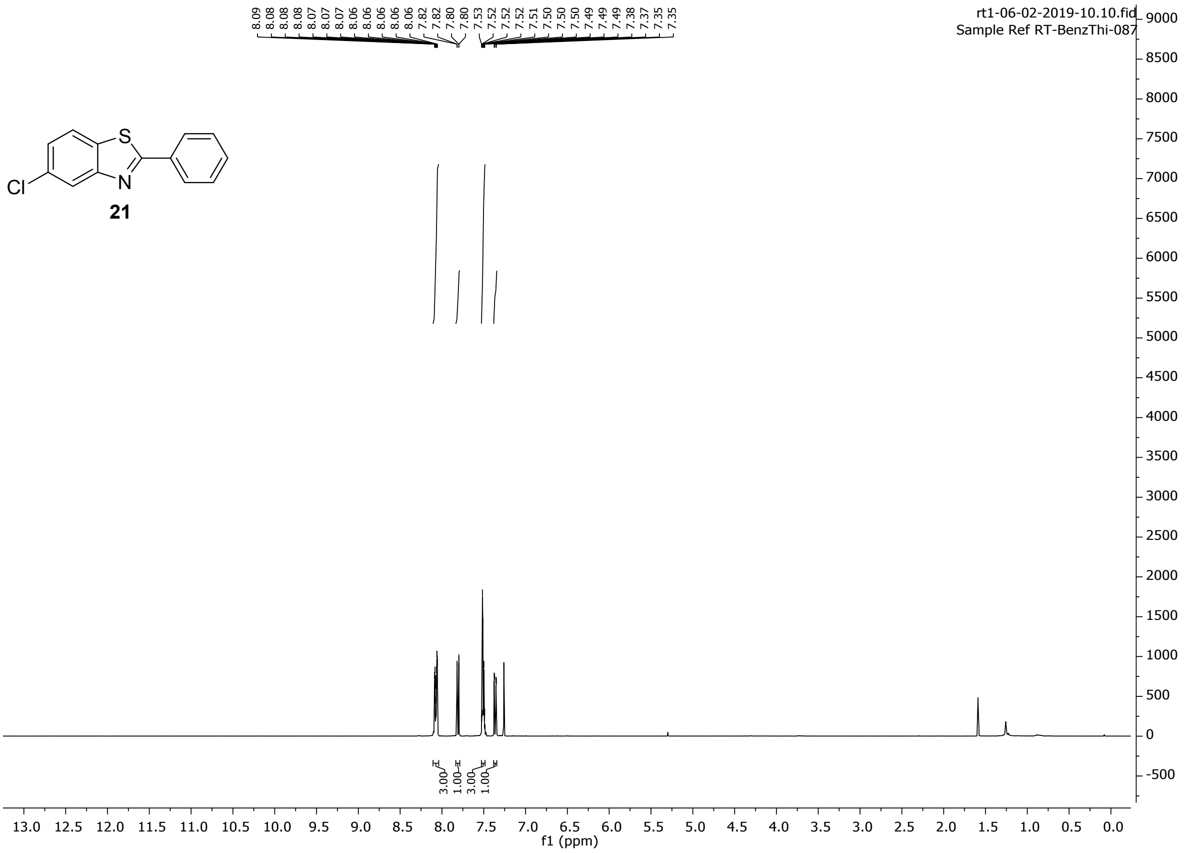


## $^{13}C\{1H\}$ NMR (101 MHz, Chloroform-*d*)

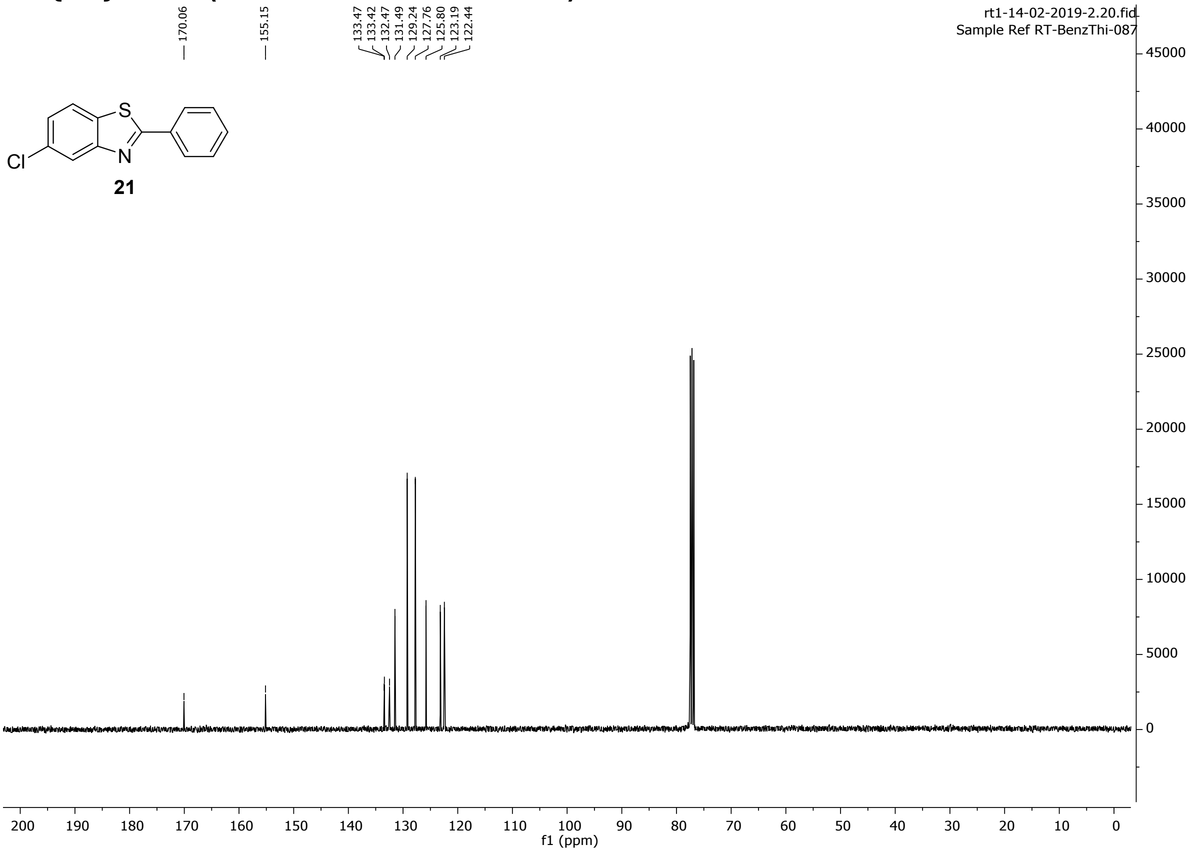

## $^{1}H$ NMR (400 MHz, Chloroform-*d*)

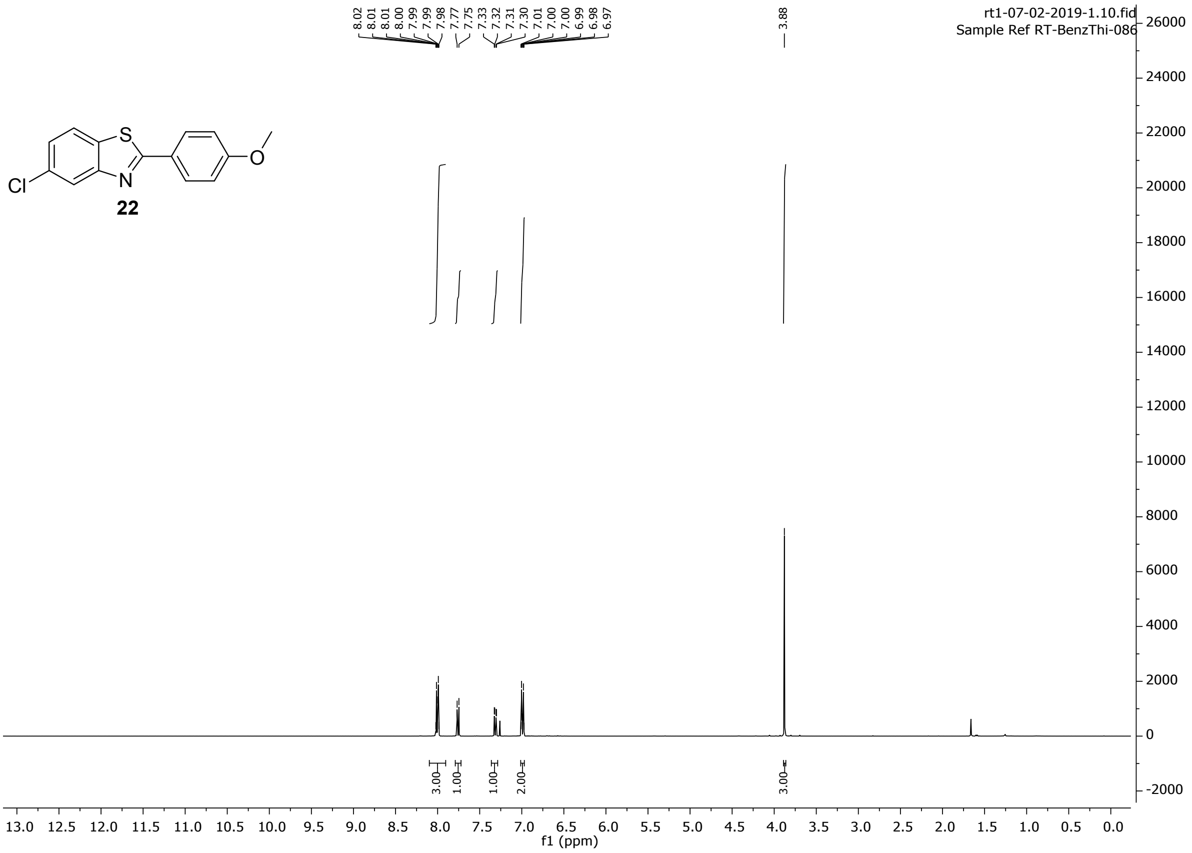


## $^{13}C\{1H\}$ NMR (101 MHz, Chloroform-*d*)

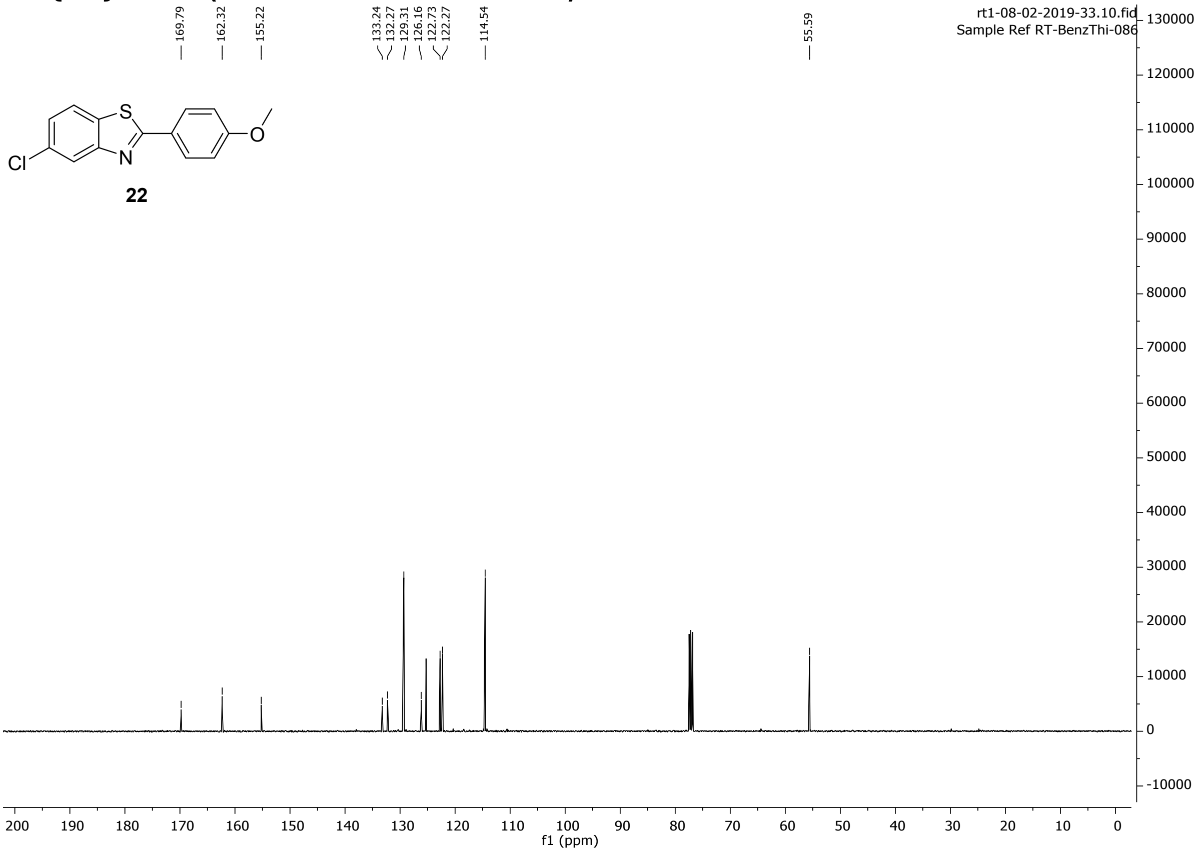

## $^1$H NMR (400 MHz, Chloroform-*d*)

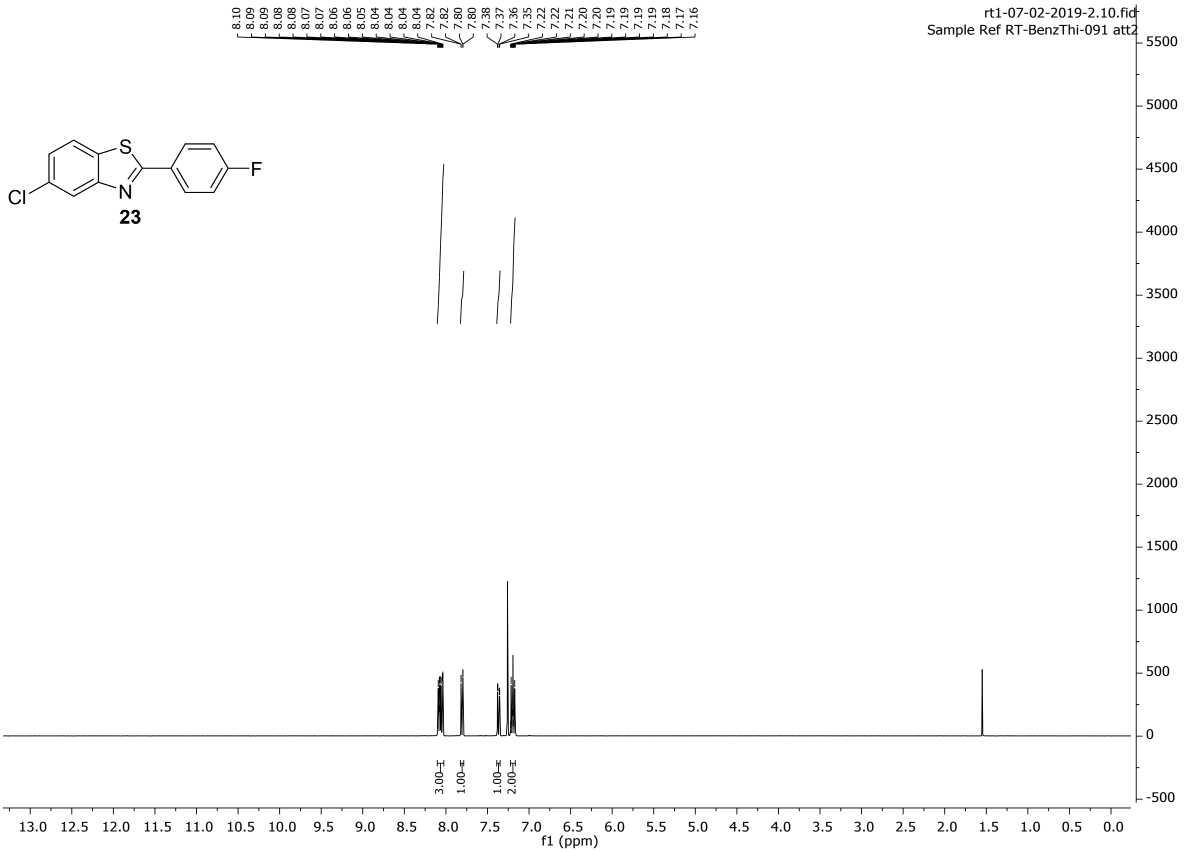


## $^{13}$C{1H} NMR (126 MHz, Chloroform-*d*)

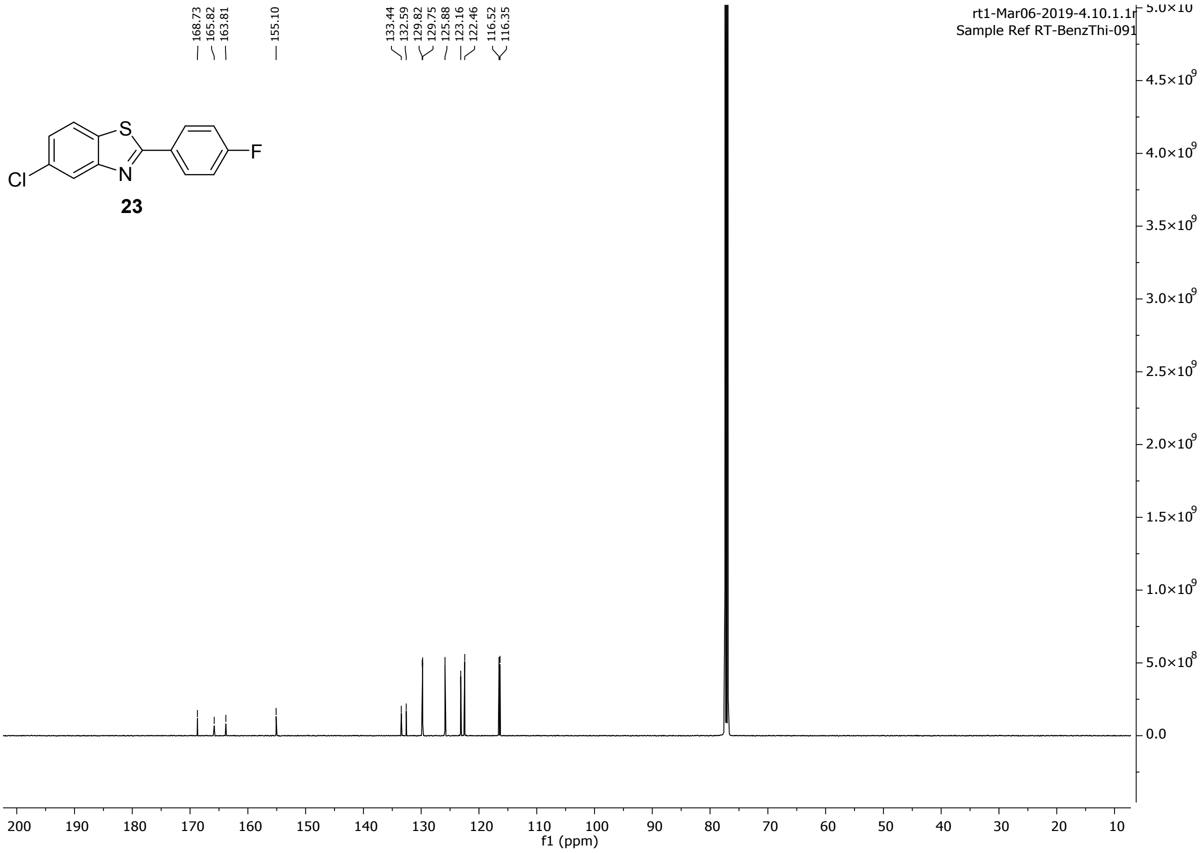

## S9.0. References


1. https://www.tinkercad.com/ Accessed 12th July 2023.
2. https://www.radleys.com/range/carousel-12-plus-reaction-station/?utm_source=Gpmax&utm_campaign=GoogleAds&creative=&keyword=&matchtype=&network=x&device=c&gclid=CjwKCAjwp9qZBhBkEiwAsYFsb9vOnkuI1cHFDe4L6q0LqvXI0HrbVcHCb15x5WRsMW0kYlBSH_7yJBoCffIQAvD_BwE Accessed 12th July 2023.
3. Hilton, S. T.; Penny, M. R.; Dos Santos, B. S.; Patel, B. *Br. Pat.,* GB201604322D0, (2016).
4. Penny, M. R.; Hilton, S. T. Design and Development of 3D Printed Catalytically-Active Stirrers for Chemical Synthesis *React. Chem. Eng.*,**5**, 853–858 (2020).
5. Herrera Cano, N. *et al.* Selective and eco-friendly procedures for the synthesis of benzimidazole derivatives. The role of the $Er(OTf)_3$ catalyst in the reaction selectivity. *Beilstein J. Org. Chem.* **12**, 2410–2419 (2016).
6. Chebolu, R., Kommi, D. N., Kumar, D., Bollineni, N. & Chakraborti, A. K. Hydrogen-bond-driven electrophilic activation for selectivity control: Scope and limitations of fluorous alcohol-promoted selective formation of 1,2-disubstituted benzimidazoles and mechanistic insight for rationale of selectivity. *J. Org. Chem.* **77**, 10158–10167 (2012).
7. Godugu, K. *et al.* Natural dolomitic limestone-catalyzed synthesis of benzimidazoles, dihydropyrimidinones, and highly substituted pyridines under ultrasound irradiation. *Beilstein J. Org. Chem.* **16**, 1881–1900 (2020).
8. Jamatia, R., Saha, M. & Pal, A. K. An efficient facile and one-pot synthesis of benzodiazepines and chemoselective 1,2-disubstituted benzimidazoles using a magnetically retrievable Fe3O4 nanocatalyst under solvent free conditions. *RSC Adv.* **4**, 12826–12833 (2014).
9. Ali A. Mohammadi, J. A. and N. K. Caro's Acid-Silica Gel Catalyzed Synthesis of 2-Aryl-1H-Benzimidazoles and 2-Aryl-1-arylmethyl-1H-benzimidazoles. *Heterocycles* **78**, 2337–2342 (2009).
10. Sadig, J. E. R., Foster, R., Wakenhut, F. & Willis, M. C. Palladium-catalyzed synthesis of benzimidazoles and quinazolinones from common precursors. *J. Org. Chem.* **77**, 9473–9486 (2012).

11. Thapa, P., Palacios, P. M., Tran, T., Pierce, B. S. & Foss, F. W. 1,2-Disubstituted Benzimidazoles by the Iron Catalyzed Cross-Dehydrogenative Coupling of Isomeric o-Phenylenediamine Substrates. *J. Org. Chem.* **85**, 1991–2009 (2020).
12. Rao, M. S. & Hussain, S. One-Pot, Borax-mediated synthesis of structurally diverse N, S-heterocycles in water. *Tetrahedron Lett.* **74**, 153159 (2021).
13. Dhawale, K. D., Ingale, A. P., Shinde, S. V., Thorat, N. M. & Patil, L. R. ZnO-NPs catalyzed condensation of 2-aminothiophenol and aryl/alkyl nitriles: Efficient green synthesis of 2-substituted benzothiazoles. *Synth. Commun.* **51**, 1588–1601 (2021).
14. Djuidje, E. N. *et al.* Design, synthesis and evaluation of benzothiazole derivatives as multifunctional agents. *Bioorg. Chem.* **101**, 103960 (2020).
15. Kaur, G., Moudgil, R., Shamim, M., Gupta, V. K. & Banerjee, B. Camphor sulfonic acid catalyzed a simple, facile, and general method for the synthesis of 2-arylbenzothiazoles, 2-arylbenzimidazoles, and 3H-spiro[benzo[d]thiazole-2,3′-indolin]-2′-ones at room temperature. *Synth. Commun.* **51**, 1100–1120 (2021).
16. Ye, L. miao *et al.* Visible-light-promoted synthesis of benzothiazoles from 2-aminothiophenols and aldehydes. *Tetrahedron Lett.* **58**, 874–876 (2017).
17. Gorepatil, P. B., Mane, Y. D., Gorepatil, A. B., Gaikwad, M. V. & Ingle, V. S. Samarium(III) triflate: A new catalyst for facile synthesis of benzothiazoles and benzoxazoles from carboxylic acids in aqueous media. *Res. Chem. Intermed.* **41**, 8355–8362 (2015).
18. Huang, J. *et al.* α-Keto Acids as Triggers and Partners for the Synthesis of Quinazolinones, Quinoxalinones, Benzooxazinones, and Benzothiazoles in Water. *J. Org. Chem.* **86**, 14866–14882 (2021).
19. Sung, G. H. *et al.* Eco-friendly atom-economical synthesis of 2-substituted-benzo[d]thiazoles and 2-substituted-benzo[d]oxazoles using 2-acylpyridazin-3(2H)-ones. *Tetrahedron* **69**, 3530–3535 (2013).
20. Monga, A., Bagchi, S., Soni, R. K. & Sharma, A. Synthesis of Benzothiazoles via Photooxidative Decarboxylation of α-Keto Acids. *Adv. Synth. Catal.* **362**, 2232–2237 (2020).
